# Arcopilins: A New Family of *Staphylococcus aureus* Biofilm Disruptors from the Soil Fungus *Arcopilus navicularis*

**DOI:** 10.1021/acs.jmedchem.4c00585

**Published:** 2024-08-14

**Authors:** Esteban Charria-Girón, Haoxuan Zeng, Tatiana E. Gorelik, Alexandra Pahl, Khai-Nghi Truong, Hedda Schrey, Frank Surup, Yasmina Marin-Felix

**Affiliations:** † Department Microbial Drugs, Helmholtz Centre for Infection Research (HZI), German Centre for Infection Research (DZIF), Partner Site Hannover-Braunschweig, Inhoffenstrasse 7, 38124 Braunschweig, Germany; ‡ Institute of Microbiology, Technische Universität Braunschweig, Spielmannstraße 7, 38106 Braunschweig, Germany; § Department Structure and Function of Proteins, Helmholtz Centre for Infection Research (HZI), Inhoffenstrasse 7, 38124 Braunschweig, Germany; ∥ Department Microbial Natural Products, Helmholtz-Institute for Pharmaceutical Research Saarland (HIPS), Campus E8.1, 66123 Saarbrücken, Germany; ⊥ Rigaku Europe SE, Hugenottenallee 167, 63263 Neu-Isenburg, Germany

## Abstract

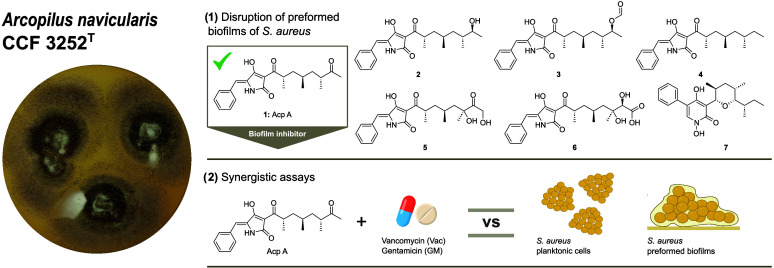

Biofilms represent a key challenge in the treatment of microbial infections; for instance, *Staphylococcus aureus* causes chronic or fatal infections by forming biofilms on medical devices. Herein, the fungus *Arcopilus navicularis* was found to produce a novel family of PKS-NRPS metabolites that are able to disrupt preformed biofilms of *S. aureus*. Arcopilins A–F (**1**–**6**), tetramic acids, and arcopilin G (**7**), a 2-pyridone, were elucidated using HR-ESI-MS and one-dimensional (1D) and two-dimensional (2D) nuclear magnetic resonance (NMR) spectroscopy. Their absolute configuration was established by the synthesis of MPTA-esters for **2**, analysis of ^1^H–^1^H coupling constants, and ROESY correlations, along with comparison with the crystal structure of **7**. Arcopilin A (**1**) not only effectively disrupts preformed biofilms of *S. aureus* but also potentiates the activity of gentamicin and vancomycin up to 115- and 31-fold times, respectively. Our findings demonstrate the potential application of arcopilins for the conjugated treatment of infections caused by *S. aureus* with antibiotics unable to disrupt preformed biofilms.

## Introduction

Sordarialean fungi, renowned for their pivotal ecological roles across various natural habitats, have emerged as instrumental contributors in different fields of economic relevance.^1−5^ This diverse taxonomic group is also a prolific source of biologically active secondary metabolites, from which taxa belonging to the Chaetomiaceae are particularly known to harbor a wealth of unique and chemically diverse entities.^4,6^ Despite the extensive research on fungi within this family, the exploration of untapped genera continues to offer opportunities for the discovery of novel natural products with diverse biological activities.

Over the past three decades, biofilms have been a relevant topic due to their complex nature and impact on human health. Biofilms are structured microbial communities, which adhere to any suitable living or abiotic surface through a self-produced matrix of extracellular polymeric substances (EPSs).^7^ The three-dimensional EPS matrix provides several functions within biofilms, such as the transportation of signals and nutrients between cells and the environment.^8−10^ In addition, biofilms confer protection against environmental factors, including high salt concentrations, ultraviolet radiation, extreme temperatures, pH variations, high pressure, and chemicals.^11−14^ As a result, biofilms also significantly enhance the tolerance and resistance of pathogens to antibiotics when compared to planktonic cells.^15^ According to a report by the National Institutes of Health (NIH), bacterial pathogens forming biofilms are responsible for 80% of the chronic infections in clinical trials.^16^ Among these pathogens, *Staphylococcus aureus*, recognized as an ESKAPE pathogen, is one of the most dangerous opportunistic organisms, causing a range of human infections.^17^ Numerous diseases, including osteomyelitis, cystic fibrosis, and otitis media, are therefore related to the biofilm infection of *S. aureus*.^18−20^

Microbial infections threaten the development of society, as their treatment remains a global challenge with the rapid increase and spread of resistance.^21−23^ To address this substantial challenge, combination therapy has been increasingly accepted in recent years, building on approaches established for anticancer treatment.^24^ This approach involves targeting multiple pathways within important pathogen biological processes, circumventing their defense mechanisms.^24^ For instance, the combination of sublethal concentrations of bacteriophages with the antibiotic vancomycin, or using biofilm-targeting antigens as a vaccine in conjunction with vancomycin, has significantly reduced *S. aureus* biofilm formation.^25,26^ These strategies are effective through the disruption of the biofilm structure or cell membrane, offering new avenues for therapeutic intervention.

During an ongoing project focused on the discovery of bioactive compounds from taxa belonging to the Sordariales, six previously undescribed tetramic acids (**1**–**6**) and a related 2-pyridone congener (**7**) were isolated from the soil-born fungus *Arcopilus navicularis* CCF 3252^T^. Herein, we report the isolation, structure elucidation, antimicrobial activities, and biofilm disruption properties against *S. aureus* of arcopilins A–G (**1**–**7**). Due to the remarkable efficacy of arcopilin A (**1**) to disrupt *S. aureus* biofilms at subtoxic concentrations, we decided to systematically examine its synergistic effect in combination with the known antibiotics, gentamicin (GM) and vancomycin (Vac), ineffective against the preformed biofilms of this pathogen.

## Results and Discussion

### Isolation and Structure Elucidation of Arcopilins

**Figure 1:**
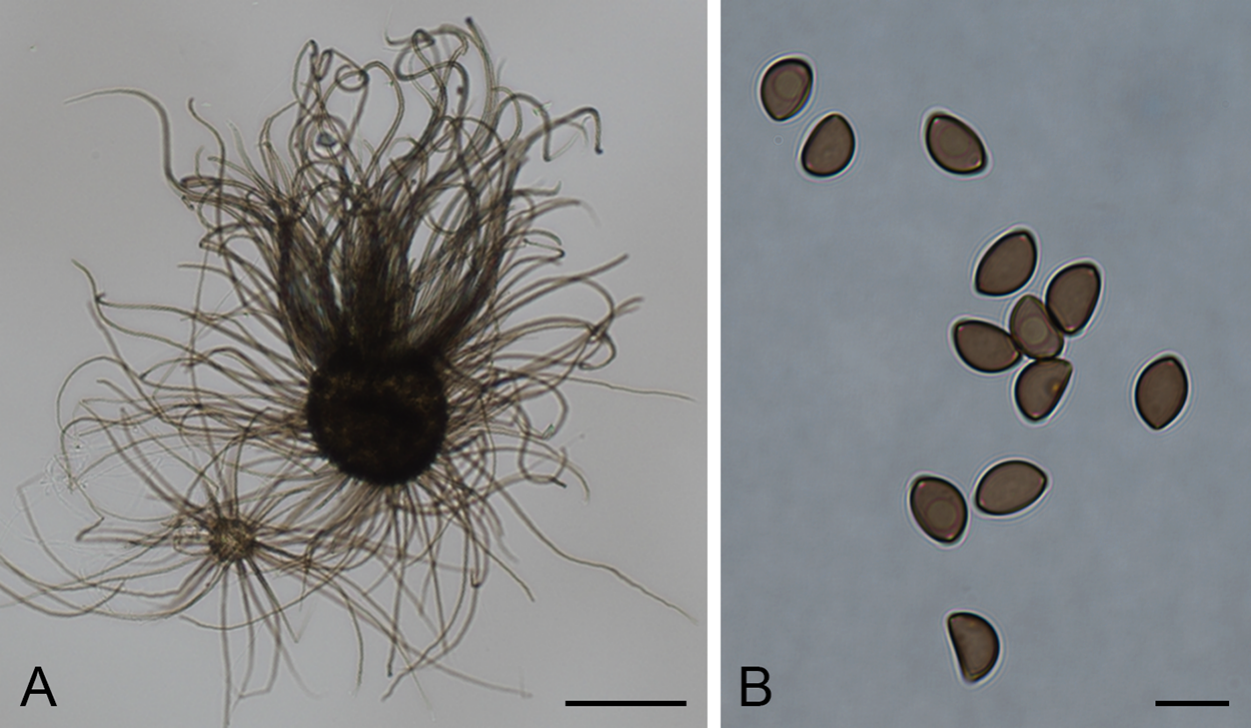
*Arcopilus navicularis* CCF 3252^T^. (A) Ascomata and (B) ascospores. Scale bars: 10 μm.

The strain CCF 3252^T^ was obtained from the Culture Collection of Fungi (CCF) in Prague. This strain represents the type strain of *A. navicularis*.^27^ Morphologically, this species is characterized by ascomata bearing arcuate hairs with incurved to coiled apexes and navicular ascospores with two apical germ pores (Figure 1).

The production of secondary metabolites by the chaetomiaceous fungus *A. navicularis* CCF 3252^T^ was evaluated under its cultivation in three different liquid media (YM 6.3, ZM 1/2, Q6 1/2) and one solid medium (BRFT) (Figure S1). Metabolomic analysis of the obtained crude extracts by high-resolution electrospray ionization mass spectrometry (HR-ESI-MS) discerned the production of nitrogen-containing molecules with unprecedented molecular formulas and a distinctive UV/vis absorption at λ_max_ 226, 288, and 346 nm in the Q6 1/2 medium. After the scaled-up fermentation of *A. navicularis* CCF 3252^T^ in Q6 1/2 medium (8 L), targeted isolation by preparative HPLC afforded compounds **1**–**6** as brown to orange oils and **7** as an orange to white powder (Figure 2). Their planar structures were elucidated by 1D and 2D NMR spectroscopy in combination with tandem mass spectrometry analyses (Table 1; Figures S4–S45).

**Figure 2:**
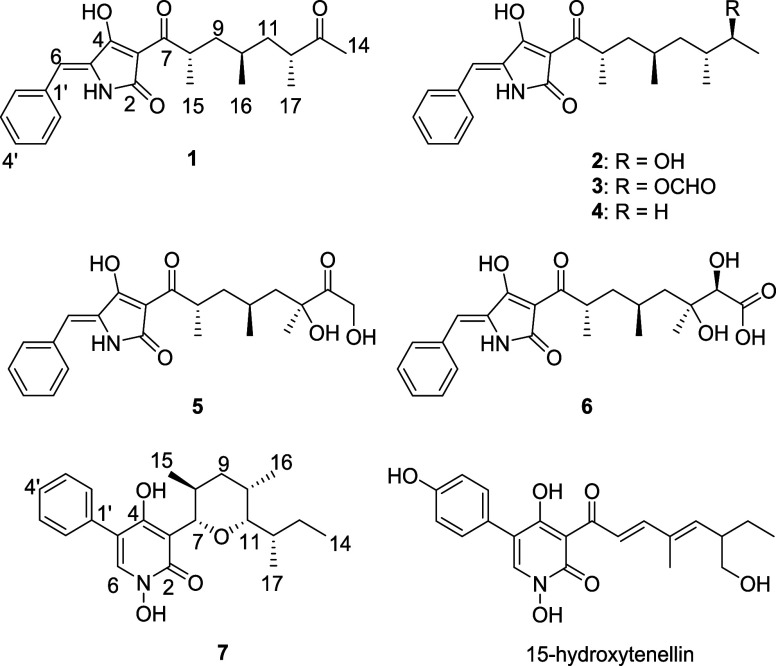
Chemical structures of the tetramic acids arcopilins A−F (**1**–**6**) and the related 2-pyridone arcopilin G (**7**), as well as 15-hydroxytenellin, a 2-pyridone produced by the entomopathogenic fungus *Beauveria neobassiana*.

**Table 1:** NMR Data of **1**–**7** in DMSO (^1^H 700 MHz, ^13^C 175 MHz)

	1		2		3		4		5		6		7	
#	δ_C_, mult.	δ_H_, mult.	δ_C_, mult.	δ_H_, mult.	δ_C_, mult.	δ_H_, mult.	δ_C_, mult.	δ_H_, mult.	δ_C_, mult.	δ_H_, mult.	δ_C_, mult.	δ_H_, mult.	δ_C_, mult.	δ_H_, mult.
2	n.o.		n.o.		n.o.		n.o.		n.o.		n.o.		157.2, C	
3	n.o.		n.o.		n.o.		n.o.		n.o.		n.o.		109.8, C	
4	181.2^a^, C		n.o.		181.4, C		181.4, C		n.o.		n.o.		159.7, C	OH: 9.54, s
5	n.o.		n.o.		n.o.		n.o.		n.o.		n.o.		111.0, C	
6	107.7^a^,^b^, CH	6.44, br s	108.9^b^, CH	6.37, br s	107.4, CH	6.41, br s	107.7, CH	6.45, br s	n.o.	6.40, br s	107.5^b^, CH	6.41, br s	133.9, CH	7.87, s
7	n.o.		n.o.		n.o.		n.o.		n.o.		n.o.		81.5, CH	4.61, d (9.6)
8	n.o.	3.79, tq (7.2,6.7)	n.o.	3.84, tq (7.5,6.5)	n.o.	3.84, m	34.6^b^, CH	3.82, tq (7.2,6.8)	n.o.	3.73, tq (7.3,6.8)	n.o.	3.73, m	30.1, CH	1.99, m
9	40.6, CH_2_	1.54, m	41.3, CH_2_	1.56, m	41.4, CH_2_	1.59, m	41.3, CH_2_	1.54, m	41.3, CH_2_	1.45, m	42.0, CH_2_	1.47, m	39.6^b^, CH_2_	1.70, dt (13.2, 3.0)
		1.34, m		1.26, m		1.31, m		1.30, m		1.41, m		1.39, m		1.56, m
10	28.1, CH	1.39, m	27.5, CH	1.41, m	27.6, CH	1.43, m	27.8, CH	1.46, m	26.3, CH	1.63, m	26.2, CH	1.67, m	28.5, CH	1.93, m
11	39.0, CH_2_	1.36, m	39.2, CH_2_	1.08, m	38.5, CH_2_	1.17,ddd (13.5,10.3,3.6)	43.4, CH_2_	1,11, m	46.4, CH_2_	1.55, dd (14.0,7.8)	44.2, CH_2_	1.47, m	84.7, CH	9.31, m
		1.26, m				1.06, m		1.01, m		1.49, dd (14.0,4.8)		1.29,dd (14.3,7.4)		
12	43.9, CH	2.51, m	36.4, CH	1.43, m	34.1, CH	1.69, m	31.1, CH	1.34, m	78.1, C	OH: 5.07, s	73.5, C		35.4, CH	1.45, m
13	211.8, C		69.9, CH	3.37, dq (6.3,5.0)	73.9, CH	4.74, m	19.9, CH_2_	1.22, m	215.8, C		77.3, CH	3.70, s	25.2, CH_2_	1.58, m
					13-OCHO:	8.18, s		1.09, m						
					161.8, CH									
14	27.7, CH_3_	2.08, s	19.0, CH_3_	0.93, d (6.3)	15.8, CH_3_	1.101, d (6.5)	11.2, CH_3_	0.81, t (7.3)	64.5, CH_2_	4.45, d (19.6)	174.1, C		10.5, CH_3_	0.80, t (7.5)
										4.40, d (19.6)				
15	17.0, CH_3_	1.08, d (6.7)	17.3, CH_3_	1.07, d (6.5)	17.3, CH_3_	1.096, d (6.5)	17.1, CH_3_	1.10, d (6.9)	16.7, CH_3_	1.05, d (6.8)	16.7, CH_3_	1.08, d (6.9)	17.7, CH_3_	0.75, d (6.4)
16	19.4, CH_3_	0.84, d (6.2)	19.4, CH_3_	0.80, d (6.5)	19.3, CH_3_	0.82, d (6.5)	19.6, CH_3_	0.82, d (6.6)	20.6, CH_3_	0.77, d (6.6)	21.6, CH_3_	0.94, d (6.5)	11.9, CH_3_	0.94, d (6.9)
17	15.5, CH_3_	0.89, d (6.9)	13.8, CH_3_	0.64, d (6.8)	13.9, CH_3_	0.72, d (6.7)	18.6, CH_3_	0.70, d (6.6)	26.7, CH_3_	1.12, s	22.8, CH_3_	1.05, s	13.9, CH_3_	0.78, d (6.7)
1′	133.3, C		133.4^a^, C		133.1, C		133.3, C		133.4, C		133.5, C		133.3, C	
2′/ 6′	129.6, CH	7.63, br d (7.7)	129.4, CH	7.62, br d (7.6)	129.6, CH	7.63, br d (7.6)	129.6, CH	7.63, br d (7.6)	129.5, CH	7.62, br d (7.6)	129.5, CH	7.62, br d (7.6)	129.1, CH	7.46, br d (7.6)
3′/ 5′	128.7, CH	7.40, br t (7.7)	128.7, CH	7.39, br t (7.6)	128.7, CH	7.39, br t (7.6)	128.7, CH	7.39, br t (7.6)	128.7, CH	7.39, br t (7.6)	128.7, CH	7.39, br t (7.6)	128.2, CH	7.38, br t (7.6)
4′	128.2, CH	7.32, br t (7.7)	127.9, CH	7.30, br t (7.6)	128.2, CH	7.31, br t (7.6)	128.2, CH	7.32, br t (7.6)	128.0, CH	7.30, br t (7.6)	128.1, CH	7.31, br t (7.6)	127.1, CH	7.31, br t (7.6)

a Chemical shift extracted from HMBC data.

b Chemical shift extracted from HSQC data; n.o.: not observed.

The molecular formula of compound **1** was determined as C_22_H_27_NO_4_ according to the quasimolecular ion peak cluster at *m*/*z* 370.2015 [M + H]^+^ in the HR-ESI-MS spectrum, indicating ten degrees of unsaturation. ^1^H and HSQC spectra revealed the presence of four methyl, two methylene, and four olefinic/aromatic signals, two of the aromatics with dual intensities. Since the ^13^C NMR spectrum only contained signals for an additional ketone and a further quaternary carbon without bound protons, signals of five carbon atoms were missing according to the molecular formula. HMBC correlations connected a styryl and an oxotrimethyleptyl moiety as two isolated parts of the molecule (Figure S8). Based on the coupling of 6–H to N–1 in the ^1^H,^15^N HMBC spectrum, four unassigned degrees of unsaturation, and chemical shifts, we deduce the tetramic acid backbone for **1**. Tetramic acids are known for their tautomeric exchange, explaining the missing signals in the NMR spectra. The rather small shift difference of the germinal methylene protons of Δδ_H_ = 0.20 and 0.10 ppm for 9–H_2_ and 11–H_2_, respectively, is indicative of a *trans*/*trans* configuration of the methyl groups.^28^

The molecular formula of **2** was established as C_22_H_29_NO_4_ according to the quasimolecular ion peak cluster at *m*/*z* 372.2168 [M + H]^+^ in the HR-ESI-MS spectrum, corresponding to the loss of one degree of unsaturation compared to **1**. NMR data were highly similar to those of **1**, with the replacement of the C–13 keto moiety by a hydroxyl. A J-resolved analysis connected the stereochemistry of C–12 and C–13,^29^ while the patterns of the Δδ^SR^ shift with a negative value for 14–H_3_ (−0.09) and positive ones for 12–H (+0.04) and 17–H_3_ (+0.08) were indicative for an 8*S*,10*R*,12*R*,13*S* absolute configuration.^30^

Compounds **3** and **4** were found to be the 18–formyl and 18–dehydroxy derivatives of **2**, respectively. Indicative for the structures were the molecular formulas C_23_H_29_NO_5_ and C_22_H_29_NO_3_, respectively, in addition to the additional formyl group connected to C–13 by HMBC coupling in **3** as well as the lack of signals for the hydroxyl function at C–13 in **4**. HR-ESI-MS data revealed C_22_H_27_NO_6_ as the molecular formula of compound **5**, meaning two additional oxygen atoms compared to **1**. These were located at C–12 and C–14, as demonstrated by the replacement of the methyl group CH_3_–14 as well as methane CH–12 by an oxymethylene as well as a carbon devoid of bound protons. Since ROESY correlations and coupling constants remained largely unchanged, we ascribe **3** as the 8*S*,10*S*,12*S* configuration. HR-ESI-MS data disclosed the molecular formula C_22_H_27_NO_7_ for **6**. In the structure of **6**, methyl C-14 and methine C–12 of **1** were replaced by a carboxylic acid and an oxygenated carbon devoid of bound protons, respectively. Coupling constants and ROESY correlations are similar to those of **3**, and thus, we assign a common 8*S*,10*S*,12*S*,13*R* configuration.

Compound **7** had the same molecular formula C_22_H_29_NO_4_ as **2**. However, NMR data showed apparent differences. The methane CH–6 was significantly deshielded (δ_H_ 7.86/ δ_C_ 133.6) compared to compounds **1**–**6**, and all expected carbons were observed in the ^13^C NMR spectrum, indicating a strongly lesser degree of tautomerism. The same styryl and 6-keto-1,3,5-trimethyleptyl moieties were assembled by COSY and HMBC data, but HMBC correlations from 4–OH to C-3, C-4, and C-5 and from 6–H to C-2, C-4, and C-5 connected the α-pyridone. Strong ROESY correlations between 7–H and 11–H as well as 8–H and 16–H_3_ established the 7*S*,8*S*,10*S*,11*S* stereochemistry.

**Figure 3:**
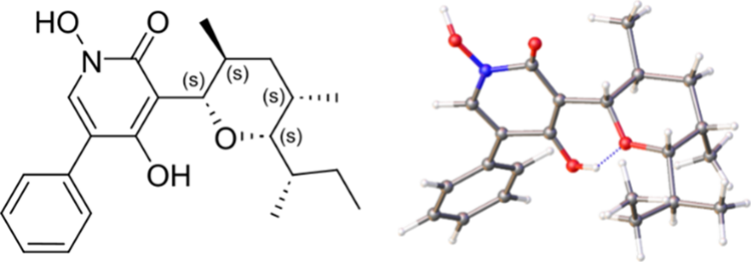
Absolute configuration of the stereocenters and the molecular conformation of compound **7** within its crystal structure determined from 3D ED analysis.

The crystal structure of compound **7** was determined via a continuous rotation 3D electron diffraction (3D ED) experiment collected on a XtaLAB Synergy-ED diffractometer.^31^ The structure was solved with direct methods,^32^ and the absolute configuration was determined in the course of dynamical refinement in JANA.^33,34^ The absolute configuration of the stereocenters, as well as the molecular conformation within the crystal structure, is shown in Figure 3. The experimental and refinement details as well as the CSD deposition number of the structure are given in the Supporting Information. Arcopilin G (**7**) is nearly the enantiomer of septoriamycin A, which has been isolated from a culture medium of the ascomycete fungus *Septoria pistaciarum*.^35^ A total synthesis of septoriamycin A has been completed by Fotiadou and Zogrofos.^36^

Extensive knowledge of the biosynthesis of tetramic acids and their related 2-pyridones reveals a common progression catalyzed by polyketide synthase-nonribosomal peptide synthetase (PKS-NRPS) hybrid machineries. The diversity and evolution of these biosynthetic pathways are illustrated in several natural products, including tenellin, aspyridone A, fusarin C, leporin B, fischerin, PFF1140, sambutoxin, equisetin, etc.^37,38^ In the late stages of tenellin biosynthesis, two cytochrome P450 oxidases are responsible for catalyzing the oxidative expansion and N-hydroxylation of pretenellin A.^38^ Furthermore, certain metabolites might undergo cyclization of their side chains through processes such as inverse-electron demand Diels–Alder reactions, as seen in the antifungal ilicicolin H, or through a Michael addition, as observed in the biosynthesis of the mycotoxin (-)-sambutoxin.^39,40^ Since compounds **1**–**7** share the same carbon skeleton except for **3**, it is likely that **7** is biosynthesized in a similar fashion as (-)-sambutoxin, a related PKS-NRPS hybrid product with a longer polyketide chain.

### Antimicrobial and Cytotoxic Activities of Arcopilins

The antimicrobial activities of compounds **1**–**7** (Acp A–G) were assessed against different bacterial and fungal strains in addition to their cytotoxic effects on two mammalian cell lines. The tested microorganisms comprised a diverse array of clinically relevant pathogens, encompassing sensitive indicator strains. Among the Gram-positive bacteria were *Bacillus subtilis*, *Staphylococcus aureus*, and *Mycolicibacterium smegmatis*. Gram-negative bacteria included *Acinetobacter baumannii*, *Chromobacterium violaceum*, *Escherichia coli*, and *Pseudomonas aeruginosa*. Additionally, filamentous fungi such as *Mucor hiemalis* and yeasts including *Candida albicans*, *Wickerhamomyces anomalus*, *Rhodotorula glutinis*, and *Schizosaccharomyces pombe* were included. Generally, all compounds presented similar biological properties, summarized in weak or no activity against fungal pathogens as well as weak to moderate inhibition of Gram-positive bacteria (Table 2). Acp E and F did not exhibit any antimicrobial activity in our assays.

**Table 2:** Minimum Inhibitory Concentration (MIC, μg/mL) Against Bacterial and Fungal Test Organisms and Half-Maximal Inhibitory Concentrations (IC_50_, μg/mL) against Mammalian Cell Lines of Arcopilins A–G. Reference Compounds: (a) Oxytetracycline, (b) Gentamicin, (c) Ciprofloxacin, (d) Kanamycin, (e) Nystatin, and (f) Epothilone B. Notes: No Activity Observed under Test Conditions (−), Not Tested (n.t.)

		Acp							
tested organisms/cell line	code	A	B	C	D	E	F	G	ref
MIC against bacteria (μg/mL)									
*B. subtilis*	DSM 10	66.7	66.7	33.3	4.2	–	–	8.3	8.3^a^
*E. coli*	DSM 1116	–	–	–	–	–	–	–	1.7^b^
*P. aeruginosa*	PA 14	–	–	–	–	–	–	–	0.21^b^
*S. aureus*	DSM 346	66.7	66.7	33.3	–	–	–	16.6	0.4^b^
*S. aureus*	DSM 1104	31.3	n.t.	n.t.	n.t.	n.t.	n.t.	n.t.	15.6^c^
*C. violaceum*	DSM 30191	–	–	–	–	–	–	–	0.42^b^
*A. baumannii*	DSM 30008	–	–	–	–	–	–	–	0.26^c^
*M. smegmatis*	ATCC 700084	–	–	–	66.7	–	–	–	1.7^d^
MIC against fungi (μg/mL)									
*W. anomalus*	DSM 6766	–	–	–	–	–	–	–	8.3^e^
*S. pombe*	DSM 70572	66.7	–	–	–	–	–	–	4.2^e^
*C. albicans*	DSM 1665	66.7	–	–	–	–	–	–	8.3^e^
*M. hiemalis*	DSM 2656	66.7	–	66.7	66.7	–	–	–	8.3^e^
*R. glutinis*	DSM 10134	–	–	–	–	–	–	–	4.2^e^
IC_50_ against mammalian cell lines (μg/mL)									
KB-3-1	ACC 158	8.9	–	0.8	0.8	–	–	2.0 × 10^–4^	8.6 × 10^–6 f^
L929	ACC 2	14.0	–	1.4	1.7	–	–	2.4 × 10^–5^	8.6 × 10^–5 f^

The above suggests that the hydroxylation at C-12 and C-14 in Acp E has a negative effect on antibacterial activity. Similarly, the hydroxylation at C-12 and C-13, in addition to the presence of carboxylic acid at C-14 in Acp F, results in the loss of antibacterial activity. In terms of their cytotoxic properties, note that 2-pyridone Acp G was the most cytotoxic metabolite, while its tetramic acid congeners presented rather weak or no cytotoxic effects as for compounds Acp B, Acp E, and Acp F. The fact that hydrophilic arcopilins are less cytotoxic suggests a possible correlation between the hydrophobicity and the cytotoxicity of these molecules.

While PKS-NRPS hybrid products within the tetramic acid and pyridone secondary metabolite families exert a wide range of biological activities and are widespread in ascomycetes, only a limited number of examples from the Sordariales order have been reported. Notably, the most notorious examples are the decalin-containing tetramic acids, myceliothermophins, originally discovered in *Thermothelomyces thermophilus* (syn. *Myceliophthora thermophila*).^41^ The potent antitumor activity exhibited by myceliothermophins C, D, and E against a number of human cancer cell lines has prompted numerous total synthesis endeavors.^42,43^ Similarly, the chaetolivacines A–C, isolated from *Chaetomium olivaceum* (Chaetomiaceae), represent another example of decalin-containing tetramic acids.^44^ Only chaetolivacine B exerts moderate antibacterial properties against *S. aureus* and methicillin-resistant *S. aureus* (MRSA). Additionally, rare decalin-containing tetramic acids such as zopfiellamide A and B, as well as zopfielliamides A–D, have been isolated from *Zopfiella latipes* and *Zopfiella* sp., taxa with uncertain taxonomic placement within this order.^4,45,46^

### Arcopilins Are Able to Disrupt the Preformed Biofilms of *Staphylococcus aureus*

**Figure 4:**
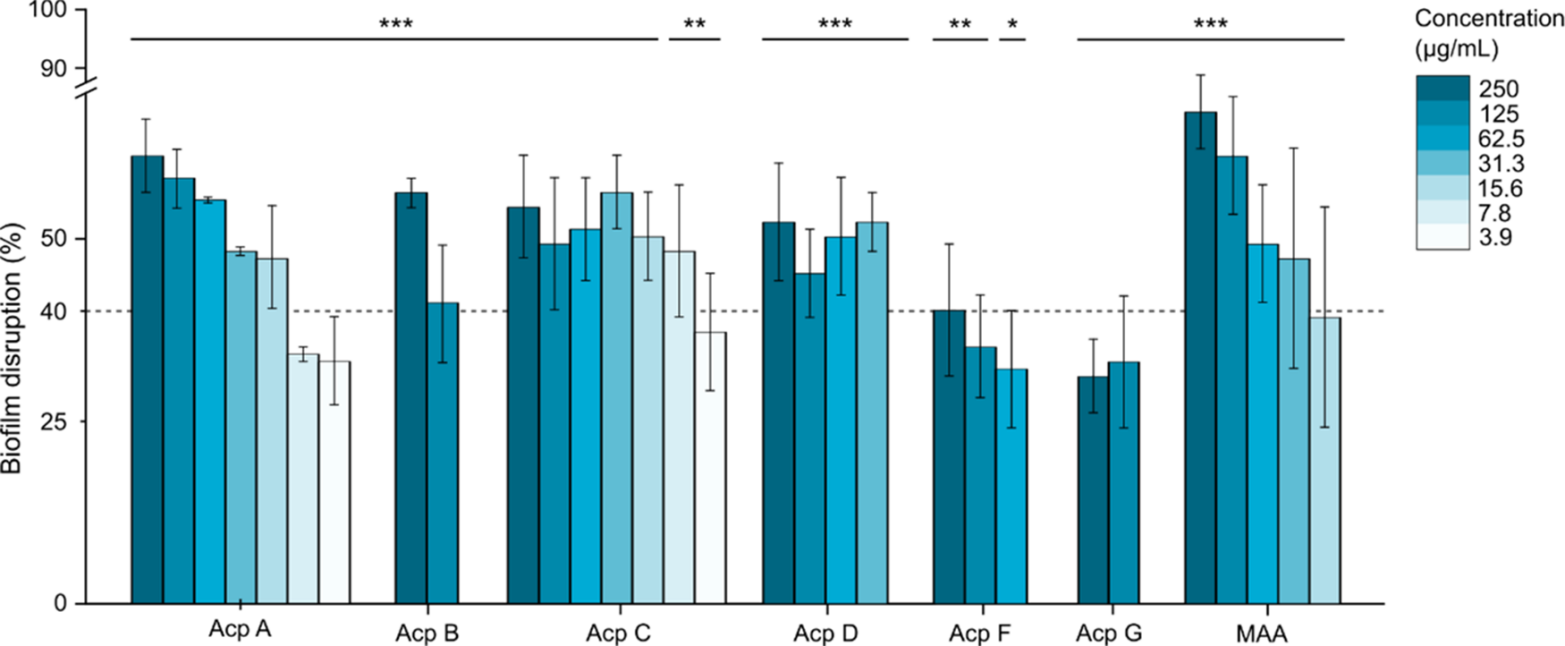
Effects of Acp A–G on preformed biofilms of *S. aureus* DSM 1104 compared to the positive control (MAA). Error bars indicate the standard deviation. *p*-values: * *p* < 0.05, ** *p* < 0.01, and *** *p* < 0.001, (*n* = 4). The dotted line represents 40% of biofilm disruption, considered a threshold for prioritization of potent molecules.

After identifying that arcopilins exhibit rather weak activities against the tested organisms and cell lines, we decided to evaluate their efficacy toward the disruption of preformed biofilms of the bacterial pathogen *S. aureus*. Therefore, Acp A–G were evaluated against preformed biofilms of *S. aureus* using crystal violet staining.^47^ The 2-pyridone, 15-hydroxytenellin (15-Ht), produced by the entomopathogenic fungus *Beauveria neobassiana* was also used for comparison, as the tenellins are model compounds for the study of fungal secondary metabolite biosynthesis and have displayed inhibitory properties against the formation of biofilms by *S. aureus*.^38,48^ Among the tested metabolites, Acp A and C showed the most promising disrupting effects toward preformed biofilms of *S. aureus*, whereas weak to moderate effects were observed for Acp B, F, and G (Figure 4).

Furthermore, Acp G (Figure 4) and 15-Ht (data not shown), both belonging to the class of 2-pyridones, were not active against preformed biofilms of *S. aureus*. Acp A displayed approximately 50–60% efficacy toward preformed biofilms within the concentration range of 15.6 to 250 μg/mL. Similarly, Acp C demonstrated ca. 50% effectiveness in the dispersal of preformed biofilms between 7.8 μg/mL and 250 μg/mL. Notably, both compounds exhibited a pronounced efficacy of 35–45% even at a concentration as low as 3.9 μg/mL. These results align with the growth curve of Acp A shown in Figure S47, demonstrating that the growth of *S. aureus* was inhibited by Acp A treatment even at concentrations as low as 3.9 and 7.8 μg/mL. Consequently, the disruption of existing biofilms may be due to the downregulation of cell growth. However, the precise mechanism behind this effect remains unclear and it is out of the scope of the present study.

In the case of Acp A and C, a carbonyl group is present on the side chain of these metabolites. However, the presence of this moiety is not exclusively necessary for the observed activity, as demonstrated by Acp D, which lacks a carbonyl group but still exhibits significant dispersal effects at concentrations as low as 31.3 μg/mL. During our examination of different tetramic acids and related 2-pyridones, no discernible link between cytotoxicity and the dispersion of *S. aureus* preformed biofilms was found. For instance, Acp G, the most cytotoxic metabolite within the tested congeners, exhibited only weak disruptive effects on the biofilms. A link between cytotoxicity and biofilm eradication could affect the applicability of the metabolites, as increased cytotoxicity might also damage host cells.

### Synergistic Effects of Arcopilin A in Combination with Gentamicin and Vancomycin

Interestingly, both Acp A and Acp C demonstrated remarkable effectiveness in disrupting preformed biofilms of *S. aureus*. Given its promising activity and relatively low cytotoxicity, we selected Acp A for further experiments. We investigated its in-depth effects alone and in combination with the antibiotics gentamicin (GM) and vancomycin (Vac) on planktonic cells and *S. aureus* biofilms. To evaluate the influence of Acp A on both biofilm metabolic activity and planktonic cell growth, XTT and growth curve analyses were conducted, respectively. The results from XTT assay as depicted in Figure S46 revealed a significant reduction in metabolic activity even at low concentrations of 3.9 μg/mL. These findings were consistent with the outcomes of the antibiofilm assay, indicating that effective concentrations of Acp A in dispersing *S. aureus* biofilms coincide with an alteration in the metabolic activity of preformed biofilms. In line with this, inhibitory effects were observed at concentrations between 7.8 μg/mL and 2 μg/mL according to the growth curve analysis (Figure S47).

After assessing the effects of Acp A on the pathogen *S. aureus*, we delved deeper into the interaction of Acp A with established antibiotics (GM and Vac). This exploration focused on both planktonic cells and preformed biofilms of *S. aureus*. Consequently, we used a checkerboard assay to determine the fractional inhibitory concentration index (FICI) for combinations involving Acp A, GM, or Vac based on both their MIC values in combination.^49^

**Figure 5:**
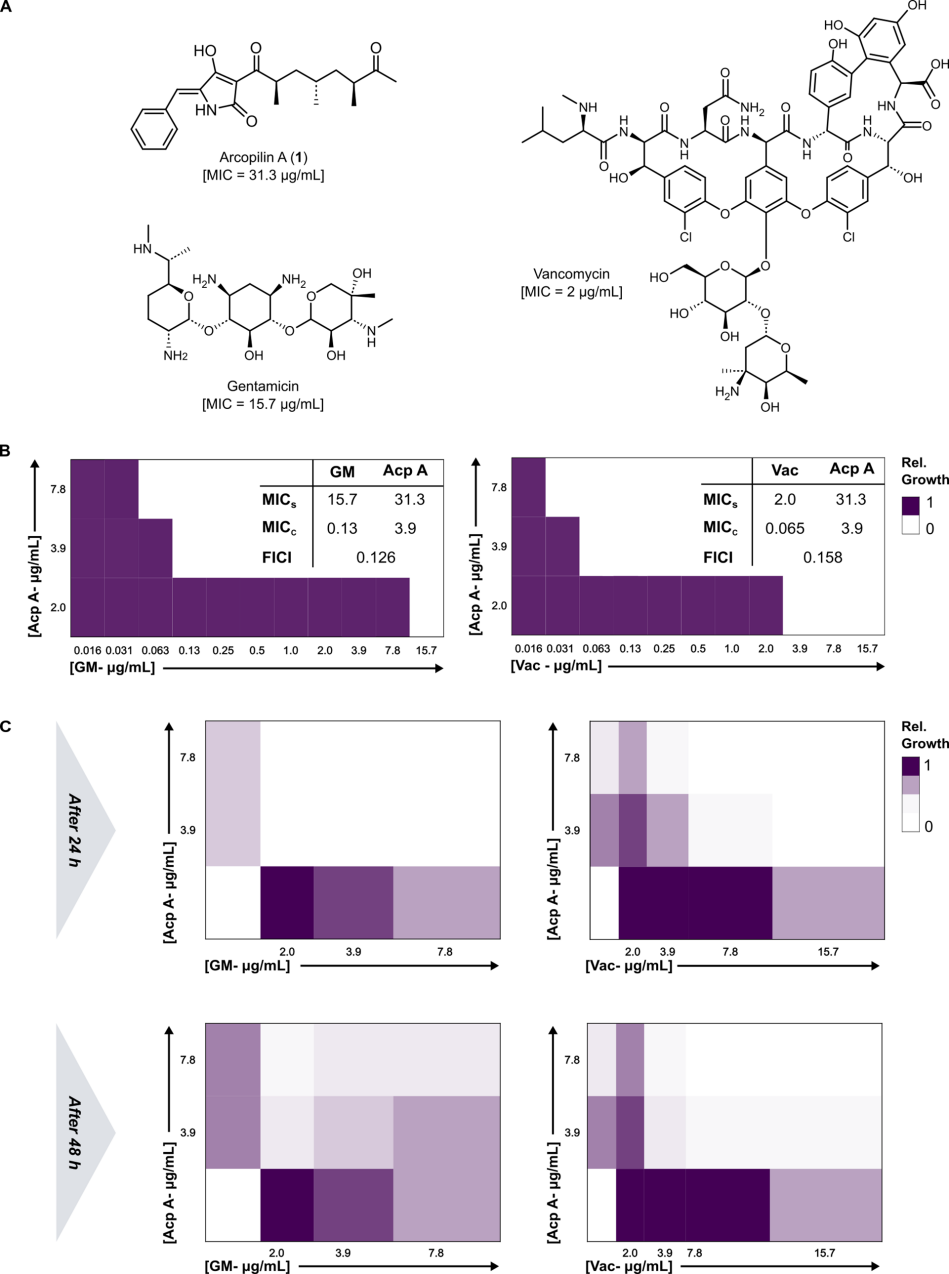
(A) Chemical structures of arcopilin A (Acp A), gentamicin (GM), and vancomycin (Vac) used for synergistic assays. (B) Checkerboard assay as demonstration of the synergistic effects of Acp A and GM, as well as Acp A and Vac on planktonic cells of *S. aureus* DSM 1104 (FICI < 0.5: synergistic). MIC_s_ refers to the MIC value of each single compound, while MIC_c_ refers to the MIC value of each compound in combination with (C) CFU count analysis as demonstration of the synergistic effects of Acp A and GM, as well as Acp A and Vac on preformed biofilms of *S. aureus* DSM 1104 at 24 and 48 h. Relative growth refers to the normalized CFU counting (CFU count in each treatment and CFU count in the negative control).

Antibiotics commonly used to fight bacterial infections often act through diverse mechanisms to hinder the growth of these pathogens. For instance, the well-known antibiotic GM functions as a protein synthesis inhibitor, while Vac exerts inhibitory effects on this pathogen by interfering with cell wall biosynthesis. Our findings revealed that when Acp A (3.9 μg/mL) was used in combination with GM or Vac, the MIC values of the established antibiotics were significantly decreased from 15.6 to 0.13 μg/mL and from 2 to 0.065 μg/mL, respectively. The combination treatment substantially increased the potency of GM and Vac up to 115-fold and 31-fold, respectively, and calculation of the FICI showed synergistic effects (FICI < 0.5) for both combinations (Figure 5b). Similarly, the MIC value of Acp A decreased almost 10-fold when combined with each antibiotic. Additionally, combined effects were also assessed on the preformed biofilms. Consequently, a colony-forming unit (CFU) count analysis treated with Acp A (7.8–3.9 μg/mL), GM (7.8–2 μg/mL), or Vac (15.6–3.9 μg/mL) alone, as well as their combinations, was carried out for preformed biofilms. For both GM and Vac, roughly a 3-fold improvement in the inhibitory effects was observed when used in combination with Acp A (7.8–3.9 μg/mL) (Figure 5c).

According to previous studies, tetramic acids with long polyketide side chains, such as the reutericyclins, have been shown to act against bacteria by disrupting their proton gradient and membrane potential.^50,51^ The cellular membrane potential is dynamic, and it is linked to signal transmission between cells within biofilms and the overall level of biofilm formation. In addition, tetramic acids are likely to act as metal chelators, but the biological implications of this phenomenon are poorly understood.^52^ Similarly, it has been demonstrated that human-targeted drugs, when used at sublethal concentrations, can be repurposed as new antimicrobials in combination therapy.^53^ However, the specific mode of action by which arcopilins disrupt *S. aureus* biofilms remains unclear and will require future investigation.

## Conclusions

In summary, we discovered a new family of tetramic acids and related 2-pyridones named arcopilins, adding to the diversity of this class of natural products. While their antimicrobial properties against the tested microorganisms were relatively weak, these compounds exerted varying effectiveness at disrupting preformed biofilms of *S. aureus*. Among them, arcopilin A (**1**) emerged as a particularly promising candidate for an in-depth investigation of its effects on this bacterial pathogen solely and in combination with established antibiotics like gentamicin and vancomycin. Notably, arcopilin A exhibited synergistic effects on both planktonic cells and preformed biofilms of *S. aureus* when paired with two antibiotics that operate through different modes of action. These findings suggest the potential for arcopilin A to be further developed for potent preclinical applications in combination therapy.

## Experimental Section

### Fermentation, Extraction, and Isolation

For the evaluation of the production of secondary metabolites by *Arcopilus navicularis* CCF 3252^T^, three different liquid media (YM 6.3: malt extract 10 g/L, yeast extract 4 g/L, d-glucose 4 g/L, pH 6.3 before autoclaving; ZM 1/2: molasses 5 g/L, oatmeal 5 g/L, sucrose 4 g/L, mannitol 4 g/L, d-glucose 1.5 g/L, CaCO_3_ 1.5 g/L, edamine 0.5 g/L, (NH_4_)_2_SO_4_ 0.5 g/L, pH 7.2 before autoclaving; Q6 1/2: d-glucose 2.5 g/L, glycerin 10 g/L, cotton seed flour 5 g/L, pH 7.2 before autoclaving) and one solid medium (BRFT: brown rice 28 g) as well as 0.1 L of base liquid (yeast extract 1 g/L, disodium tartrate dihydrate 0.5 g/L, KH_2_PO_4_ 0.5 g/L) were used. The fungus was grown in yeast malt agar (YM agar: malt extract 10 g/L, yeast extract 4 g/L, d-glucose 4 g/L, agar 20 g/L, pH 6.3 before autoclaving) at 23 °C. Later, the colonies were cut into small pieces using a cork borer (1 cm × 1 cm) and eight pieces were placed into 500 mL Erlenmeyer flasks containing 200 mL of each liquid medium, which were incubated at 23 °C under shaking conditions (140 rpm) in the darkness until 3 days after glucose depletion. For the solid culture, an additional 500 Erlenmeyer flask containing 200 mL of YM broth was incubated at 23 °C under shaking conditions (140 rpm) in the darkness. After 7 days, 6 mL of this seed culture was transferred to an Erlenmeyer flask of 500 mL containing the BRFT medium. This solid culture was incubated for 15 days at 23 °C in the darkness without agitation.

To extract the secondary metabolites from the liquid cultures, the mycelia were initially separated from the supernatant through filtration. The supernatant was extracted with an equal volume of ethyl acetate in a separatory funnel. The resulting ethyl acetate fraction was evaporated to dryness under vacuum at 40 °C. Simultaneously, the mycelia, covered in acetone, were sonicated in an ultrasonic bath for 30 min at 40 °C. The acetone fraction was separated from the mycelia by filtration throughout a cellulose filter paper (MN 615 1/4 Ø 185 mm, Macherey Macherey-Nagel, Düren, Germany). The remaining mycelia underwent another round of sonication and extraction. Both extracts were combined, and acetone was evaporated to yield an aqueous residue in vacuo at 40 °C. This aqueous phase was extracted similarly to the supernatant. For solid cultures, the mycelia followed the same extraction process as for the mycelia obtained from liquid cultures until the evaporation of the ethyl acetate fraction. Subsequently, the ethyl acetate extract was dissolved in methanol and partitioned with an equal volume of heptane in a separatory funnel. This step was repeated with the obtained methanol phase, which was then evaporated to dryness under vacuum at 40 °C. Both methanol fractions were finally combined and dried under vacuum at 40 °C.

For the scaled-up cultivation, the fungus was grown in YM agar at 23 °C. Later, the colonies were cut into small pieces using a cork borer (1 cm × 1 cm), and eight pieces were placed into two 500 mL Erlenmeyer flasks each containing 200 mL of YM broth, which were incubated at 23 °C under shaking conditions (140 rpm) in the darkness for 7 days. Afterward, 6 mL of this seed culture was transferred to each of the 40 Erlenmeyer flasks (500 mL) containing 200 mL of Q6 1/2 broth (8 L in total) and incubated at 23 °C under shaking conditions (140 rpm) in the darkness until 3 days after glucose depletion. Consequently, the cultures followed the extraction procedure described above to afford 1845 and 558 mg of supernatant and mycelial extract, respectively.

The supernatant extract (450 mg × 4) was preseparated using reverse-phase HPLC (Büchi, Pure C-850, 2020, Switzerland) with a Gemini C18 (250 mm × 50 mm, 10 μm, Phenomenex, Torrance, CA) as the stationary phase and the following conditions as the mobile phase: solvent A: deionized water (H_2_O) + 0.1% formic acid; solvent B: acetonitrile (MeCN) + 0.1% formic acid; flow: 45 mL/min; and collected fraction volume: 15 mL. The following gradient elution was applied: holding in 5% B for 5 min, increasing from 5% B to 60% B in 60 min and then from 60% B to 100% B in 10 min, and holding in 100% B for 15 min. Five fractions (SF1–SF5) were collected, from which fraction SF5 was further purified (160 mg × 2) using reverse-phase HPLC (Büchi, Pure C-850, 2020, Switzerland) with a Gemini C18 (250 mm × 50 mm, 10 μm, Phenomenex, Torrance, CA) as the stationary phase and the following conditions as the mobile phase: solvent A: deionized water (H_2_O) + 0.1% formic acid; solvent B: acetonitrile (MeCN) + 0.1% formic acid; flow: 40 mL/min; and collected fraction volume: 15 mL. The following gradient elution was applied: holding in 5% B for 5 min, increasing from 5% B to 65% B in 15 min and then from 65% B to 100% B in 60 min, and holding in 100% B for 10 min. This resulted in the isolation of five pure compounds: **6** (3.2 mg, *t*_R_ = 15 min), **5** (1.46 mg, *t*_R_ = 32 min), **2** (4.45 mg, *t*_R_ = 43 min), **1** (4.74 mg, *t*_R_ = 45 min), and **3** (2.5 mg, *t*_R_ = 54 min).

The mycelial extract (225 mg × 2) was separated using reverse-phase HPLC (Büchi, Pure C-850, 2020, Switzerland) with a Gemini C18 (250 × 50 mm, 10 μm, Phenomenex, Torrance, CA) as the stationary phase and the following conditions as the mobile phase: solvent A, deionized water (H_2_O) + 0.1% formic acid; solvent B, acetonitrile (MeCN) + 0.1% formic acid; flow: 40 mL/min; and collected fraction volume: 15 mL. The following gradient elution was applied: holding in 5% B for 5 min, increasing from 5% B to 80% B in 15 min, and then from 80% B to 100% B in 40 min. Twelve fractions (MF1−MF12) were collected, from which MF12 corresponded to compound **4** (2.89 mg, *t*_R_ = 55 min). The fraction MF8 (25 mg) was further purified using reverse-phase HPLC (Büchi, Pure C-850, 2020, Switzerland) with an X-Bridge C18 column (250 mm × 19 mm, 5 μm, Waters, Milford, MA) as the stationary phase and the following conditions as the mobile phase: solvent A: deionized water (H_2_O) + 0.1% formic acid; solvent B: acetonitrile (MeCN) + 0.1% formic acid; flow: 20 mL/min; and collected fraction volume: 5 mL. The following gradient elution was applied: increasing from 5% B to 45% B in 5 min and then from 45% B to 70% B in 40 min and finally increasing from 70% B to 100% B in 10 min. This afforded compound **7** (1.41 mg, *t*_R_ = 29 min).

### Single-Crystal Structure Determination via 3D Electron Diffraction of Arcopilin G (**7**)

Electrons feature very strong interactions with the electrostatic potential of the atoms. Subsequently, electron diffraction allows for the performing of experiments with crystallites in the nanometer range. However, it needs to be considered that the absorption of the samples is much stronger, and the data are affected by dynamical diffraction as well as ionic scattering factors compared to X-ray diffraction. This can lead to seemingly bad R-values for refinement in the simplistic kinematic approximation.

Microcrystalline powder of **7** was spread on a standard holey carbon-coated copper TEM grid. Colorless plate-like crystallites with a few 100 nm thickness were selected for 3D ED/microED measurements. Cryotransfer, i.e., freezing of samples prior to introduction to vacuum, at −173.15 °C using a Gatan ELSA (Model 698) specimen holder was applied here. As electron diffraction requires samples to be studied under a high vacuum, the cryotransfer technique is essential for many sensitive compounds, such as solvent-containing MOFs or proteins. Next to stabilization in vacuo, other benefits are improving the resolution, reducing disorder, and reducing beam damage. Crystallites of **7** suffered from the latter one when measured at ambient temperature, resulting in no diffraction after some collected frames. The combination of cryotransfer and measurement under cryogenic conditions prolonged the lifetime of the grains.

Electron diffraction measurements for **7** were collected using the Rigaku XtaLAB Synergy-ED, equipped with a Rigaku HyPix-ED detector optimized for operation in the continuous rotation 3D-ED experimental setup.^31^ Data acquisition was performed at −173.15 °C under high vacuum with an electron wavelength of 0.0251 Å (200 kV). The instrument was operated, and the diffraction data were processed in the program CrysAlisPro.^54^ A multiscan absorption correction was performed using spherical harmonics implemented in the SCALE3 ABSPACK scaling algorithm in CrysAlisPro. The structure was solved using ShelXT^32^ and subsequently refined with kinematical approximation using ShelXL^55^ in the crystallographic program suite Olex2.^54,56^ Since we wanted to conduct dynamical refinement to determine the absolute configuration of **7**, a single dataset with as much completeness as possible was collected (grain 1, 60.3%) and thus used for refinement, instead of collecting several datasets followed by data merging for full data completeness. For initial kinematical refinement, non-hydrogen atoms were assigned isotropic displacement parameters. The hydrogen atoms bonded to the oxygen atoms were located from Fourier difference maps. Other hydrogen atoms were placed in idealized positions and included as riding. Isotropic displacement parameters for all H atoms were constrained to multiples of the equivalent displacement parameters of their parent atoms with U_iso_(H) = 1.2 U_eq_(parent atom). The experimental and refinement details are given below. CSD 2311560 contains the supplementary crystallographic data for this publication. These data can be obtained free of charge via www.ccdc.cam.ac.uk/data_request/cif or by emailing data_request@ccdc.cam.ac.uk or by contacting The Cambridge Crystallographic Data Centre, 12 Union Road, Cambridge CB2 1EZ, U.K.; fax: + 44 1223 336033.

### Spectral Data

Optical rotations were recorded employing an MCP 150 circular polarimeter (Anton Paar, Seelze, Germany) at 20 °C. UV/vis spectra were recorded with a UV-2450 spectrophotometer (Shimadzu, Kyoto, Japan). Spectral data were measured in MeOH (Uvasol, Merck, Darmstadt, Germany) for all compounds. All compounds used in this study for the *in vitro* experiments were >95% pure as confirmed by NMR analysis, which are included in the Supporting Information of the manuscript. The respective 1D and 2D NMR spectra were recorded with an Avance III 700 spectrometer with a 5 mm TCI cryoprobe (^1^H NMR: 700 MHz, ^13^C: 175 MHz, Bruker, Billerica, MA) and an Avance III 500 spectrometer (^1^H NMR: 500 MHz, ^13^C: 125 MHz, Bruker, Billerica, MA). The chemical shifts δ were referenced to the solvent DMSO-*d*_6_ (^1^H, δ = 2.50; ^13^C, δ = 39.51).

#### Arcopilin A (**1**)

Brown to orange oil; [α]_d_^20^ −12 (c 0.001, MeOH); UV (MeOH) λmax (log ε) 203 (0.175), 237.5 (0.158), 313 (0.275); ESI-MS: *m*/*z* 368.20 [M – H]^−^, 370.20 [M + H]^+^, and 392.20 [M + Na]^+^; HRESI-MS: *m*/*z* 370.2025 [M + H]^+^ (calculated for C_22_H_28_NO_4_^+^: 370.2013 Da).

#### Arcopilin B (**2**)

Brown to orange oil; [α]_d_^20^ −8 (c 0.001, MeOH); UV (MeOH) λmax (log ε) 203 (0.178), 238 (0.153), 313 (0.265); ESI-MS: *m*/*z* 370.20 [M – H]^−^, 372.20 [M + H]^+^, and 394.20 [M + Na]^+^; HRESI-MS: *m*/*z* 372.21512 [M + H]^+^ (calculated for C_22_H_30_NO_4_^+^: 372.2169 Da).

#### Arcopilin C (**3**)

Brown to orange oil; [α]_d_^20^ −3.5 (c 0.001, MeOH); UV (MeOH) λmax (log ε) 201.5 (0.688), 230.5 (0.426), 290 (0.727); ESI-MS: *m*/*z* 398.06 [M – H]^−^, 400.25 [M + H]^+^, and 422.24 [M + Na]^+^; HRESI-MS: *m*/*z* 400.48531 [M + H]^+^ (calculated for C_23_H_30_NO_5_^+^: 400.4868 Da).

#### Arcopilin D (**4**)

Brown to orange oil; [α]_d_^20^ −20 (c 0.001, MeOH); UV (MeOH) λmax (log  ε) 201 (0.524), 231.5 (0.340), 313.5 (0.650); ESI-MS: *m*/*z* 354.23 [M – H]^−^, 356.26 [M + H]^+^, and 378.22 [M + Na]^+^; HRESI-MS: *m*/*z* 356.2237 [M + H]^+^ (calculated for C_22_H_30_NO_3_^+^: 356.2220 Da).

#### Arcopilin E (**5**)

Brown to orange oil; [α]_d_^20^ −3.4 (c 0.001, MeOH); UV (MeOH) λmax (log ε) 203 (0.297), 314 (0.357); ESI-MS: *m*/*z* 400.05 [M – H]^−^, 402.22 [M + H]^+^, and 424.21 [M + Na]^+^; HRESI-MS: *m*/*z* 402.1921 [M + H]^+^ (calculated for C_22_H_28_NO_6_^+^: 402.1911 Da).

#### Arcopilin F (**6**)

Brown to orange oil; [α]_d_^20^ −1.8 (c 0.001, MeOH); UV (MeOH) λmax (log ε) 201 (0.813), 232.5 (0.503), 312 (0.840); ESI-MS: *m*/*z* 416.05 [M – H]^−^, 418.22 [M + H]^+^, and 440.20 [M + Na]^+^; HRESI-MS: *m*/*z* 418.1861 [M + H]^+^ (calculated for C_22_H_28_NO_7_^+^: 417.1860 Da).

#### Arcopilin G (**7**)

Orange to white powder; [α]_d_^20^ −15 (c 0.001, MeOH); UV (MeOH) λmax (log ε) 207.5 (0.733), 241 (0.702), 304.5 (0.229); ESI-MS: *m*/*z* 370.21 [M – H]^−^, 372.23 [M + H]^+^, and 394.20 [M + Na]^+^; HRESI-MS: *m*/*z* 372.2156 [M + H]^+^ (calculated for C_22_H_30_NO_4_^+^: 372.2169 Da).

#### Crystallographic Data (**7**)

Grain 1 only: CSD 2311560, colorless plate, C_22_H_29_NO_4_, *M*_r_ = 371.46 gmol^–1^, monoclinic, space group *I*2 (No. 5), *a* = 16.7(3) Å, *b* = 7.07(18) Å, *c* = 16.83(14) Å, α = 90°, β = 90.47(10)°, γ = 90°, *V* = 1989(62) Å3, *Z* = 4, *Z′* = 1, T = −173.15 °C, *m*(transmission electron microscope) = 0.000, 3761 total reflections, 1070 with I_0_ > 2σ(I_0_), resolution = 0.837 Å, completeness = 60.3%, redundancy = 3.2, *R*_int_ = 0.1079, *R*_pim_ = 0.082, *CC*1/2 = 0.990, 2127 data, 101 parameters, 15 restraints, GOF = 1.749, R_1_ = 0.2075 and w*R*_2_ = 0.4588 [I_0_ > 2σ(I_0_)], R_1_ = 0.2762 and w*R*_2_ = 0.4988 (all reflections), 0.152 < dΔρ < −0.121. Merged grain 1 and 2: C_22_H_29_NO_4_, *M*_r_ = 371.46 gmol^–1^, monoclinic, space group *I*2 (No. 5), *a* = 16.7(3) Å, *b* = 7.07(18) Å, *c* = 16.83(14) Å, α = 90°, β = 90.47(10)°, γ = 90°, *V* = 1989(62) Å3, *Z* = 4, *Z′* = 1, T = −173.15 °C, m(transmission electron microscope) = 0.000, 8321 total reflections, 1598 with I_0_ > 2σ(I_0_), resolution = 0.837 Å, completeness = 99.9%, redundancy = 4.3, R_int_ = 0.1794, R_pim_ = 0.101, *CC*1/2 = 0.989, 3557 data, 236 parameters, 5 restraints, GOF = 1.605, R_1_ = 0.2171 and w*R*_2_ = 0.4702 [I_0_ > 2σ(I_0_)], R_1_ = 0.2910 and w*R*_2_ = 0.5027 (all reflections), 0.226 < dΔρ < −0.188.

### Derivatization of Arcopilin B (**2**) with MTPA

Arcopilin B (**2**) was dissolved in pyridine-*d*_5_ (50 μL) and transferred into a 250 μL glass vial, and (*R*)-(−)-α-methoxy-α-(trifluoromethyl) phenylacetyl chloride (4 μL) was added. The mixture was incubated for 2 h at room temperature before being transferred to an NMR tube (600 μL) and diluted to a final volume of 350 μL for the measurement of ^1^H, TOCSY, and HSQC NMR spectra. ^1^H NMR data (700 MHz, pyridine-*d*_5_): similar to **2**, but δ_H_ 5.17 (m, 13–H), 1.87 (m, 12–H), 1.17 (d, J = 6.3 Hz, 14–H_3_) and 0.89 (d, J = 6.9 Hz, 17–H_3_).

The (*R*)-MTPA ester derivative was obtained analogously with (*S*)-(+)-α-methoxy-α-(trifluoromethyl) phenylacetyl chloride (4 μL). ^1^H NMR data (700 MHz, pyridine-*d*5): similar to **2**, but δ_H_ 5.15 (m, 13–H), 1.83 (m, 12–H), 1.26 (d, J = 6.3 Hz, 14–H_3_) and 0.81 (d, J = 6.9 Hz, 17–H_3_).

### Antimicrobial and Cytotoxic Assays

The antimicrobial and cytotoxic assays were performed according to the methods reported previously.^57^

### Biofilm Assays

Cultures of *S. aureus* DSM 1104 were prepared by inoculating 1 mL aliquots from a frozen stock (−20 °C) into 25 mL of CASO medium and incubating them overnight at 37 °C with shaking at 130 rpm.

### Preformed Biofilms

The crystal violet assay was performed according to a previously reported procedure.^47^ Arcopilins A–G were tested in serial dilutions (250–2 μg/mL), with methanol and microporenic acid A (MAA) as negative and positive controls, respectively. Statistical differences between samples and the controls were determined using a two-tailed Student’s *t* test, with statistical significance defined as *p* < 0.01. Statistical analysis was carried out using GraphPad Prism 9 (GraphPad Software, San Diego, CA).^58^

### XTT Assay

The seed culture of *S. aureus* DSM 1104 was prepared as previously described, and its OD600 was adjusted to match the turbidity of a 0.001 McFarland standard. Next, 150 μL of this bacterial solution in CASO with 4% glucose broth was incubated in 96-well tissue plates (TPP tissue culture ref no. 92196, Switzerland) for 24 h at 150 rpm. After incubation, the supernatant was discarded and 150 μL of the fresh media was added to the wells, along with serially diluted arcopilin A (31.3–0.5 μg/mL). The plate was further incubated for 24 h. Afterward, XTT (Cell profile XTT kit, Roche, Switzerland) was prepared in phosphate-buffered saline (PBS) at a final concentration of 0.3 mg/mL. The plate was washed three times with PBS buffer, and then, 150 μL of the prepared XTT solution was added to each well. Plates were further incubated for an additional 4 h at 37 °C while shaking (150 rpm), and absorbance was measured at 490 nm using a plate reader (Synergy 2, BioTek, Santa Clara). Methanol (2.5%) was used as the solvent control. Error bars indicate the standard deviation (SD) of duplicate with two repeats.

### Growth Curve of *S. aureus*

The seed culture of *S. aureus* DSM 1104 was adjusted to match the turbidity of a 0.1 McFarland standard and then cultured at 37 °C and 150 rpm in CASO with 4% glucose broth. Subsequently, it was added together with arcopilin A to a 96-well nontissue microtiter plate (TPP nontissue culture refno 92197, Switzerland) and serially diluted (31.3–2 μg/mL).^58^ Absorbance was measured using a plate reader (Synergy 2, BioTek, Santa Clara) at 530 nm every 90 min. Methanol (2.5%) was used as the solvent control. Error bars indicate the SD of duplicate with two repeats.

### Fractional Inhibitory Concentration Indices (FICIs)

The interaction between arcopilin A, vancomycin, and gentamicin against *S. aureus* DSM 1104 was evaluated using a checkerboard broth dilution method to determine the fractional inhibitory concentration indices (FICIs), calculated as FIC = MIC of drug A in combination/MIC of drug A alone + MIC of drug B in combination/MIC of drug B alone.^59^ The FICIs were interpreted as synergistic (FICI ≤ 0.5). For this assay, a seed culture was prepared as previously described to inoculate fresh CASO with 4% glucose broth to match the turbidity of a 0.1 McFarland standard suspension. Then, 100 μL of bacterial suspension was distributed in 96-well nontissue microtiter plates (TPP nontissue culture ref no. 92197, Switzerland). Arcopilin A (7.8–2 μg/mL) and antibiotics (vancomycin and gentamicin: 31.3–0.016 μg/mL) were added in increasing concentrations in columns and rows, respectively. The experiments were conducted in duplicate.

### Synergistic Effects on Preformed Biofilms

The seed culture of *S. aureus* DSM 1104 was prepared as previously described, and the OD600 was adjusted to match the turbidity of a 0.001 McFarland standard. Then, 150 μL of bacterial solution in CASO with 4% glucose broth was incubated in 96-well tissue plates (TPP tissue culture ref no. 92196, Switzerland) for 24 h at 150 rpm. Afterward, the supernatant was discarded, and 150 μL of the fresh media was added to the wells, together with serially diluted arcopilin A (15.6–3.9 μg/mL), vancomycin (15.6–2 μg/mL, Sigma Aldrich), and gentamicin (7.8–2 μg/mL, Sigma Aldrich) as well as their combinations. Methanol (2.5%) was used as the solvent control. The plates were incubated for a further 24 h at 37 °C. Colony-forming unit (CFU) count analysis of arcopilin A, antibiotics (vancomycin and gentamicin), and their combinations was performed as previously described.^58^ Cells were suspended in the well 50 times. Dilution series in 1 to 10 steps (20 μL in 200 μL) were prepared down to a final dilution level of 10^–6^, and 100 μL of this last dilution was platted on LB agar plates using 3 mm small glass beads (5 to 10, Omnilab, Germany) to homogeneously spread the liquid. Individual colonies on agar plates were counted after incubation at 30 °C for 24 and 48 h.^60^ Afterward, CFUs were calculated by considering the dilution factors. Error bars indicate the SD of duplicate with two repeats.

## Supporting Information Available

HPLC-UV/Vis chromatograms (210 nm) of the crude extracts obtained after the cultivation of *A. navicularis* CCF3252 in three different liquid media (YM 6.3, ZM 1/2, Q6 1/2) and one solid medium (BRFT); data collection parameter overview; transmission electron microscopy images and exemplary diffraction patterns; ^1^H NMR; HSQC NMR; J-HMBC NMR; and ^13^C NMR spectra; and effects of Acp A on the growth of *S. aureus* DSM 1104 planktonic cells (PDF)
Compound SMILES (CSV)

## Abbreviations

3D ED: 3D electron diffraction
15-Ht: 15-hydroxytenellin
Acp: arcopilin
BRFT: rice solid medium
CCF: Culture Collection of Fungi in Prague
CFU: colony-forming unit
FICI: fractional inhibitory concentration Index
GM: gentamicin
HPLC: high-performance liquid chromatography
HR-ESI-MS: high-resolution electrospray ionization mass spectrometry
IC_50_: half-maximum inhibitory concentration
MIC: minimum inhibitory concentration
MRSA: methicillin-resistant *S. aureus*
NMR: nuclear magnetic resonance
PKS-NRPS: polyketide synthase–nonribosomal peptide synthetase
Vac: vancomycin
YM: yeast malt
