# Supplementary material for: Arcopilins: A New Family of Staphylococcus aureus Biofilm Disruptors from the Soil Fungus Arcopilus navicularis
Source: J Med Chem. 2024 Aug 14;67(17):15029–40. doi: 10.1021/acs.jmedchem.4c00585 (PMC11403616; doi:10.1021/acs.jmedchem.4c00585)
Supplement: Supplementary file 1 — jm4c00585_si_001.pdf [file jm4c00585_si_001.pdf]

Supplementary Information

Arcopilins, a new family of *Staphylococcus aureus* biofilm disruptors from the soil fungus *Arcopilus navicularis*

Esteban Charria-Girón[a,b‡], Haoxuan Zeng[a,b‡], Tatiana Gorelik[c,d], Khai-Nghi Truong[e], Alexandra
Pahl[a,b], Hedda Schrey[a,b], Frank Surup[a,b,*], Yasmina Marin-Felix[a,b,*]

[a]Department Microbial Drugs, Helmholtz Centre for Infection Research (HZI), German Centre for Infection Research (DZIF), Partner Site Hannover-Braunschweig, Inhoffenstrasse 7, 38124 Braunschweig, Germany.

[b]Institute of Microbiology, Technische Universität Braunschweig, Spielmannstraße 7, 38106 Braunschweig, Germany.

[c]Department Structure and Function of Proteins, Helmholtz Centre for Infection Research (HZI), Inhoffenstrasse 7, 38124 Braunschweig, Germany.

[d]Department Microbial Natural Products, Helmholtz-Institute for Pharmaceutical Research Saarland (HIPS), Campus E8.1, 66123 Saarbrücken, Germany.

[e]Rigaku Europe SE, Hugenottenallee 167, 63263 Neu-Isenburg, Germany

Correspondence: frank.surup@helmholtz-hzi.de (Frank Surup); yasmina.marinfelix@helmholtz-hzi.de (Yasmina Marin-Felix).

Table of Contents

Figure S1. 3
Table S1. 3
Table S2. 3
Figure S2. 4
Figure S3. 4
Figure S4. 5
Figure S5. 6
Figure S6. 7
Figure S7. 8
Figure S8. 9
Figure S9 10
Figure S10. 11
Figure S11. 12
Figure S12. 13
Figure S13. 14

Figure S14. 15
Figure S15. 16
Figure S16. 17
Figure S17. 18
Figure S18. 19
Figure S19 20
Figure S20. 21
Figure S21. 22
Figure S22. 23
Figure S23. 24
Figure S24. 25
Figure S25. 26
Figure S26 27
Figure S27. 28
Figure S28. 29
Figure S29. 30
Figure S30. 31
Figure S31. 32
Figure S32. 33
Figure S33. 34
Figure S34. 35
Figure S35. 36
Figure S36. 37
Figure S37. 38
Figure S38. 39
Figure S39. 40
Figure S40. 41
Figure S41. 42
Figure S42. 43
Figure S43. 44
Figure S44. 45
Figure S45. 46
Figure S46. 47
Figure S47. 47
References 47

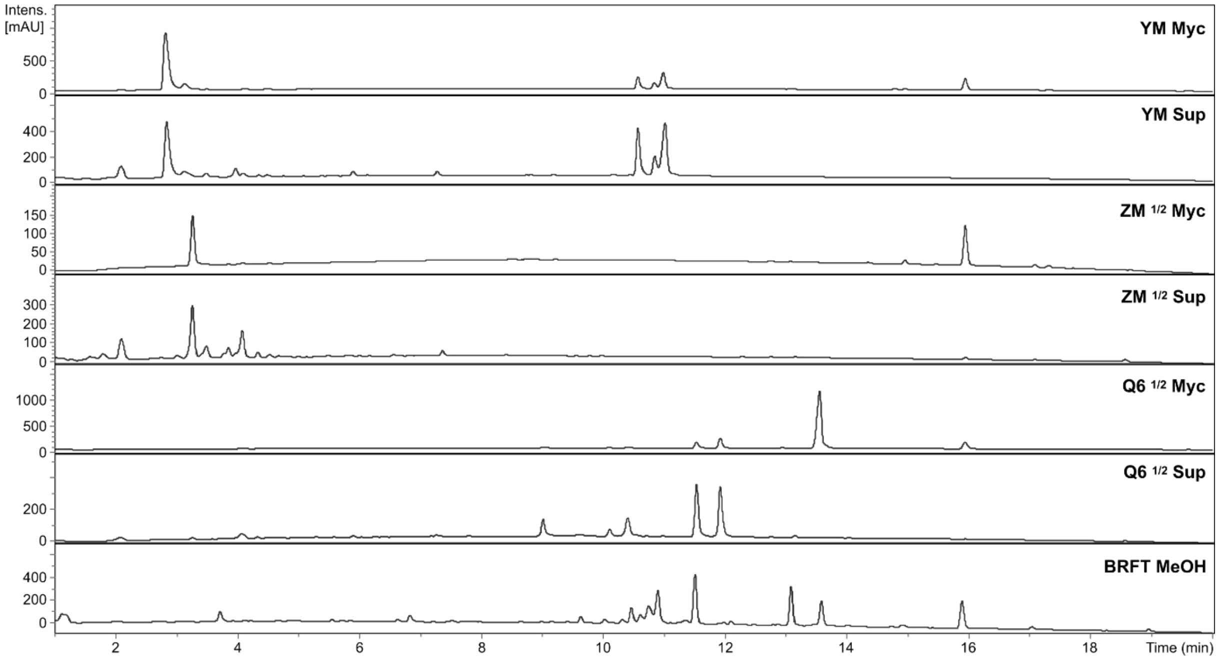

Figure S1. HPLC-UV/Vis chromatograms (210 nm) of the crude extracts obtained after the cultivation of *A. navicularis* CCF 3252 in three different liquid media (YM 6.3, ZM ½, Q6 ½) and one solid medium (BRFT). For liquid cultures, the crude extracts obtained from the supernatant and mycelia are represented by Sup and Myc respectively.

Table S1. Data collection parameter overview.

Compound	Scan range [°]	Scan width [°]	Number of frames	Exposure time/frame [s]	Total exposure time [s]	Dose rate [$e^-/(Å^2 \cdot s)$]	Dose [$e^-/(Å^2)$]
**7** (grain 1)	-50 to +50	0.25	400	0.25	100	1.08E-03	0.11
***** (grain 2)	-60 to +60	0.25	480	0.25	120	1.14E-03	0.14

* Second grain was used for data merging. A completeness of 99.9% up to a resolution of 0.837 Å was achieved after data merging.

Table S2. Comparison of both enantiomers. The absolute configuration of **7** was determined by dynamical refinement[12] using the program suite Jana2020.[13] A background noise-adjusted z-score of 4.92 was computed in favor for the *7S,8S,10S,11S*-enantiomer.[14]

Enantiomer	Refinement	R_{obs}	wR_{obs}	R_{all}	wR_{all}	GOF_{obs}	GOF_{all}
7R,8R,10R,11R	dynamical	19.96	40.70	25.12	46.75	2.08	1.41
7S,8S,10S,11S	dynamical	16.31	39.01	23.93	44.93	2.00	1.37

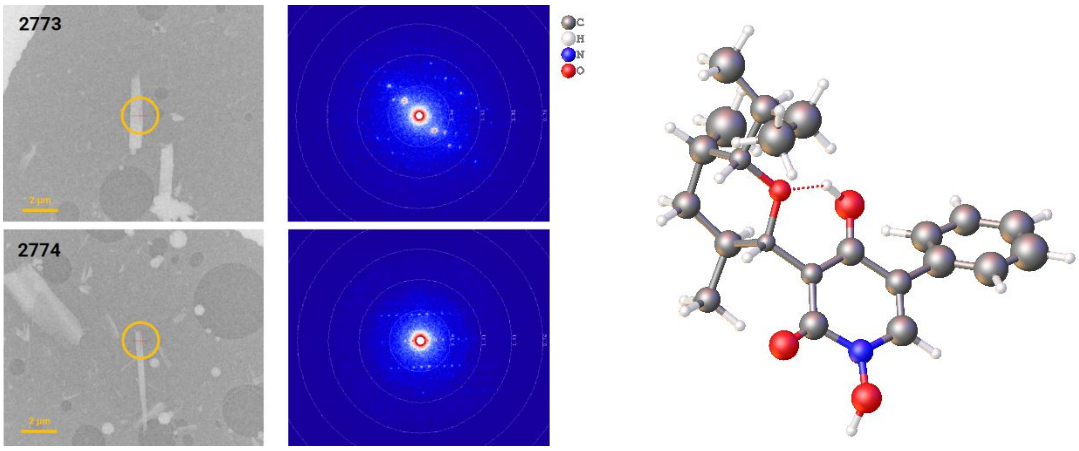

Figure S2. (Left) Transmission electron microscope images and exemplary diffraction patterns. Spherical crystallites are ice. (Right) the asymmetric unit of 7 after kinematical refinement. The red dotted line represents O–H···O hydrogen bonding.

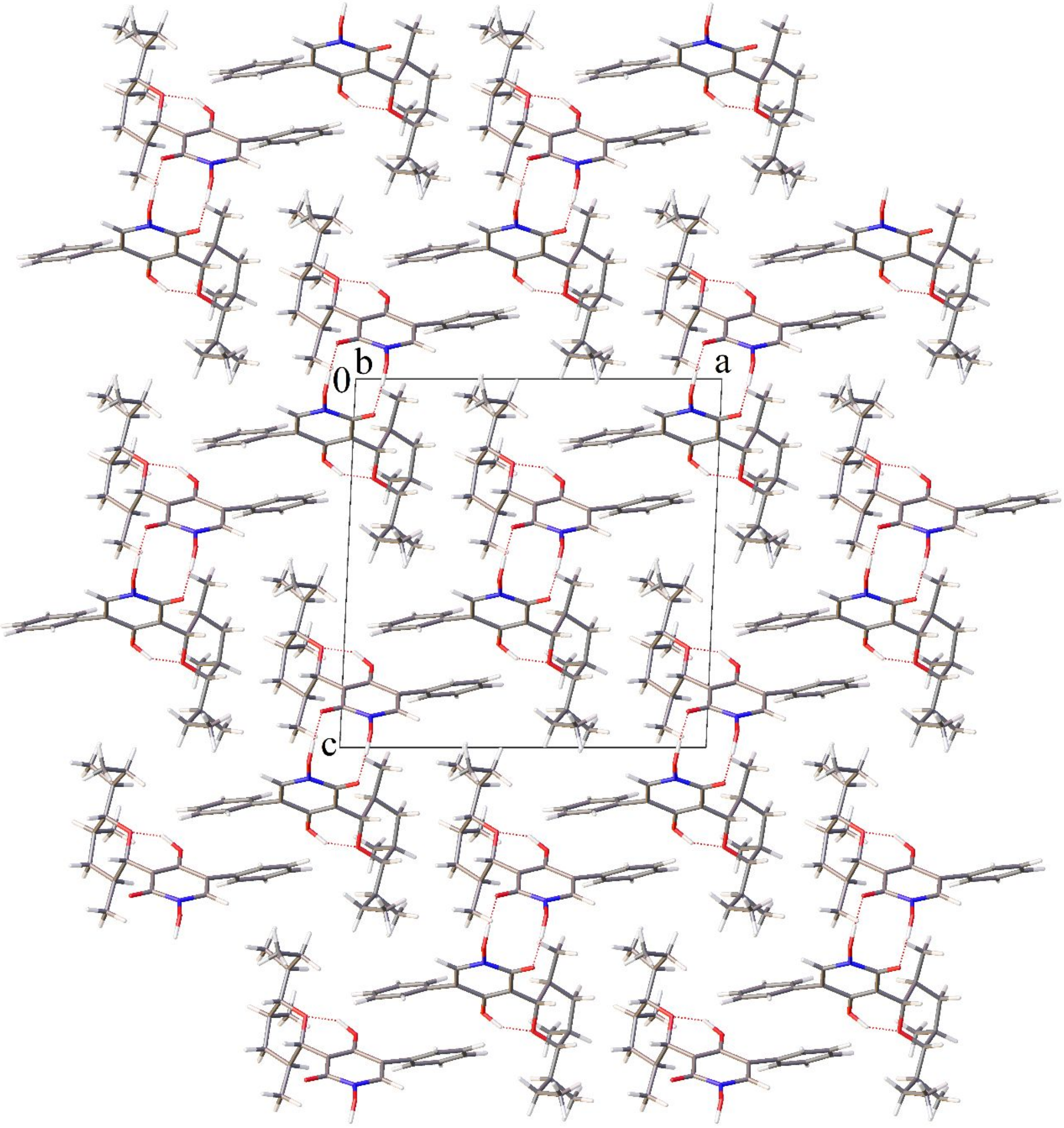

Figure S3. Crystal packing viewing along *b* direction shows dimer formation via hydrogen bonds.

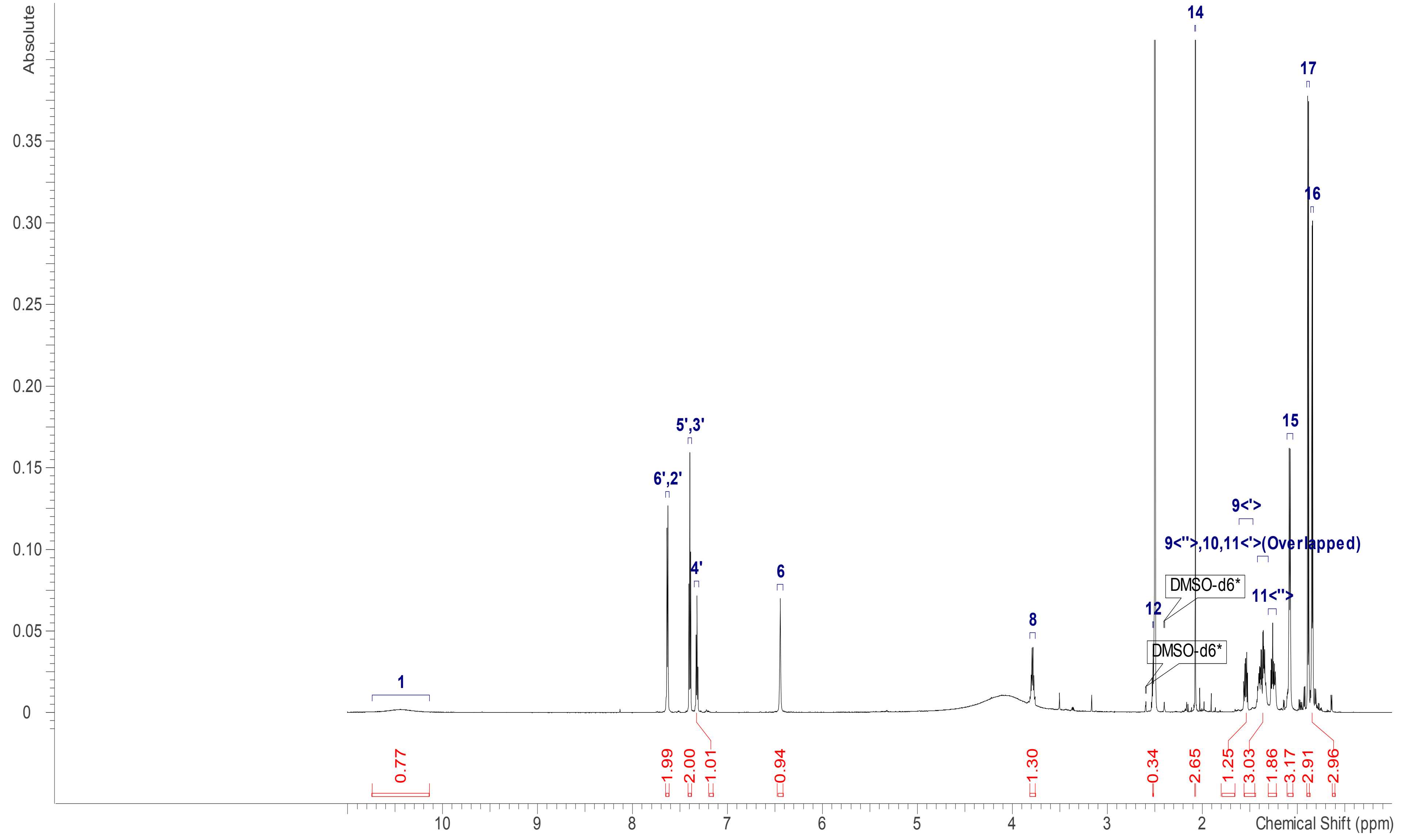

Figure S4. ^{1}H NMR spectrum of **1** (700 MHz, DMSO-d_6).

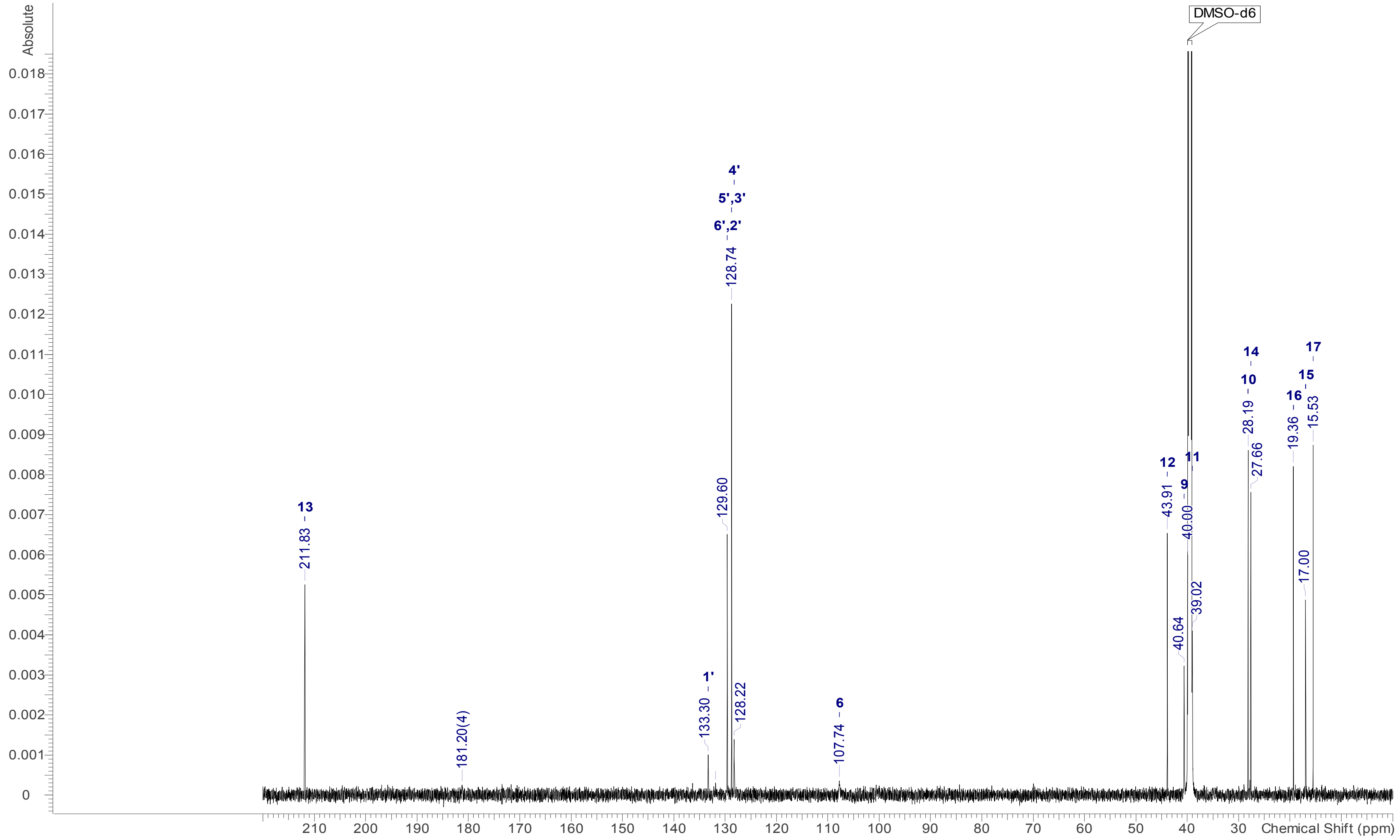

Figure S5. ^{13}C NMR spectrum of **1** (175 MHz, DMSO-d_6).

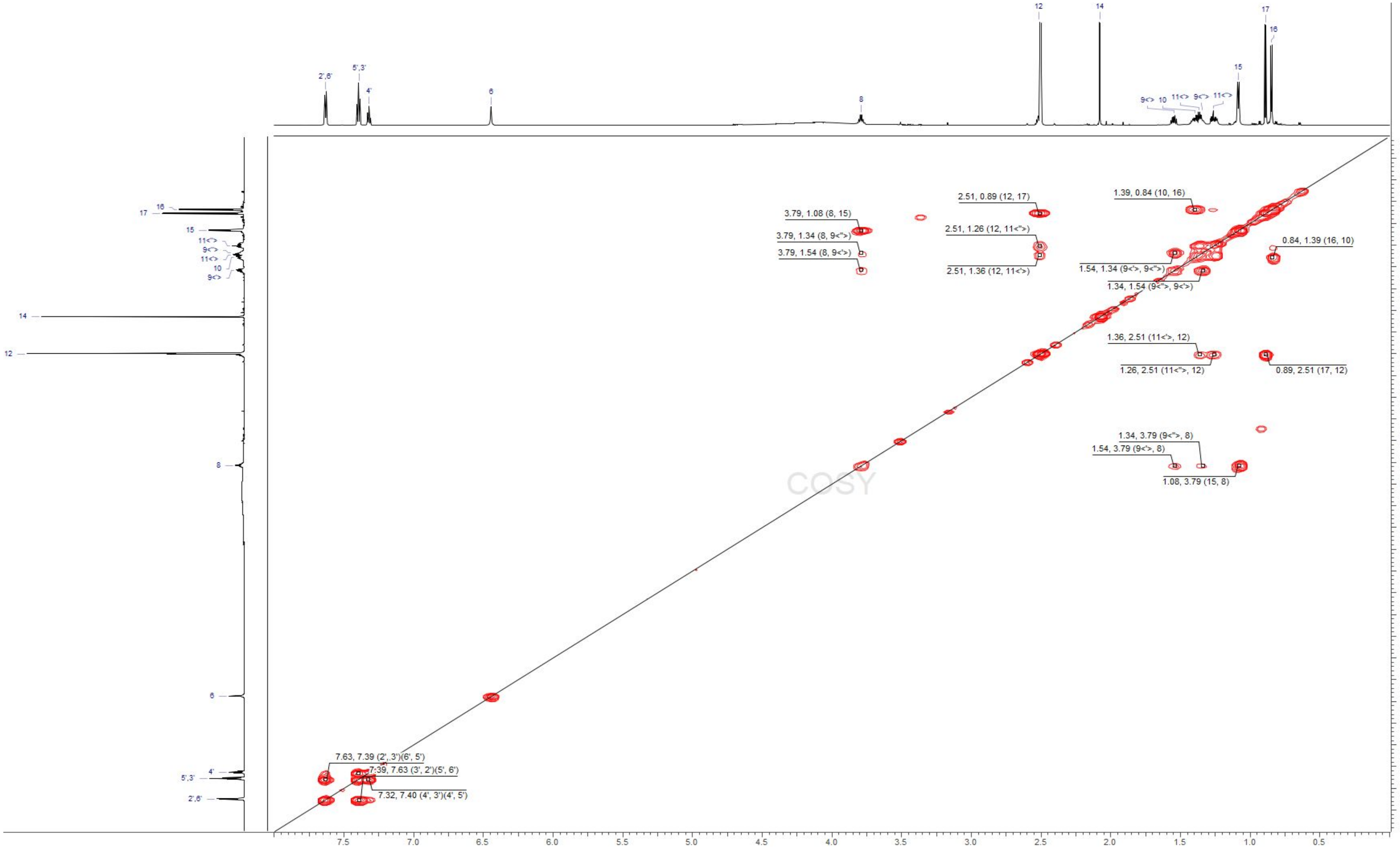

Figure S6. COSY NMR spectrum of **1** (700 MHz, DMSO-d_6).

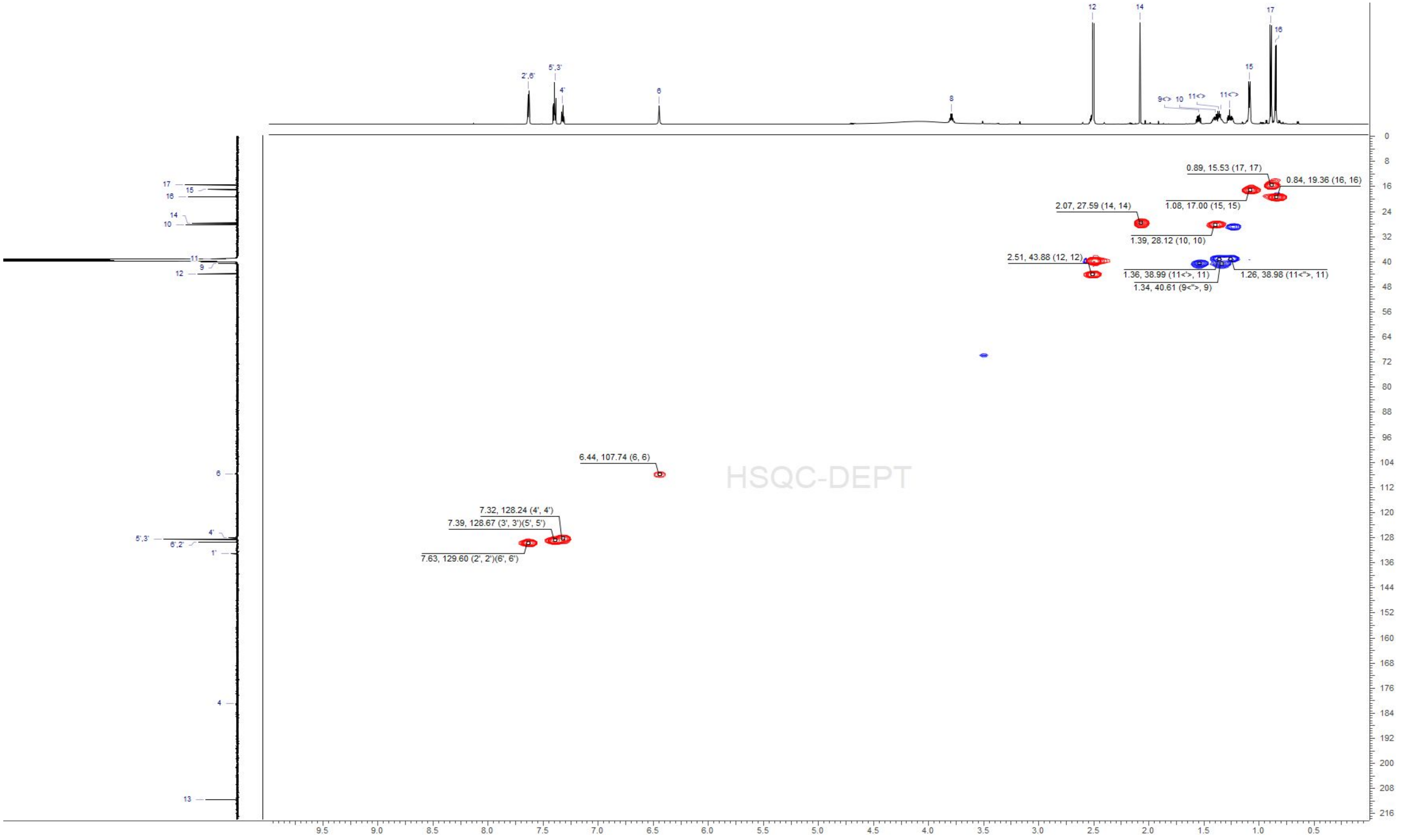

Figure S7. HSQC NMR spectrum of **1** (700 MHz, DMSO-d_6).

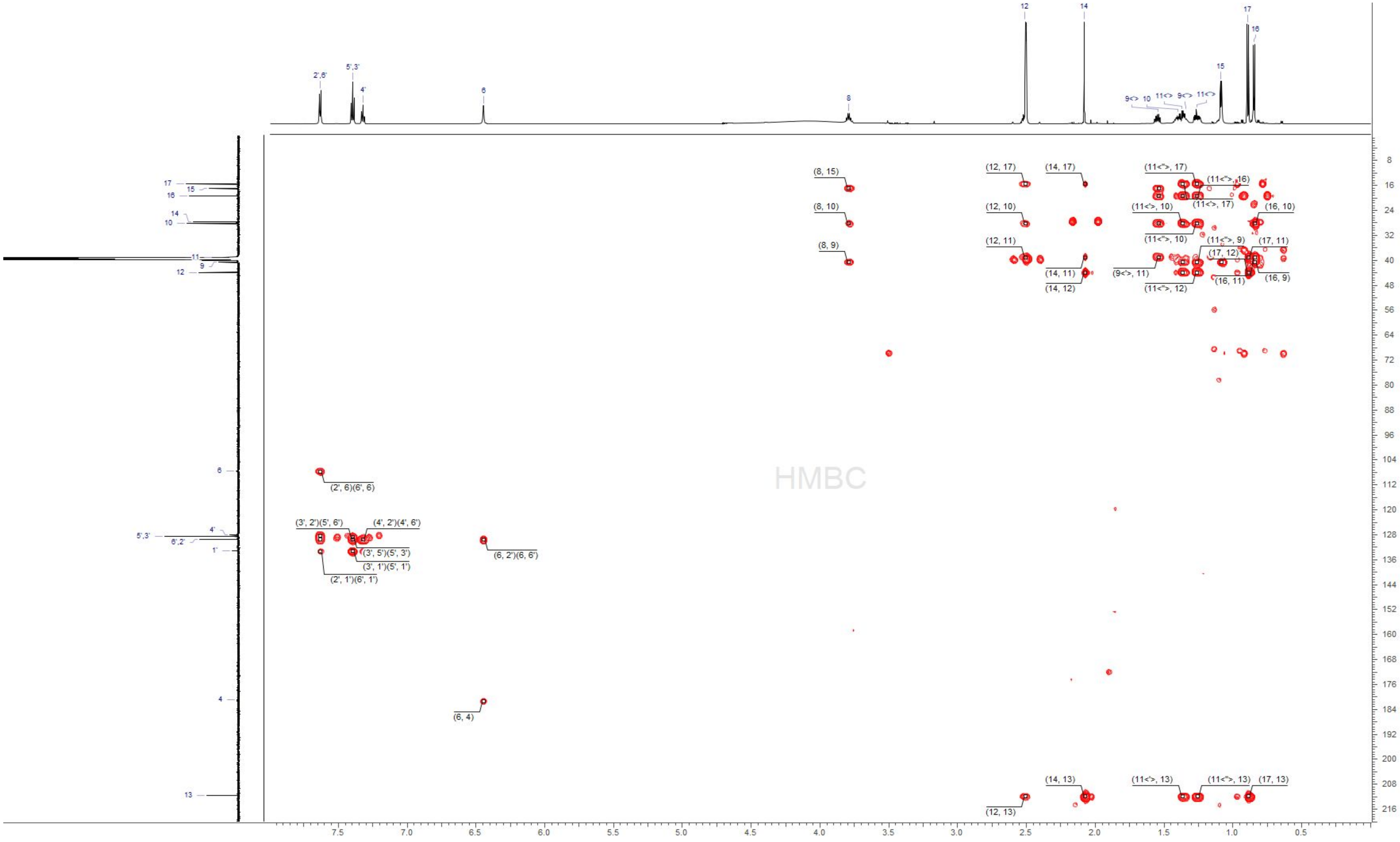

Figure S8. HMBC NMR spectrum of **1** (700 MHz, DMSO-d_6).

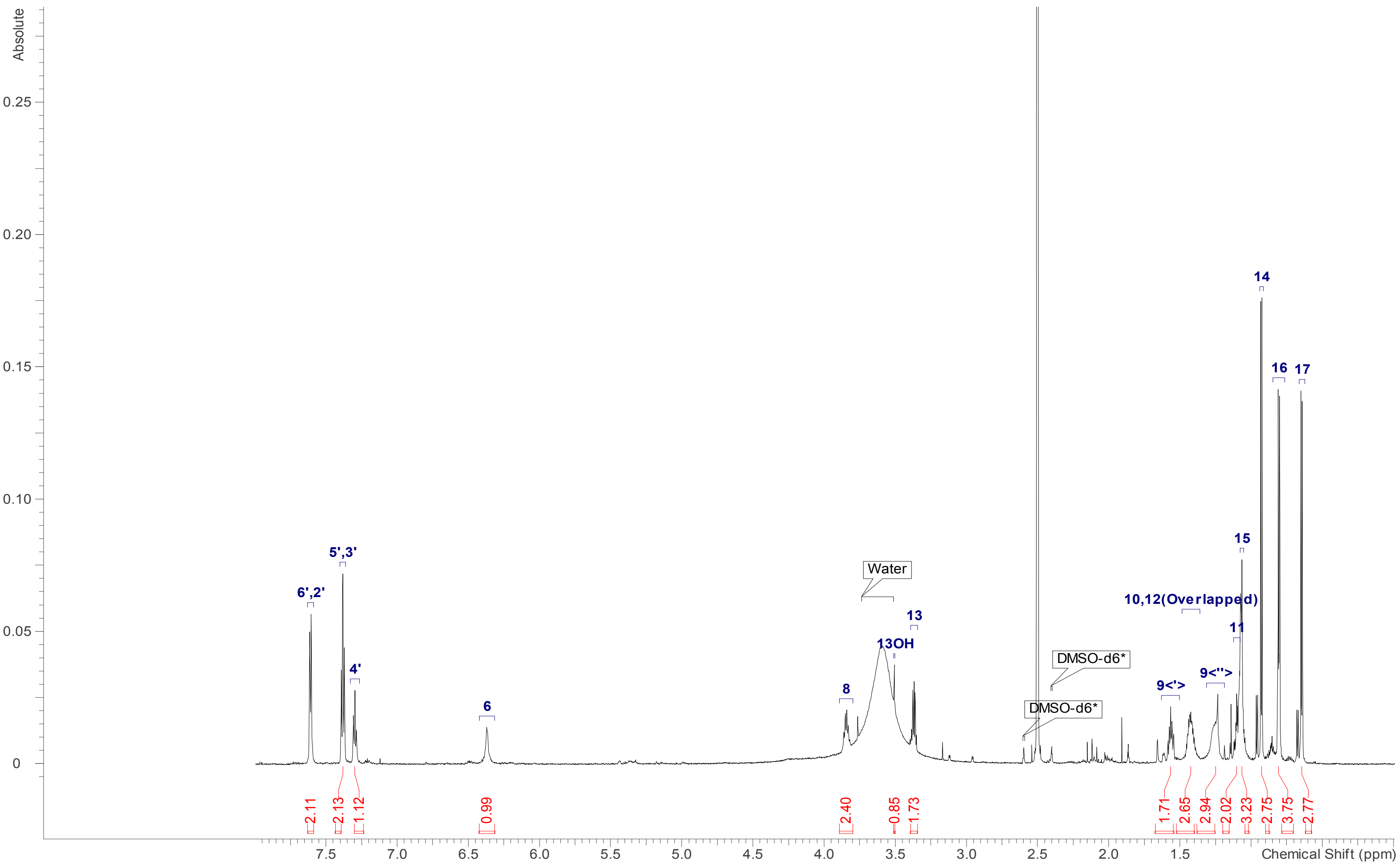

Figure S9. ^{1}H NMR spectrum of **2** (700 MHz, DMSO-d_6).

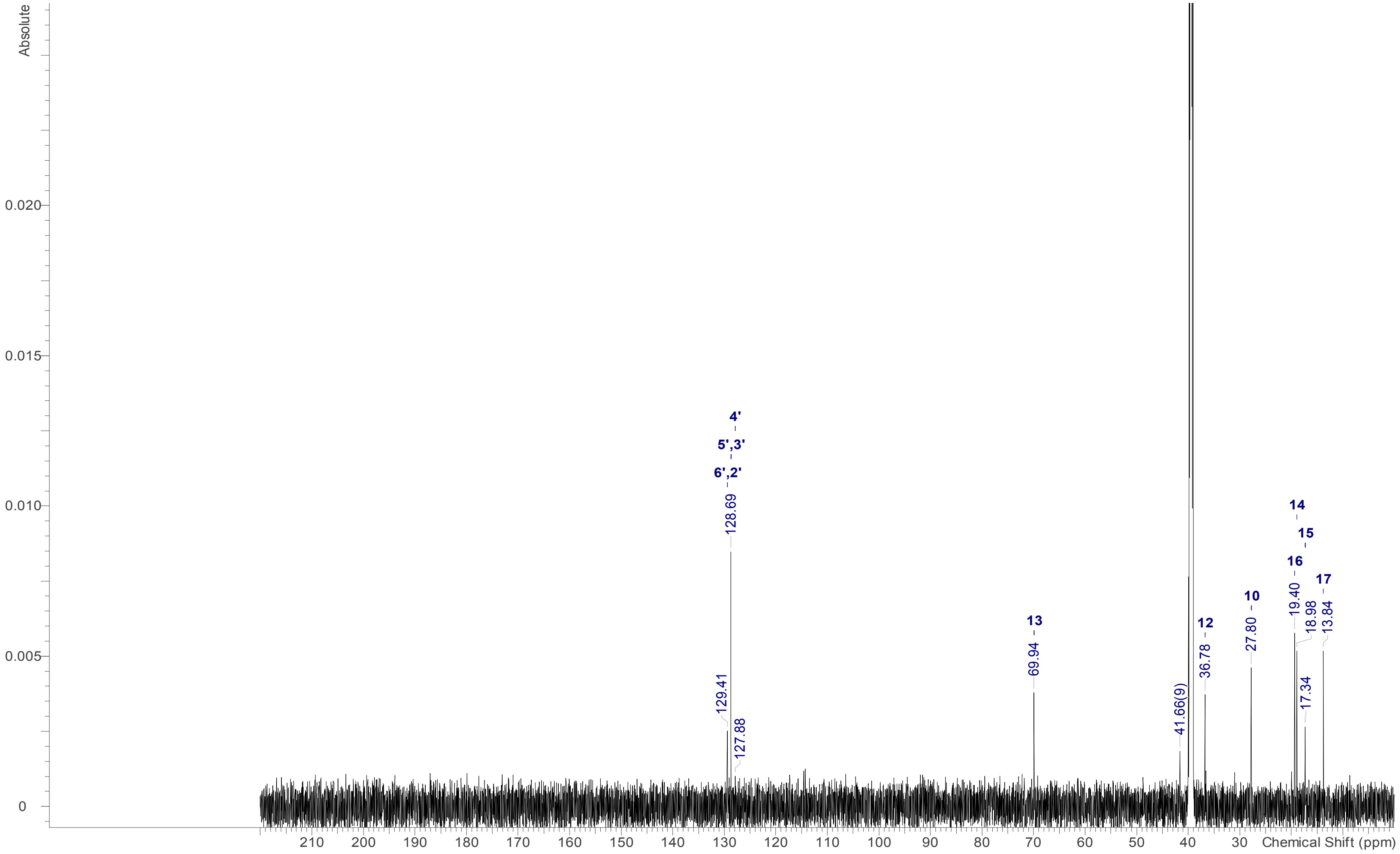

Figure S10. ^{13}C NMR spectrum of **2** (175 MHz, DMSO-d_6).

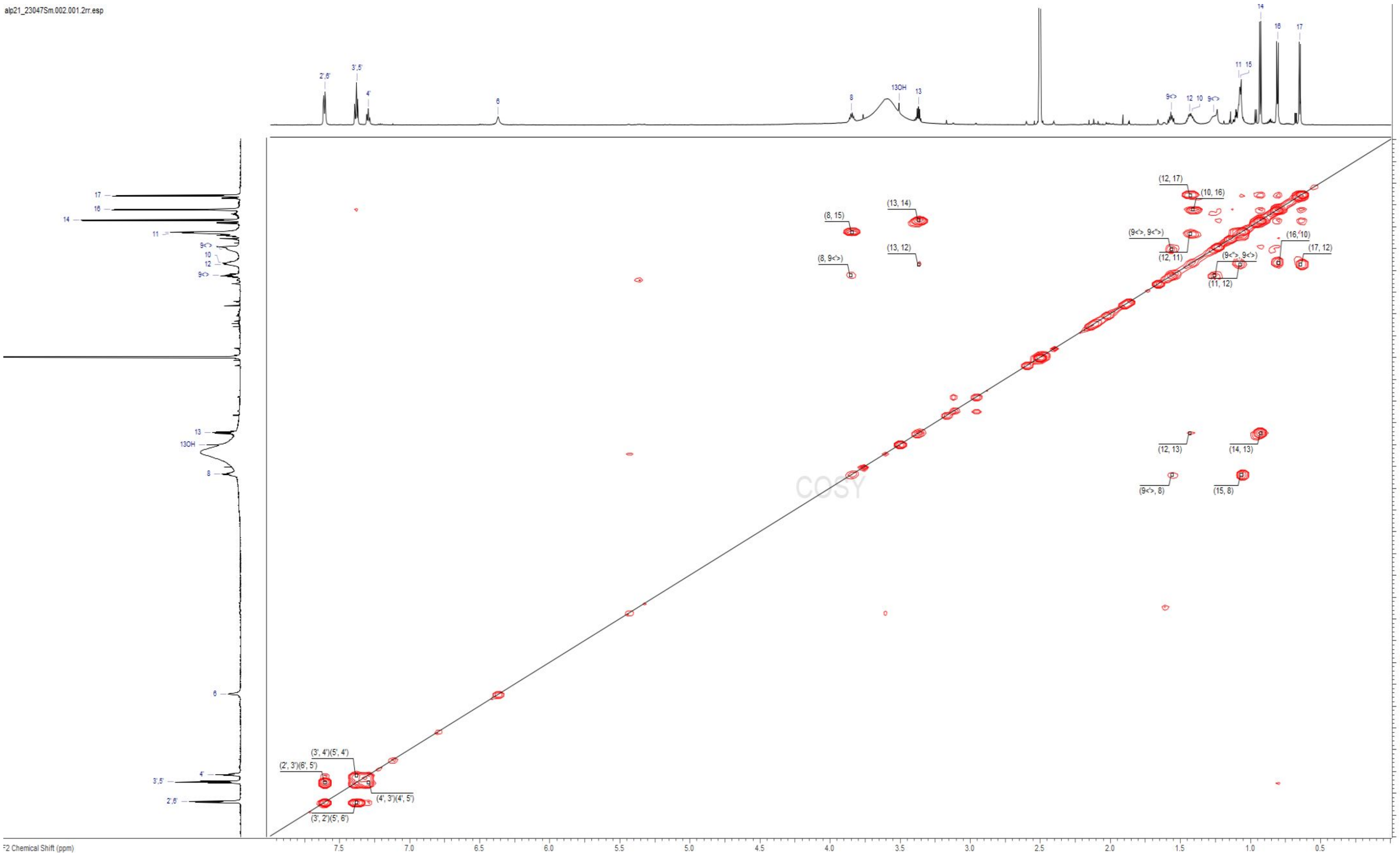

Figure S11. COSY NMR spectrum of **2** (700 MHz, DMSO-d_6).

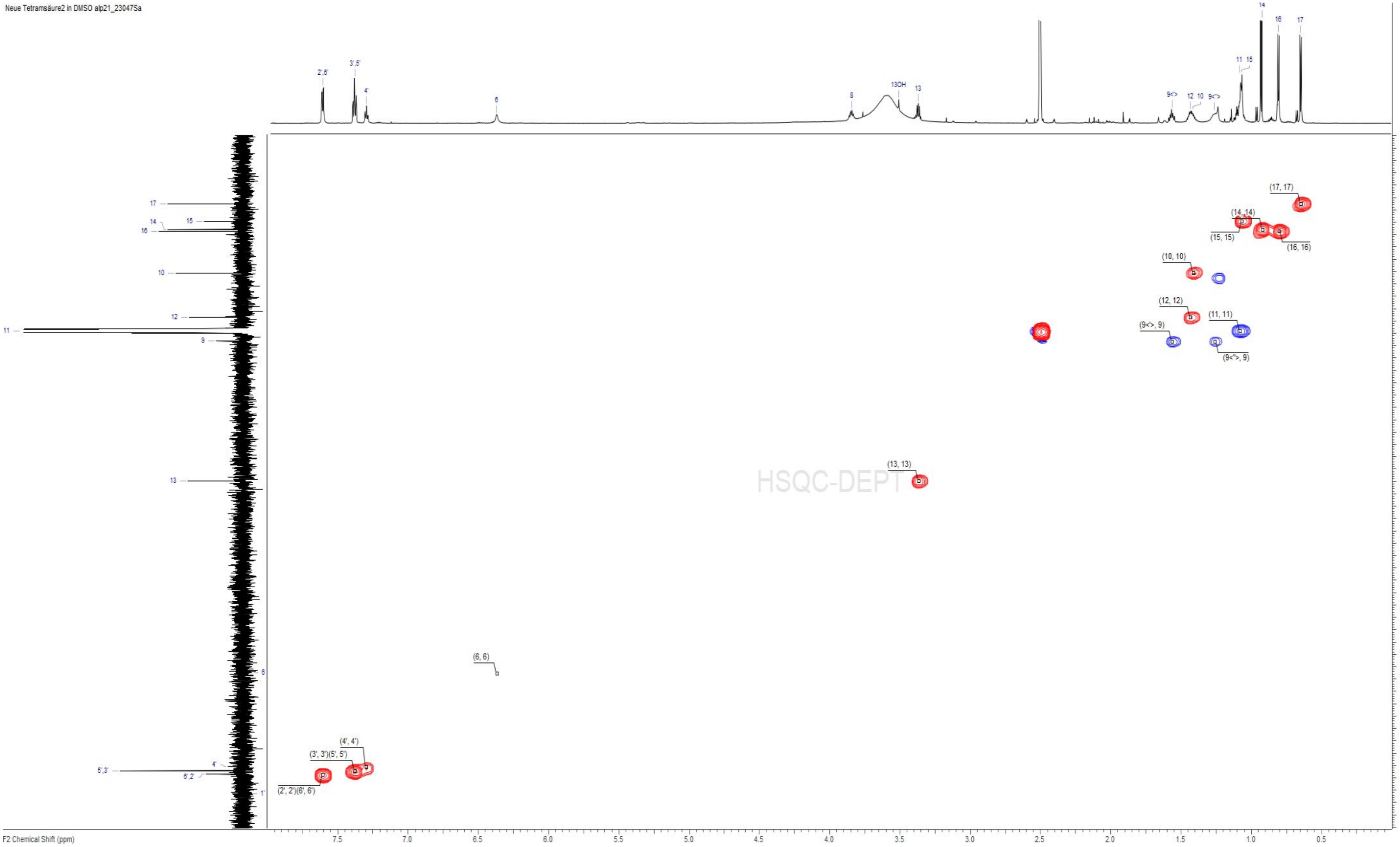

Figure S12. HSQC NMR spectrum of **2** (700 MHz, DMSO-d_6).

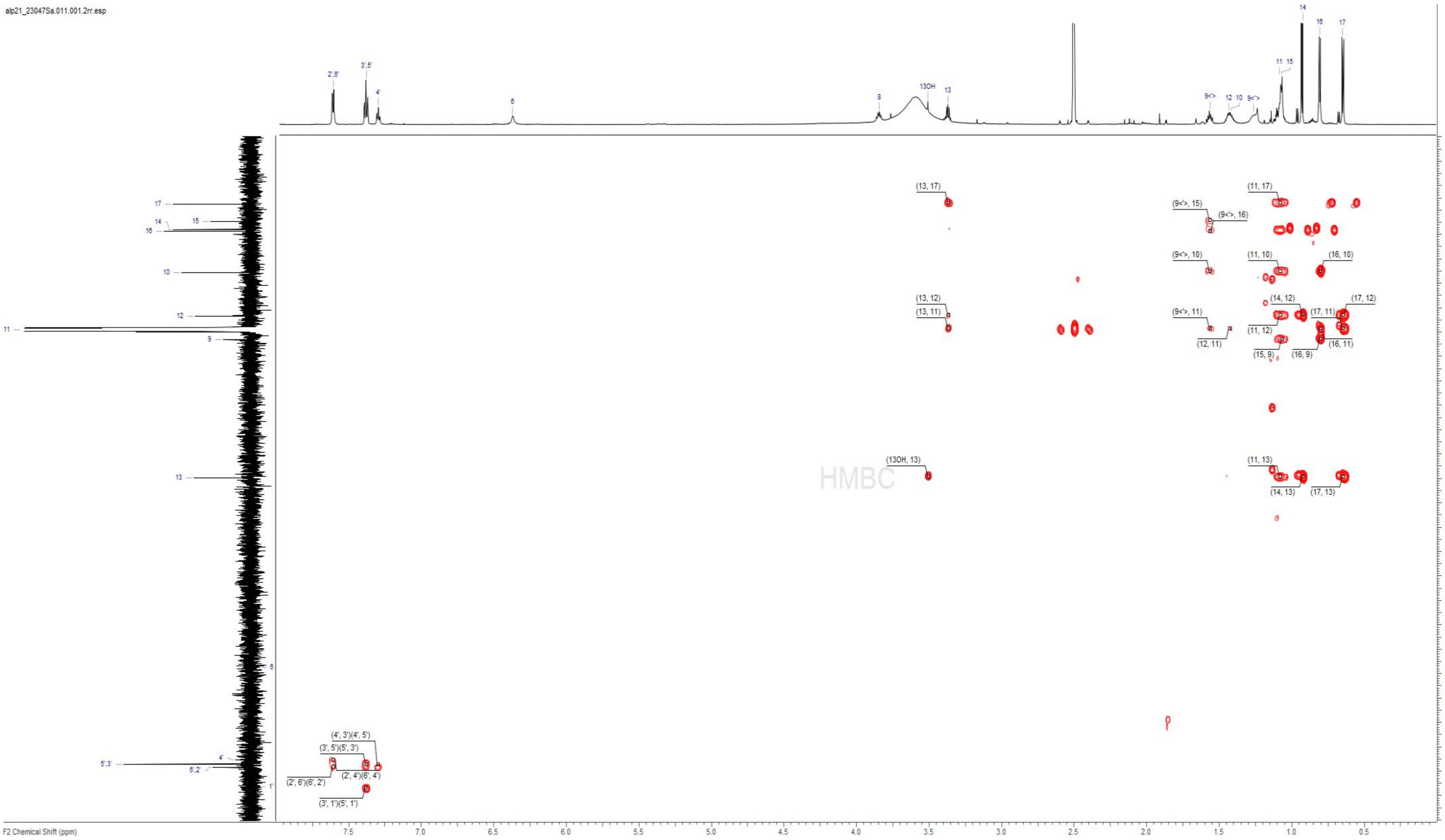

Figure S13. HMBC NMR spectrum of **2** (700 MHz, DMSO-d_6).

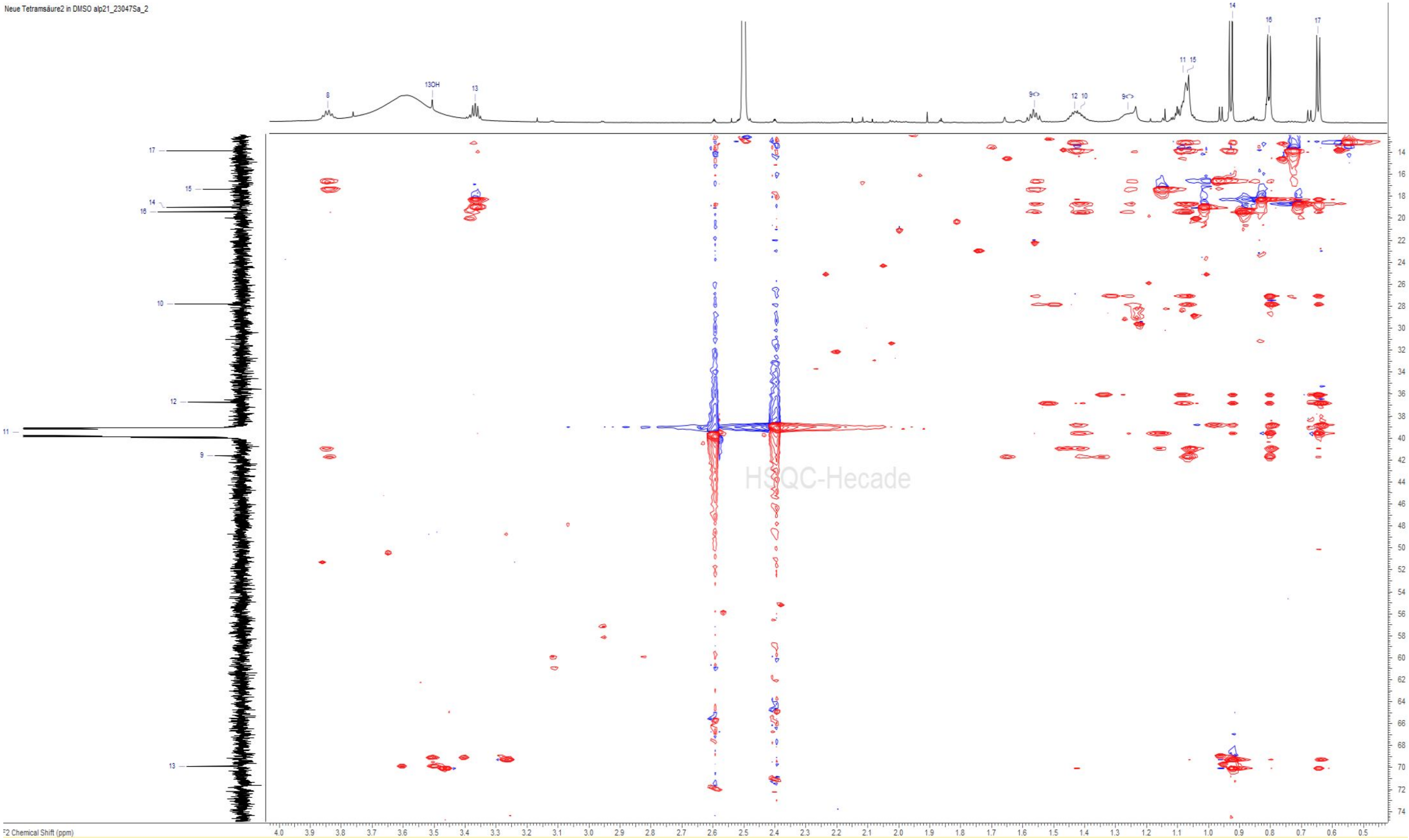

Figure S14. HSQC-Hecade NMR spectrum of **2** (700 MHz, DMSO-d_6).

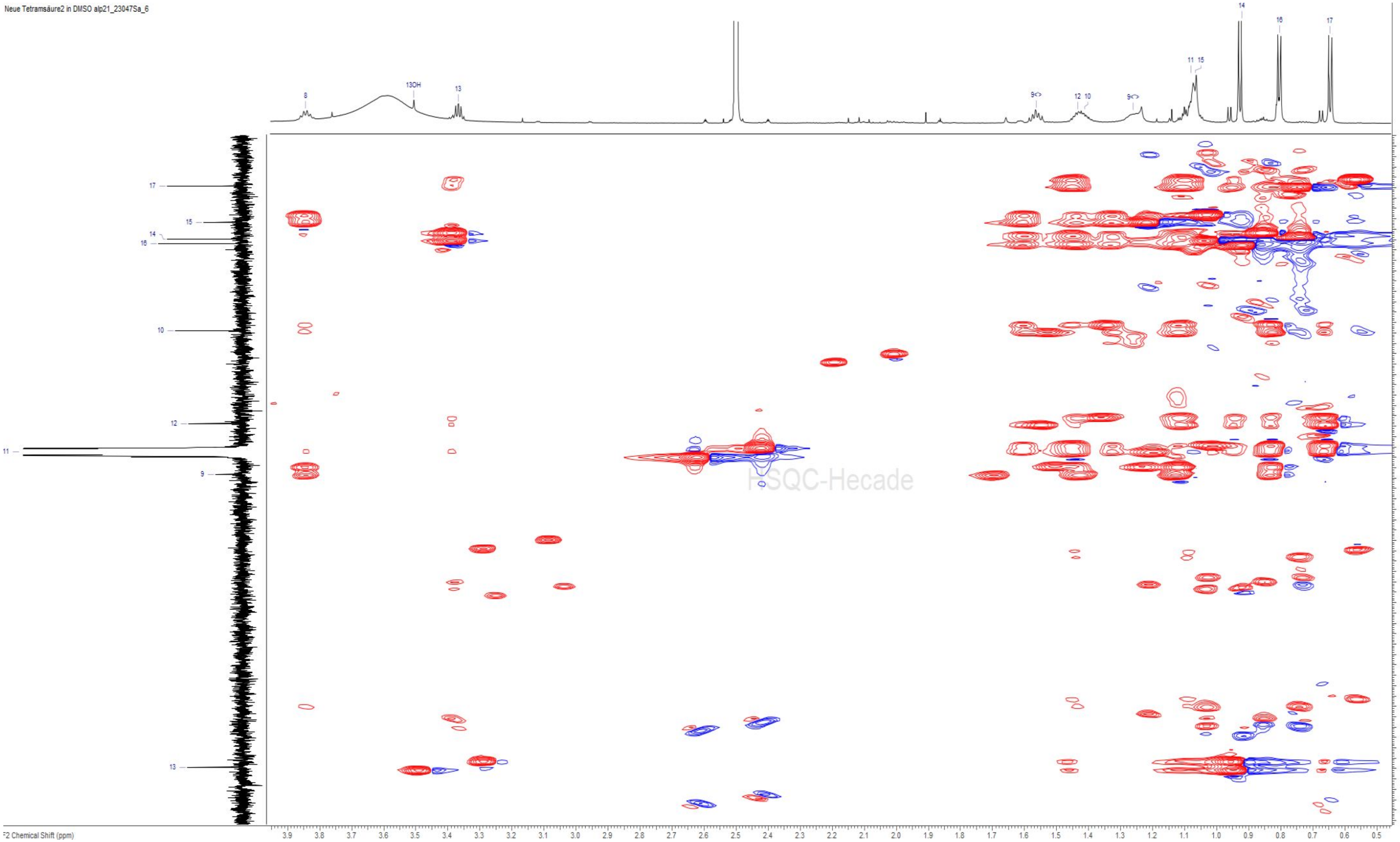

Figure S15. J-HMBC NMR spectrum of **2** (700 MHz, DMSO-d_6).

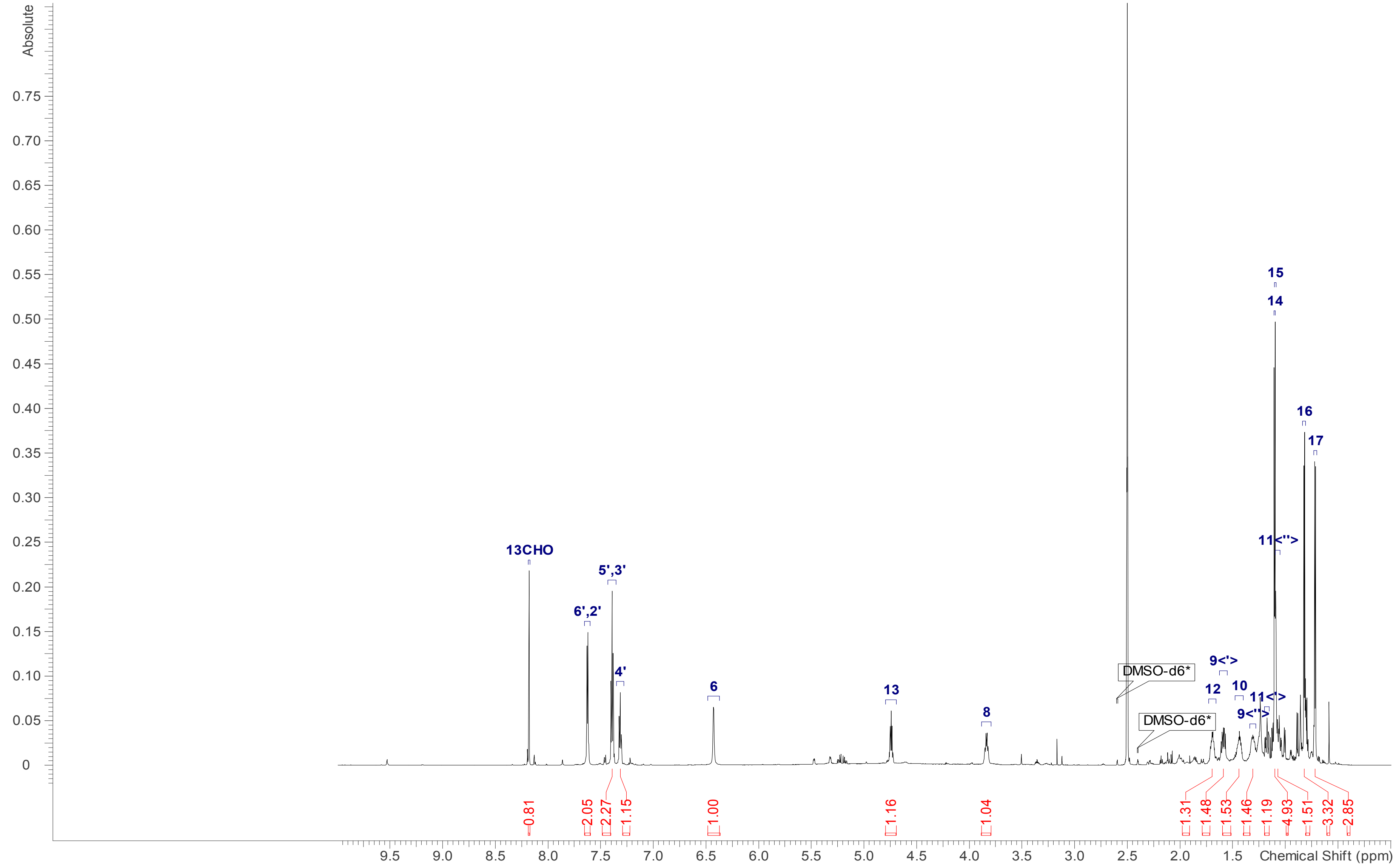

Figure S16. ^{1}H NMR spectrum of **3** (700 MHz, DMSO-d_6).

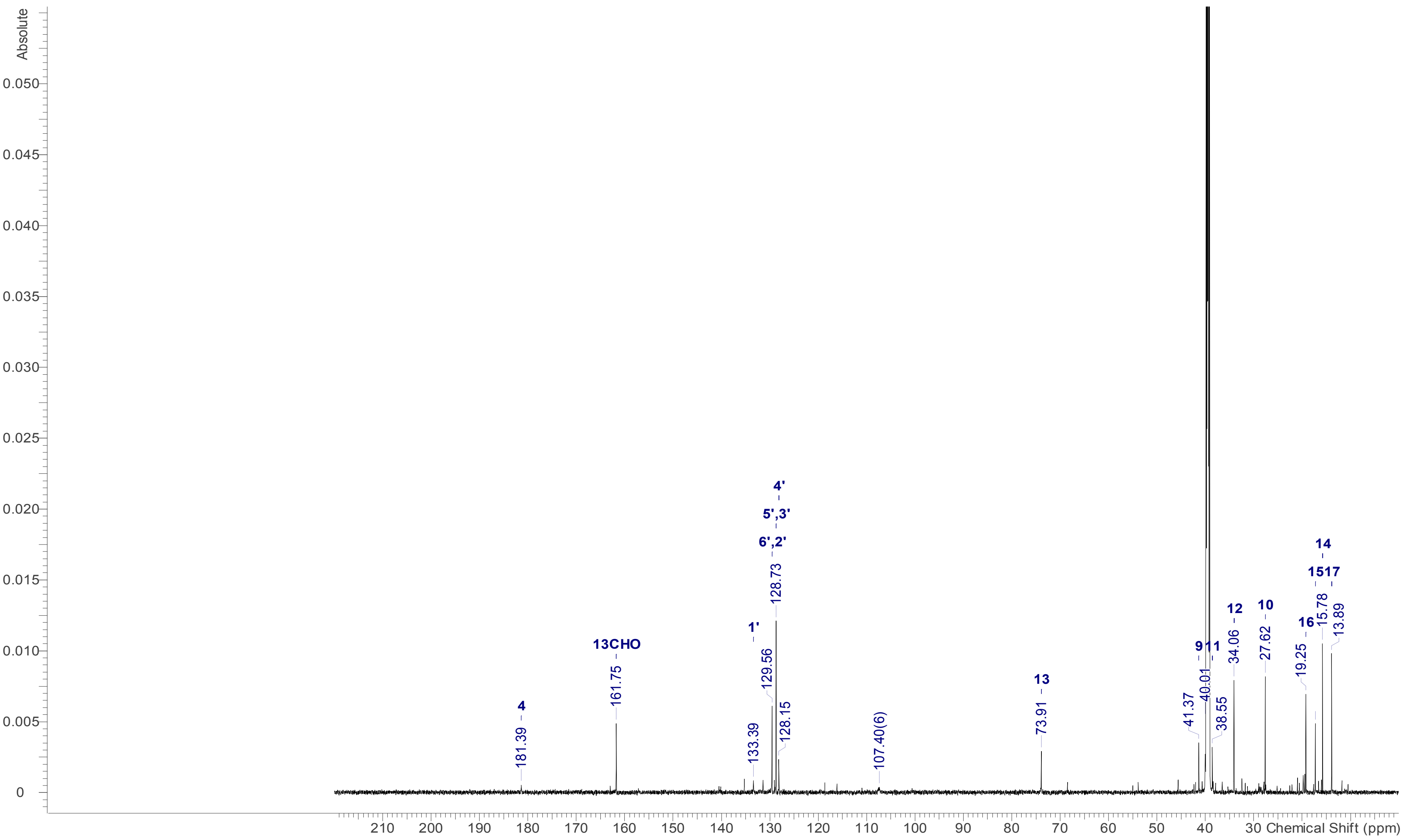

Figure S17. ^{13}C NMR spectrum of **3** (175 MHz, DMSO-d_6).

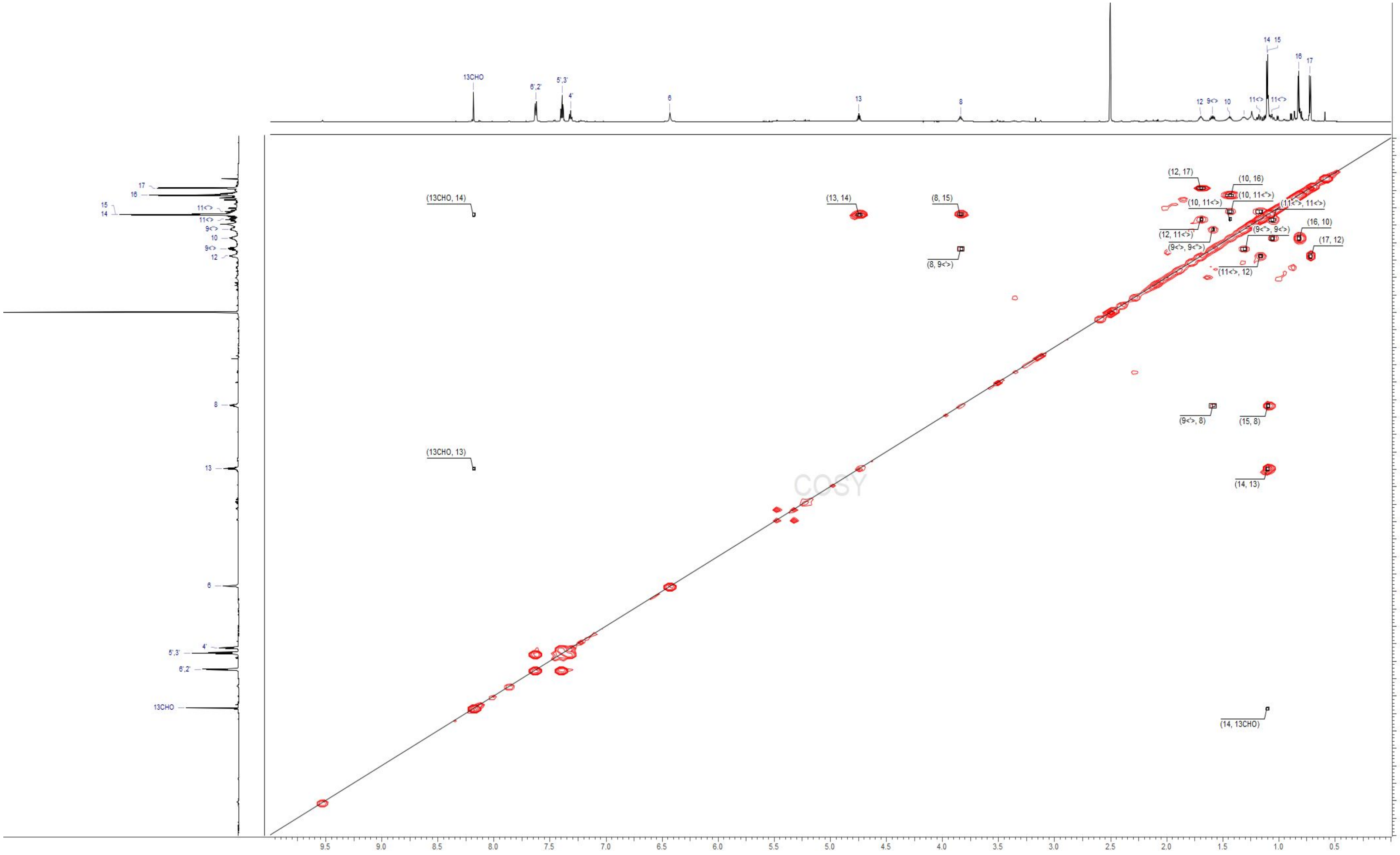

Figure S18. COSY NMR spectrum of **3** (700 MHz, DMSO-d_6).

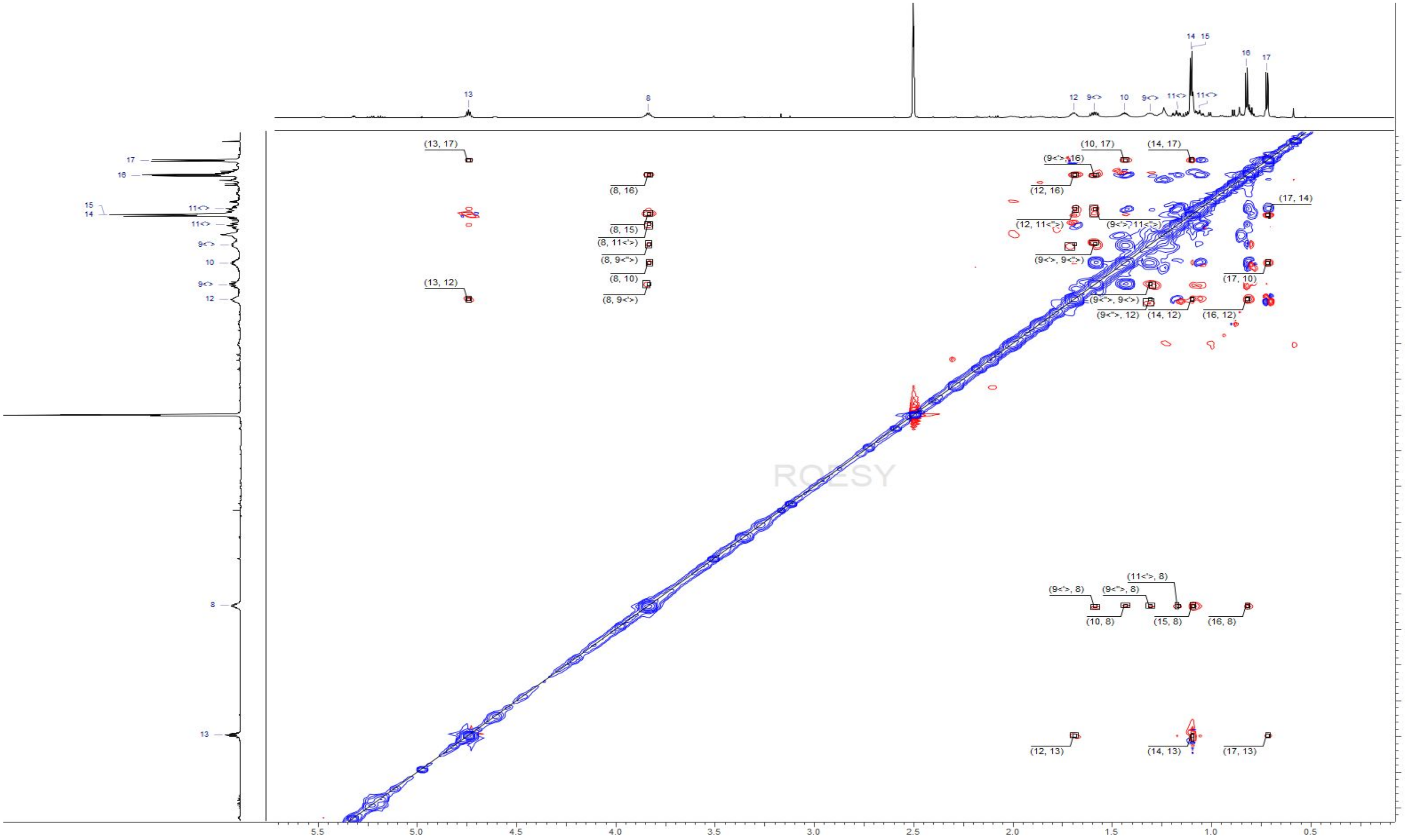

Figure S19. ROESY NMR spectrum of **3** (700 MHz, DMSO-d_6).

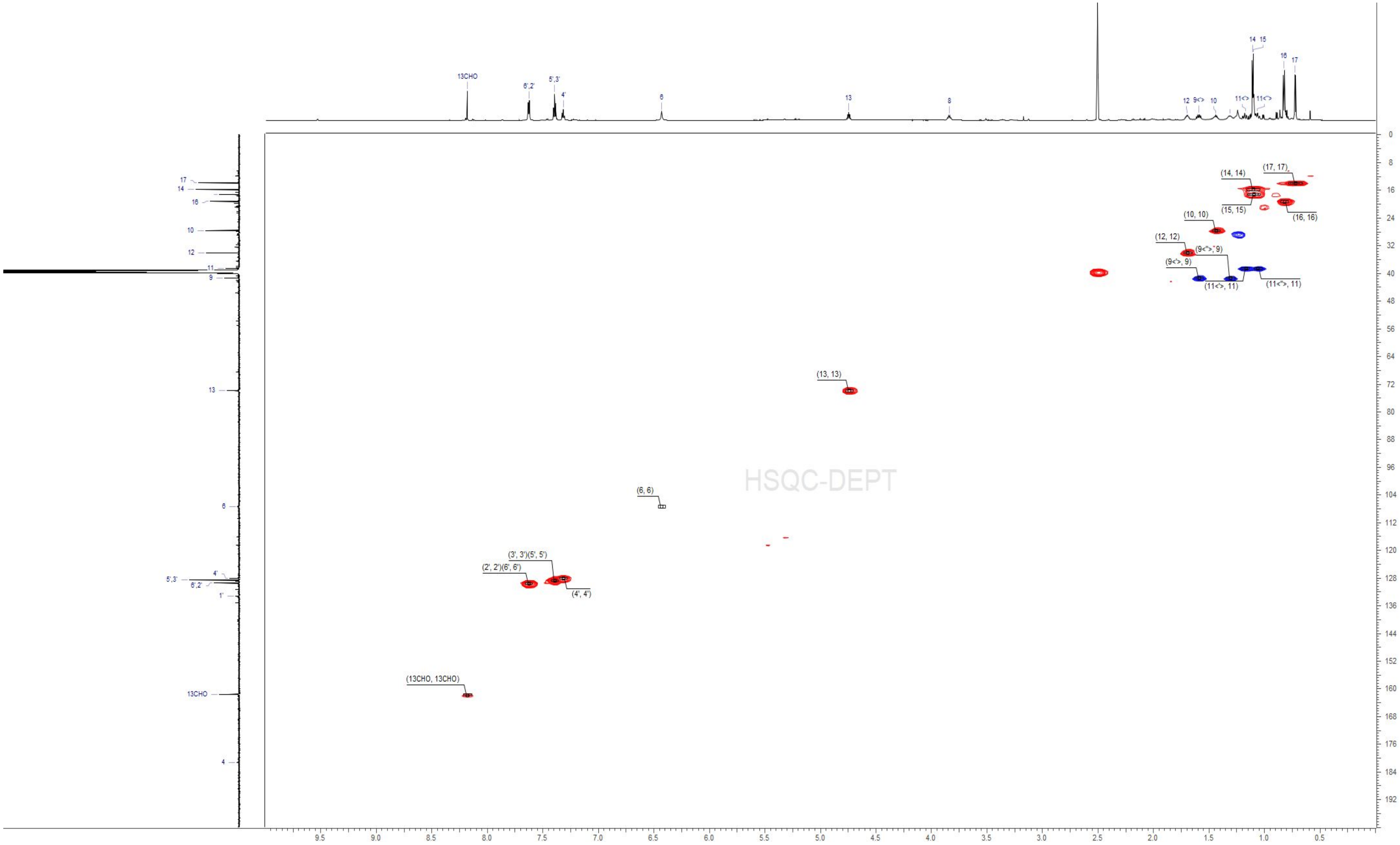

Figure S20. HSQC NMR spectrum of **3** (700 MHz, DMSO-d_6).

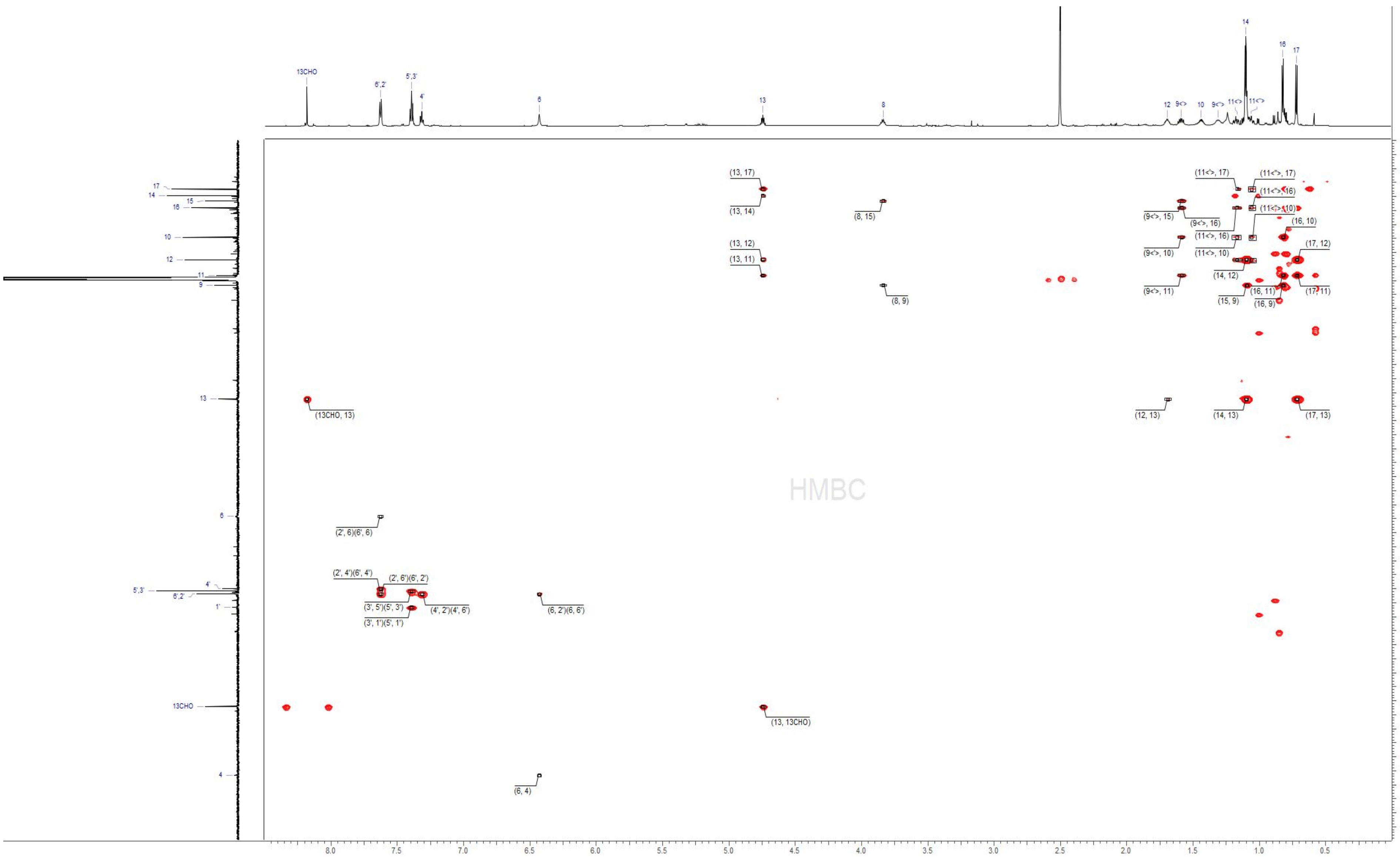

Figure S21. HMBC NMR spectrum of **3** (700 MHz, DMSO-d_6).

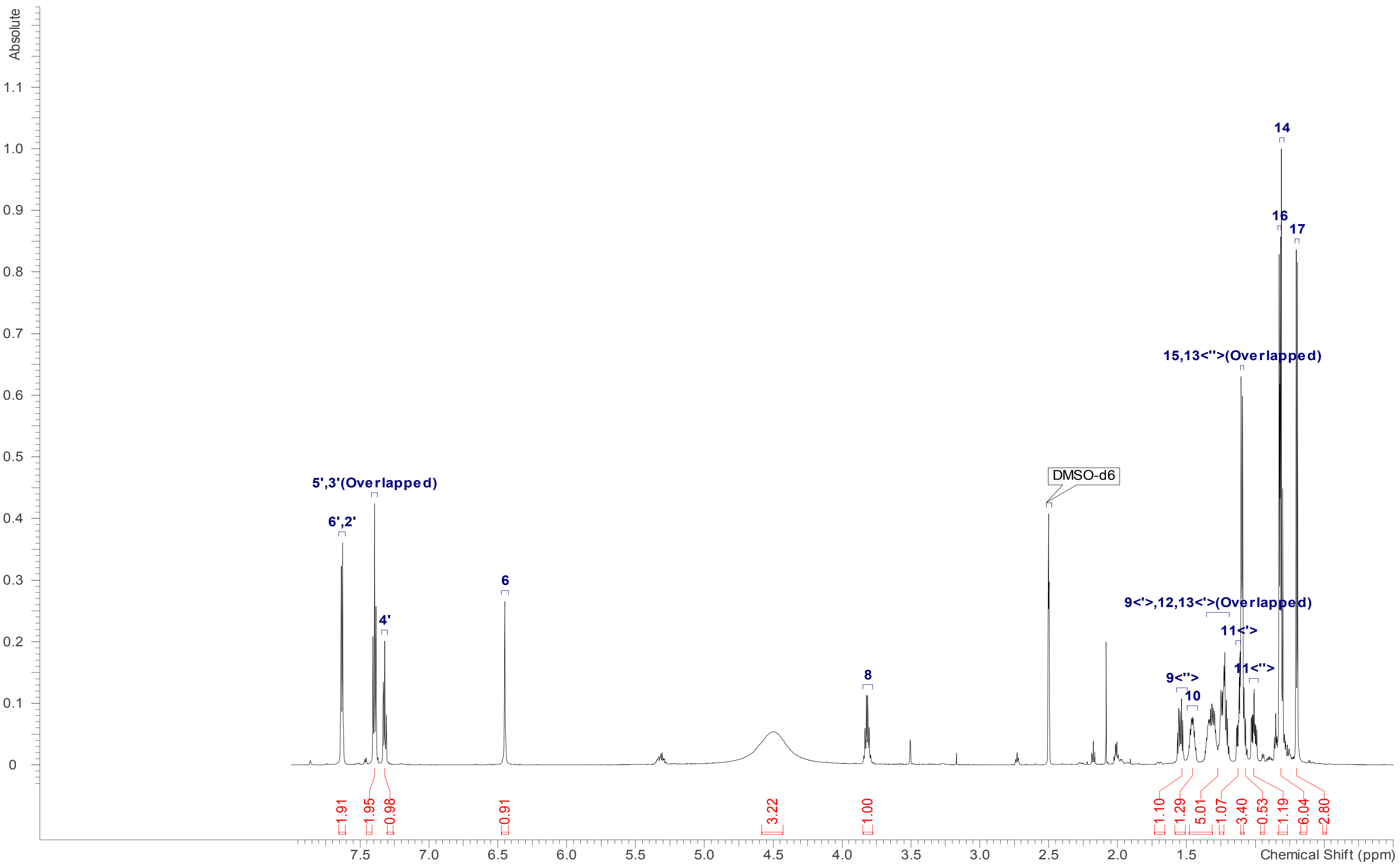

Figure S22. ^{1}H NMR spectrum of **4** (700 MHz, DMSO-d_6).

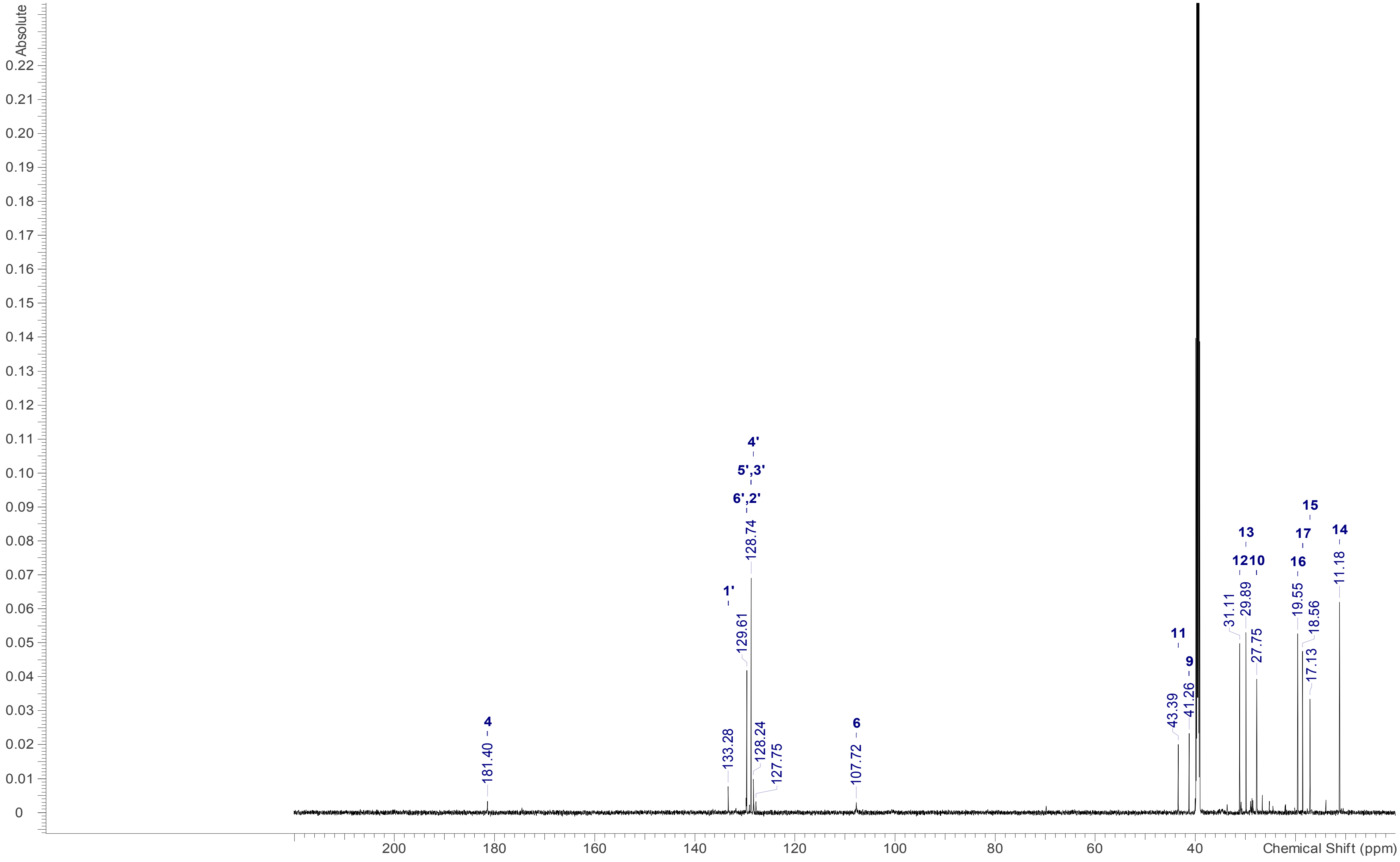

Figure S23. ^{13}C NMR spectrum of **4** (175 MHz, DMSO-d_6).

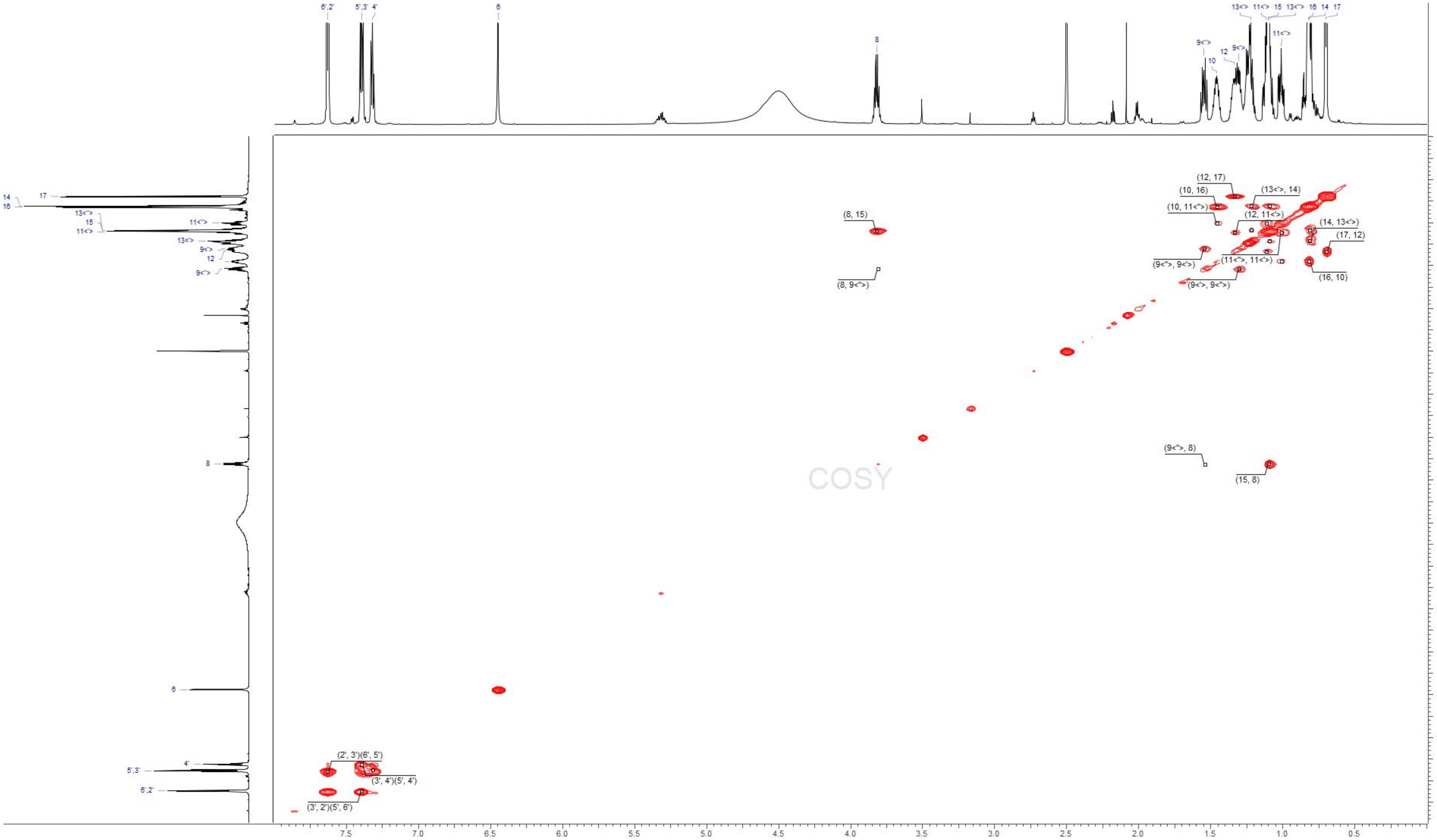

Figure S24. COSY NMR spectrum of **4** (700 MHz, DMSO-d_6).

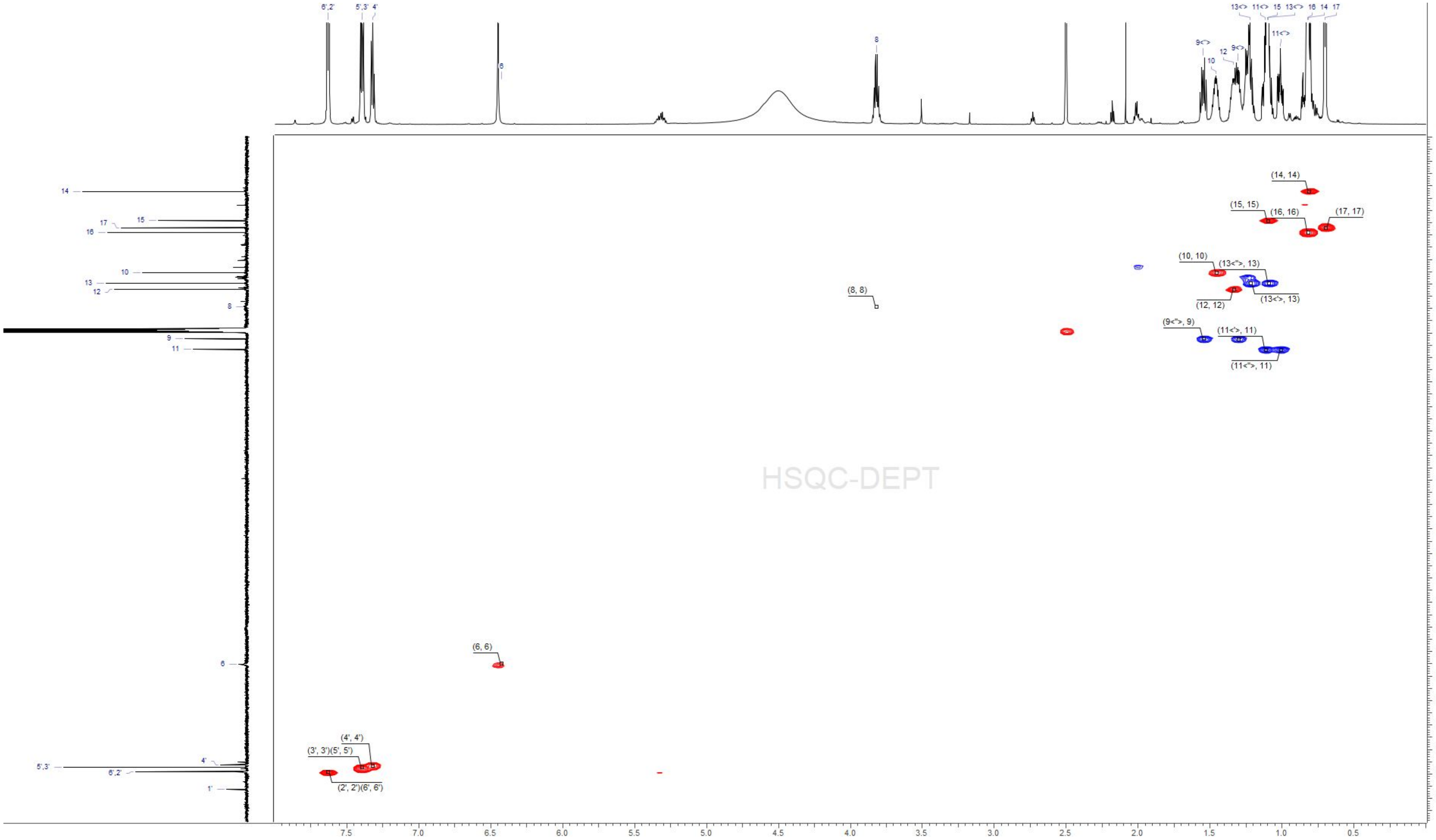

Figure S25. HSQC NMR spectrum of **4** (700 MHz, DMSO-d_6).

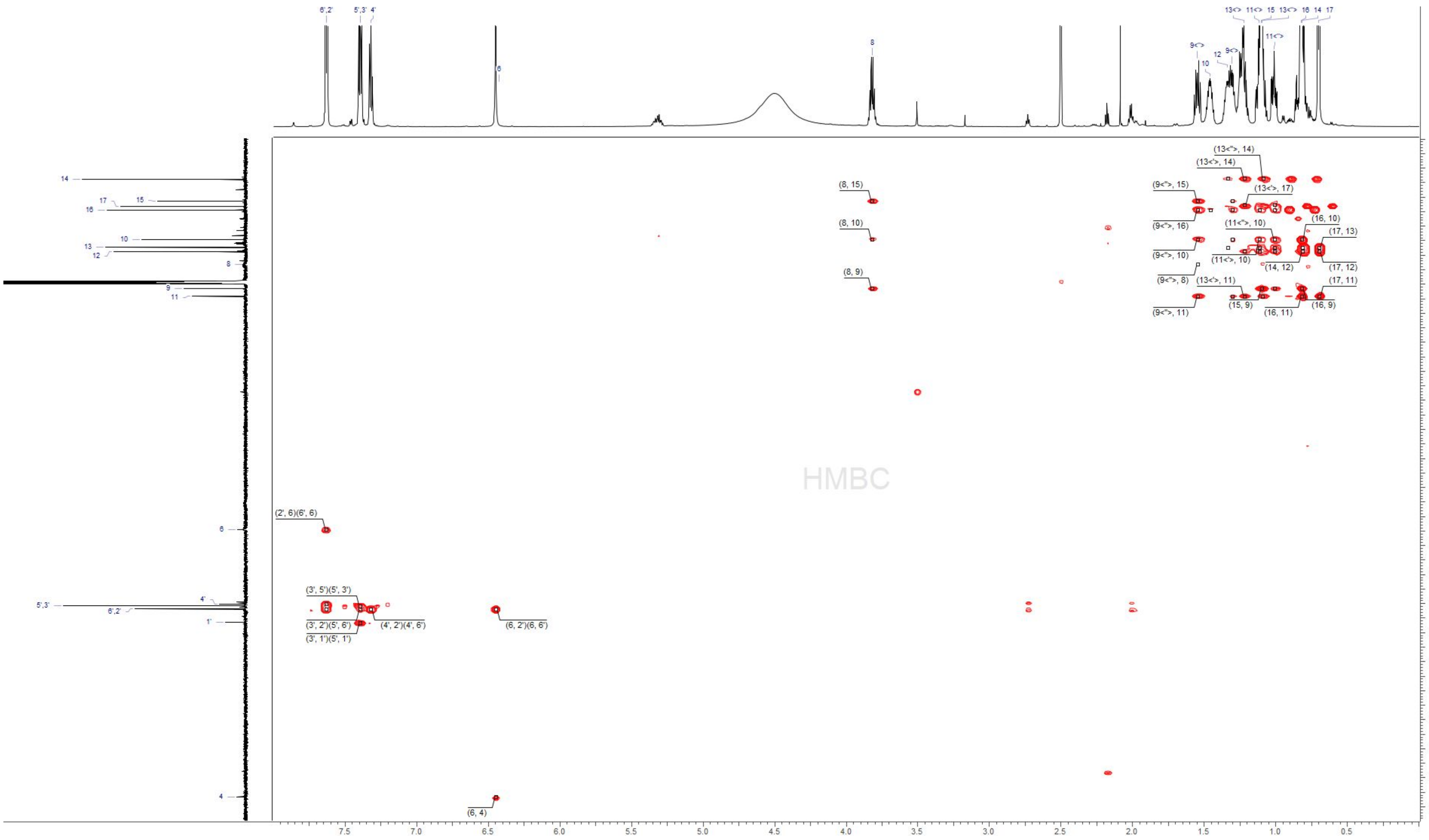

Figure S26. HMBC NMR spectrum of **4** (700 MHz, DMSO-d_6).

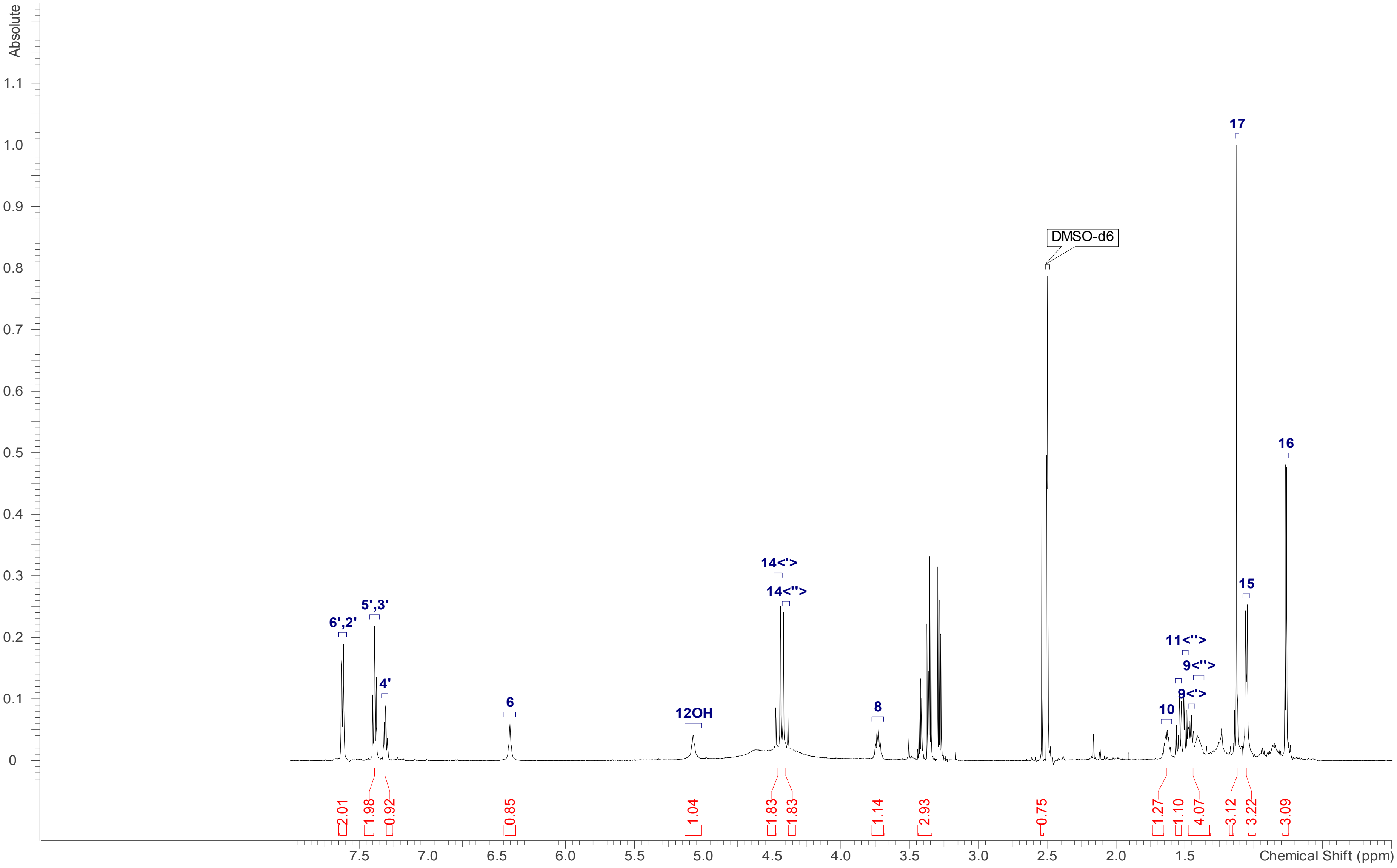

Figure S27. ^{1}H NMR spectrum of **5** (700 MHz, DMSO-d_6).

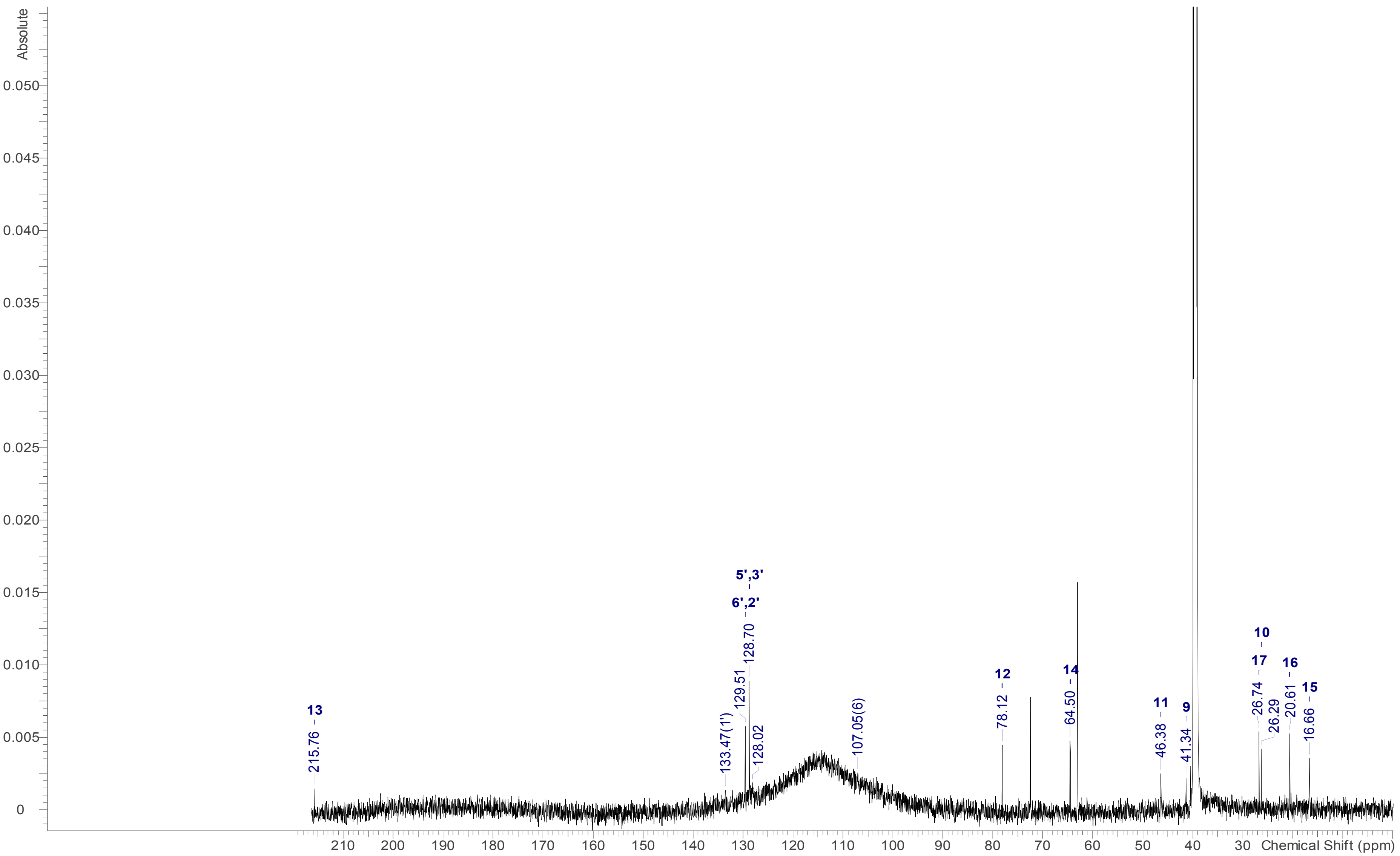

Figure S28. ^{13}C NMR spectrum of **5** (175 MHz, DMSO-d_6).

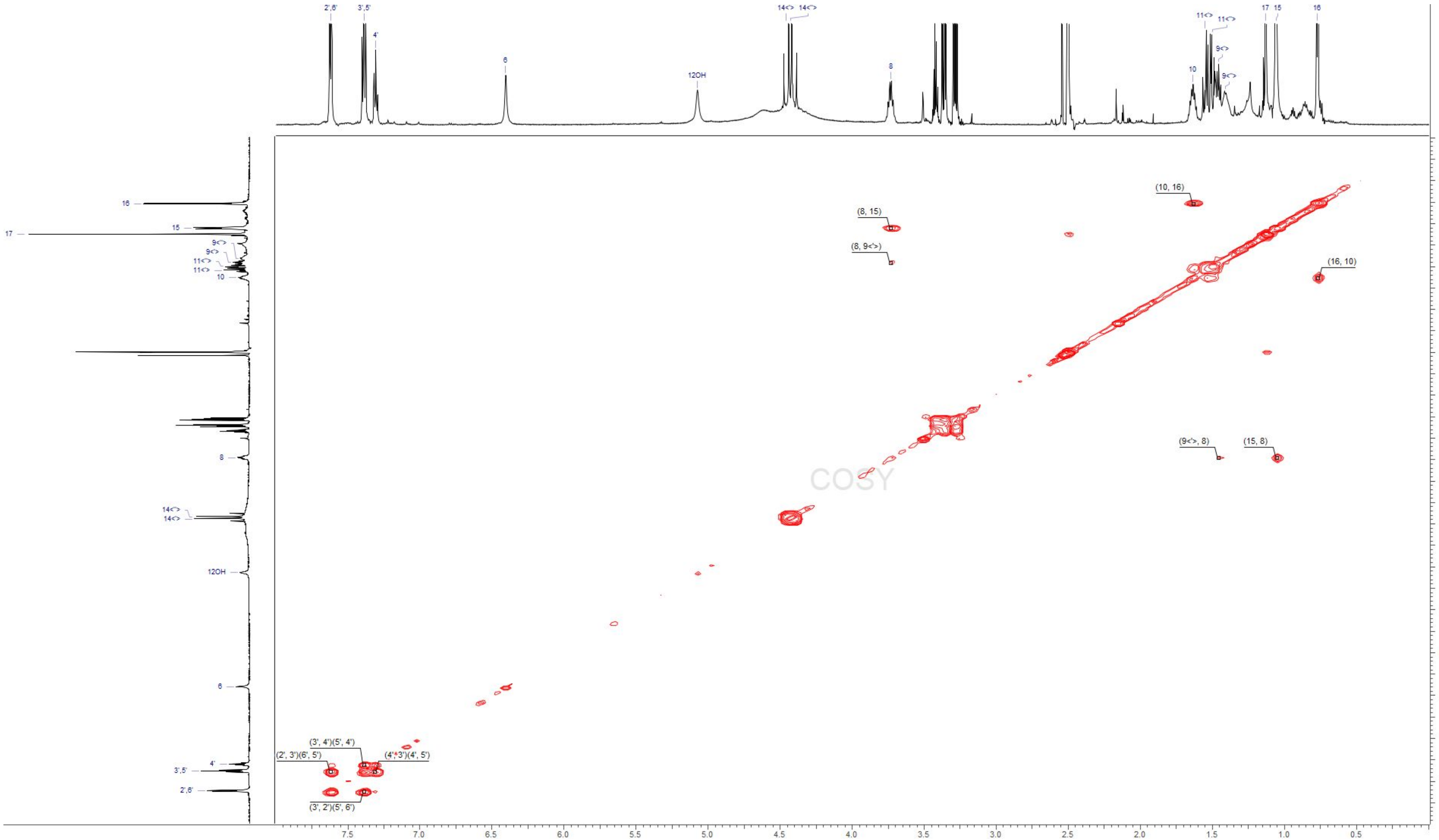

Figure S29. COSY NMR spectrum of **5** (700 MHz, DMSO-d_6).

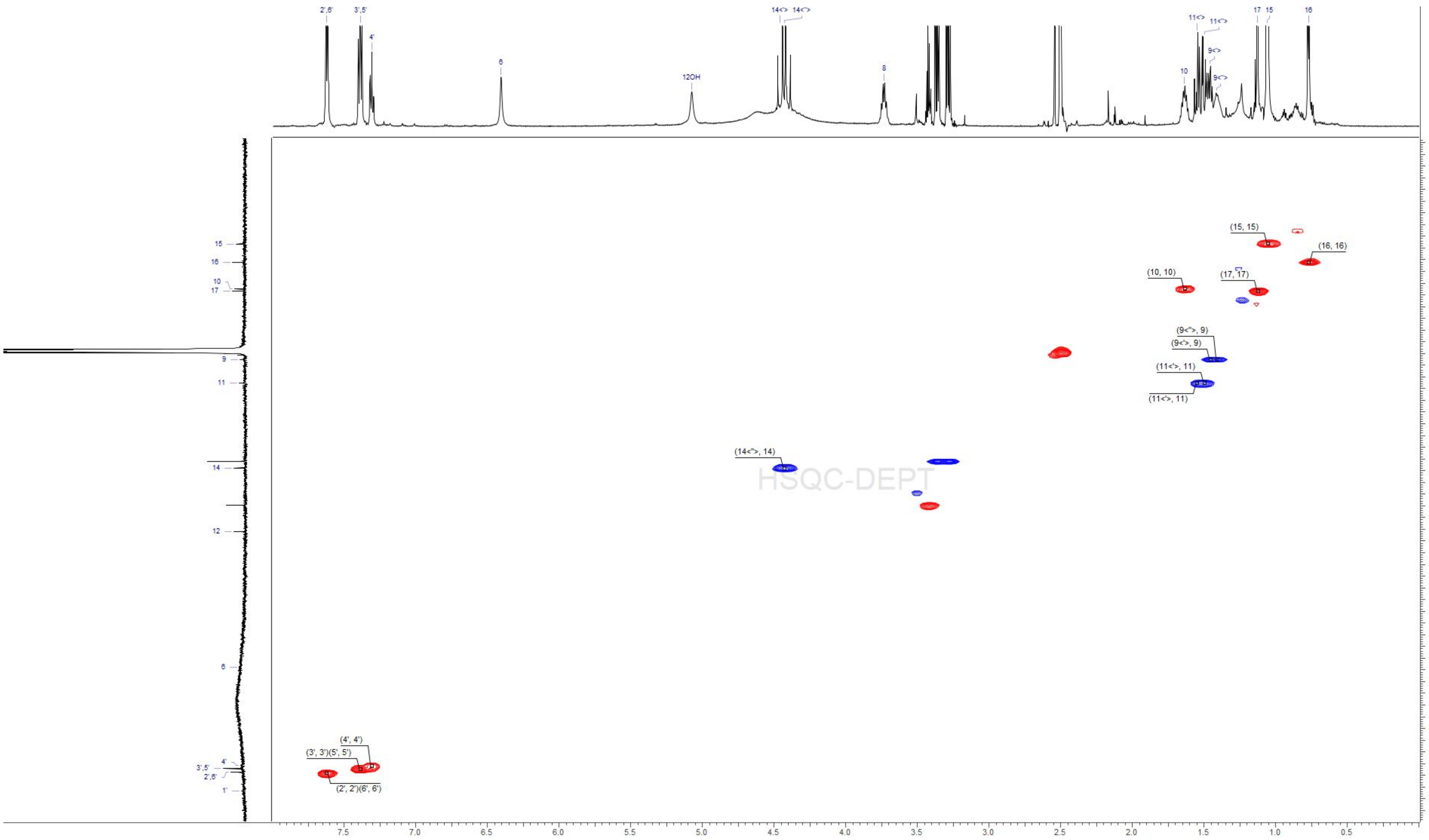

Figure S30. HSQC NMR spectrum of **5** (700 MHz, DMSO-d_6).

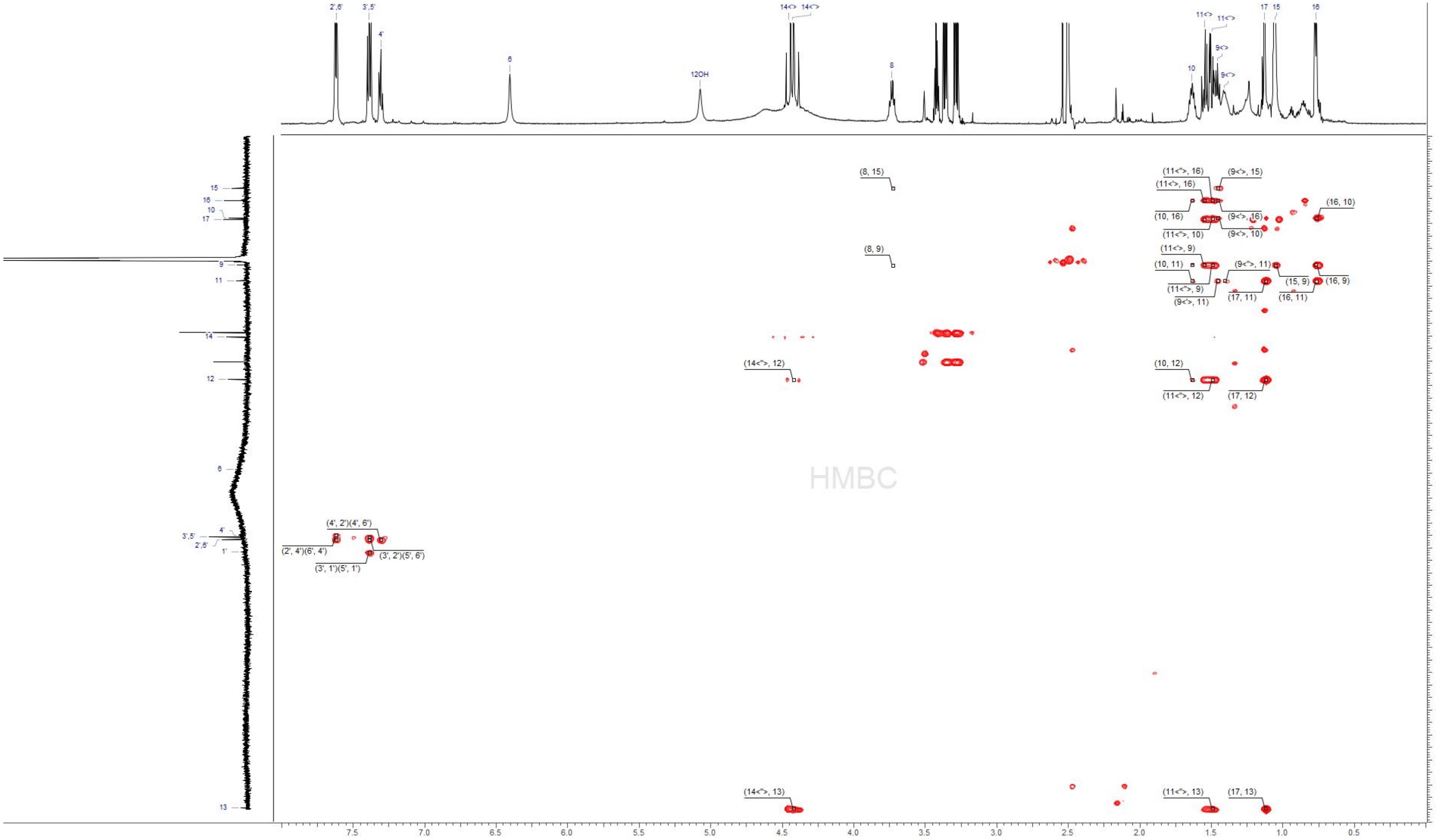

Figure S31. HMBC NMR spectrum of **5** (700 MHz, DMSO-d_6).

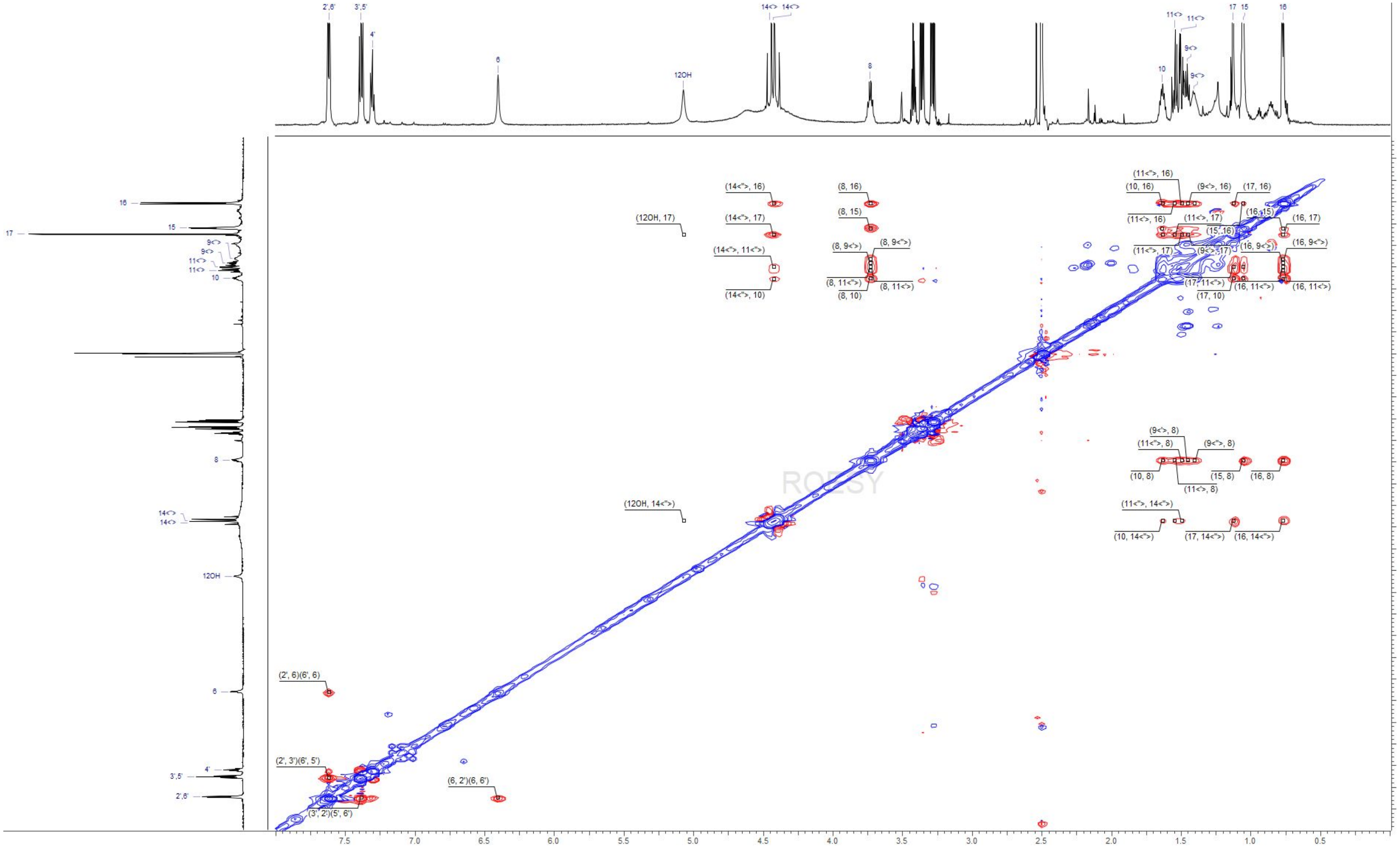

Figure S32. ROESY NMR spectrum of **5** (700 MHz, DMSO-d_6).

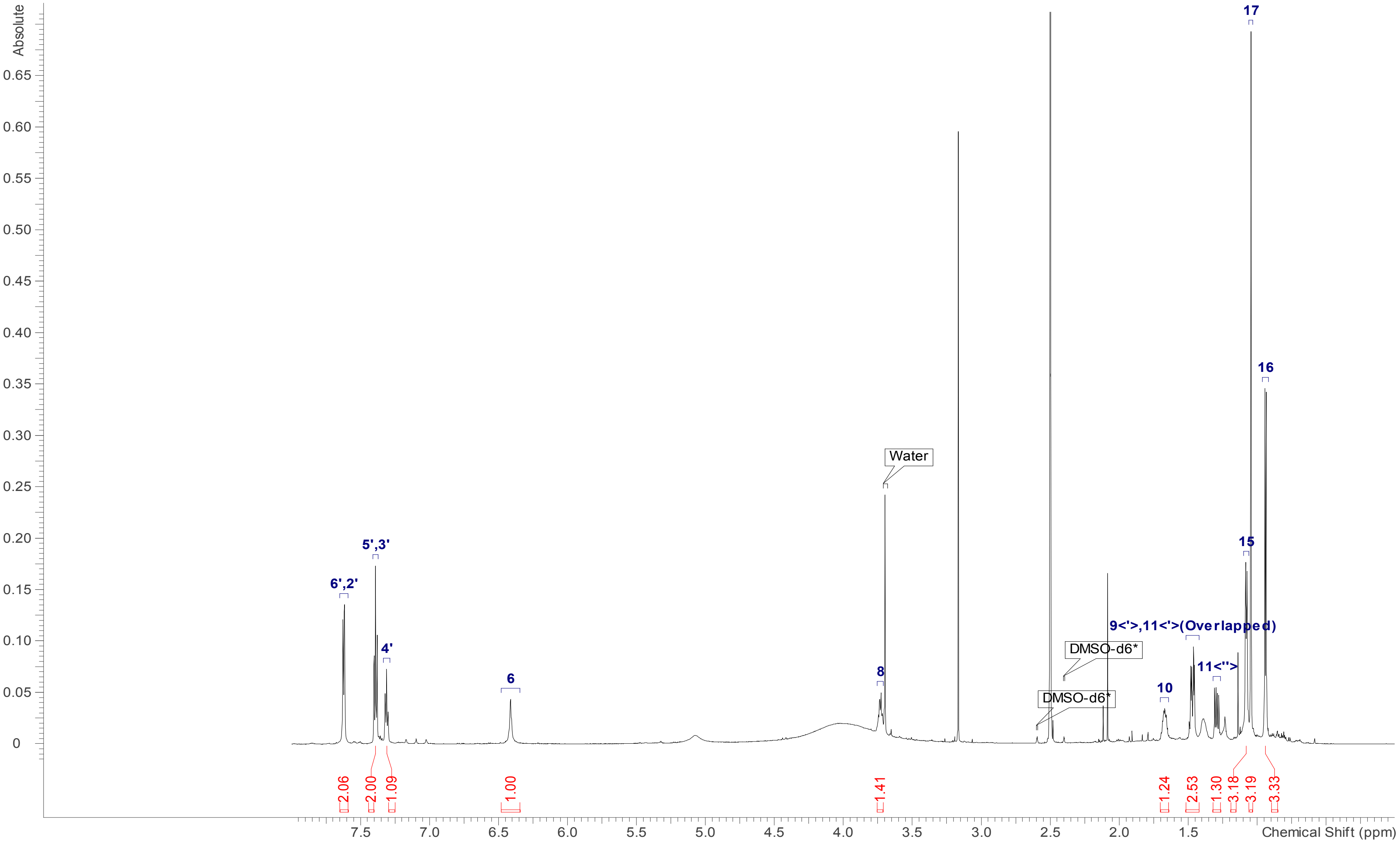

Figure S33. ^{1}H NMR spectrum of **6** (700 MHz, DMSO-d_6).

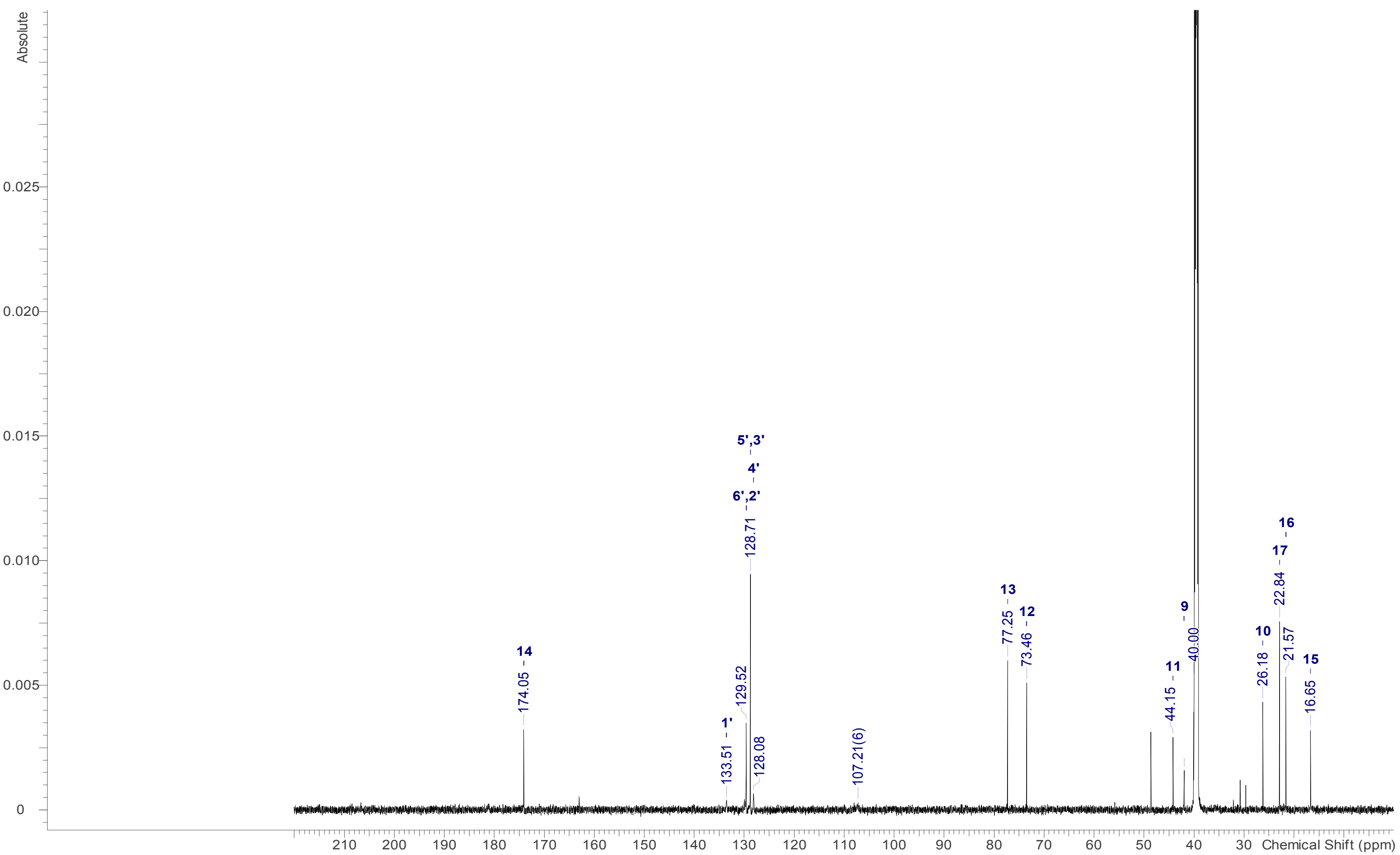

Figure S34. ^{13}C NMR spectrum of **6** (175 MHz, DMSO-d_6).

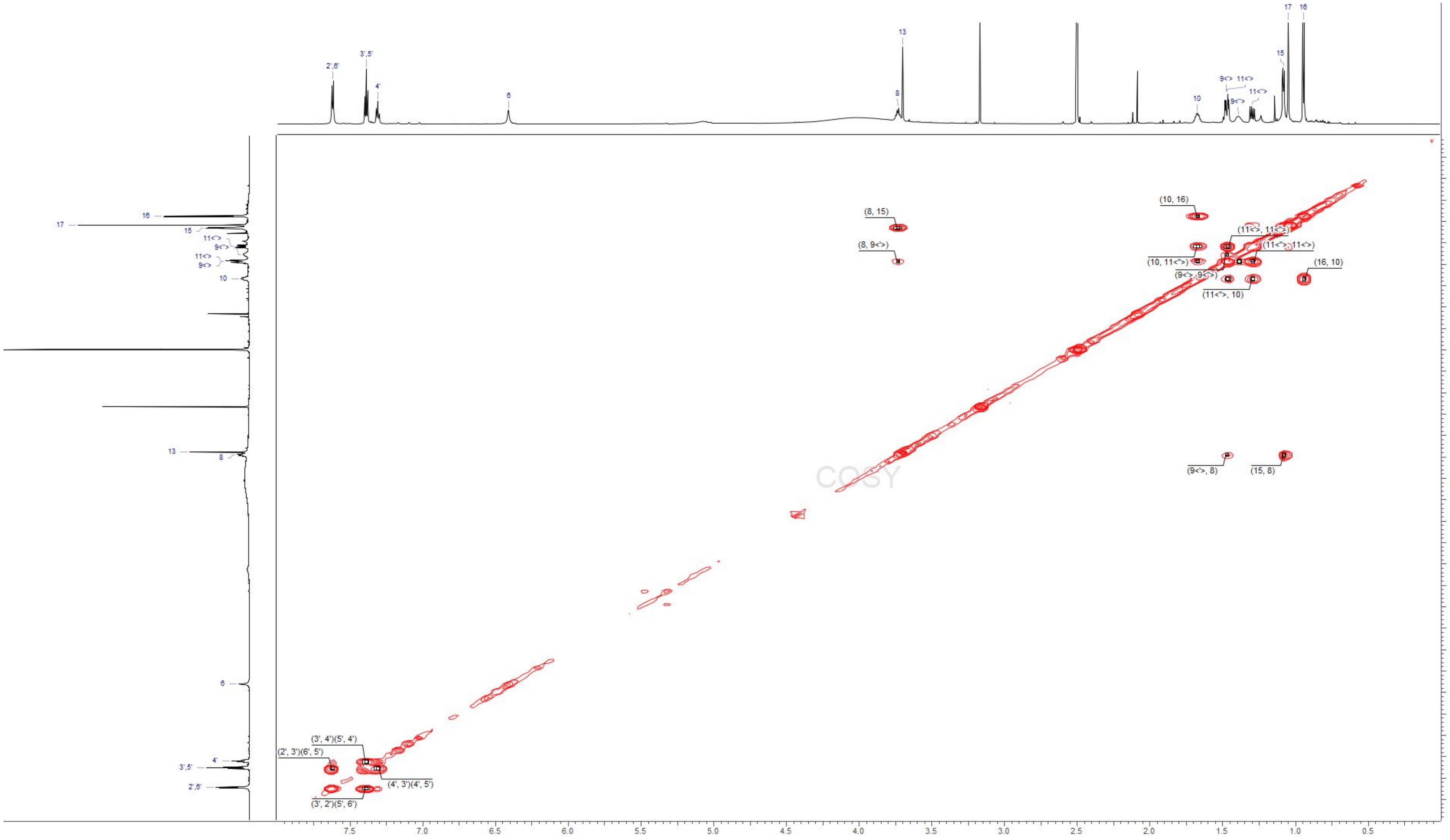

Figure S35. COSY NMR spectrum of **6** (700 MHz, DMSO-d_6).

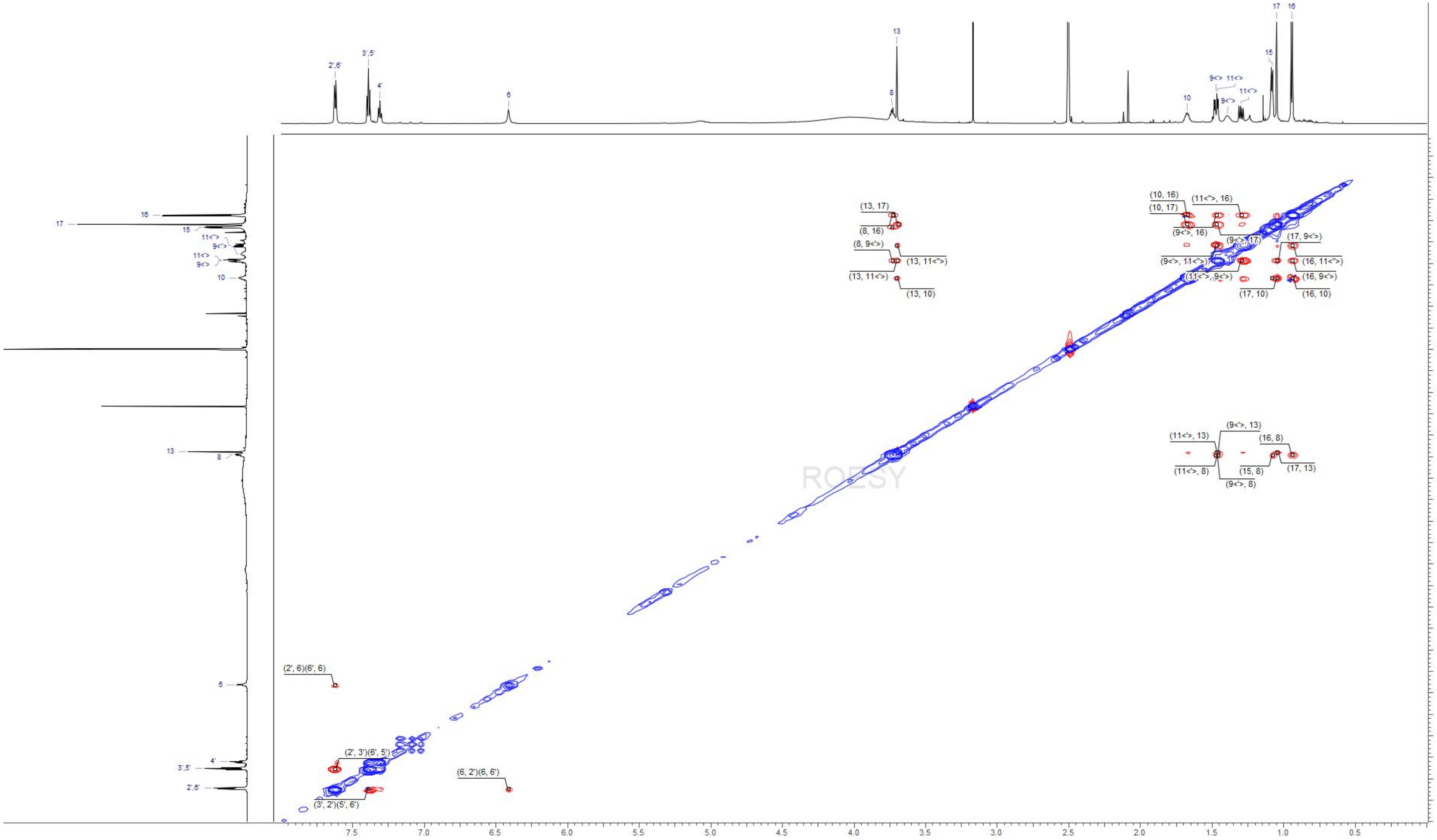

Figure S36. ROESY NMR spectrum of **6** (700 MHz, DMSO-d_6).

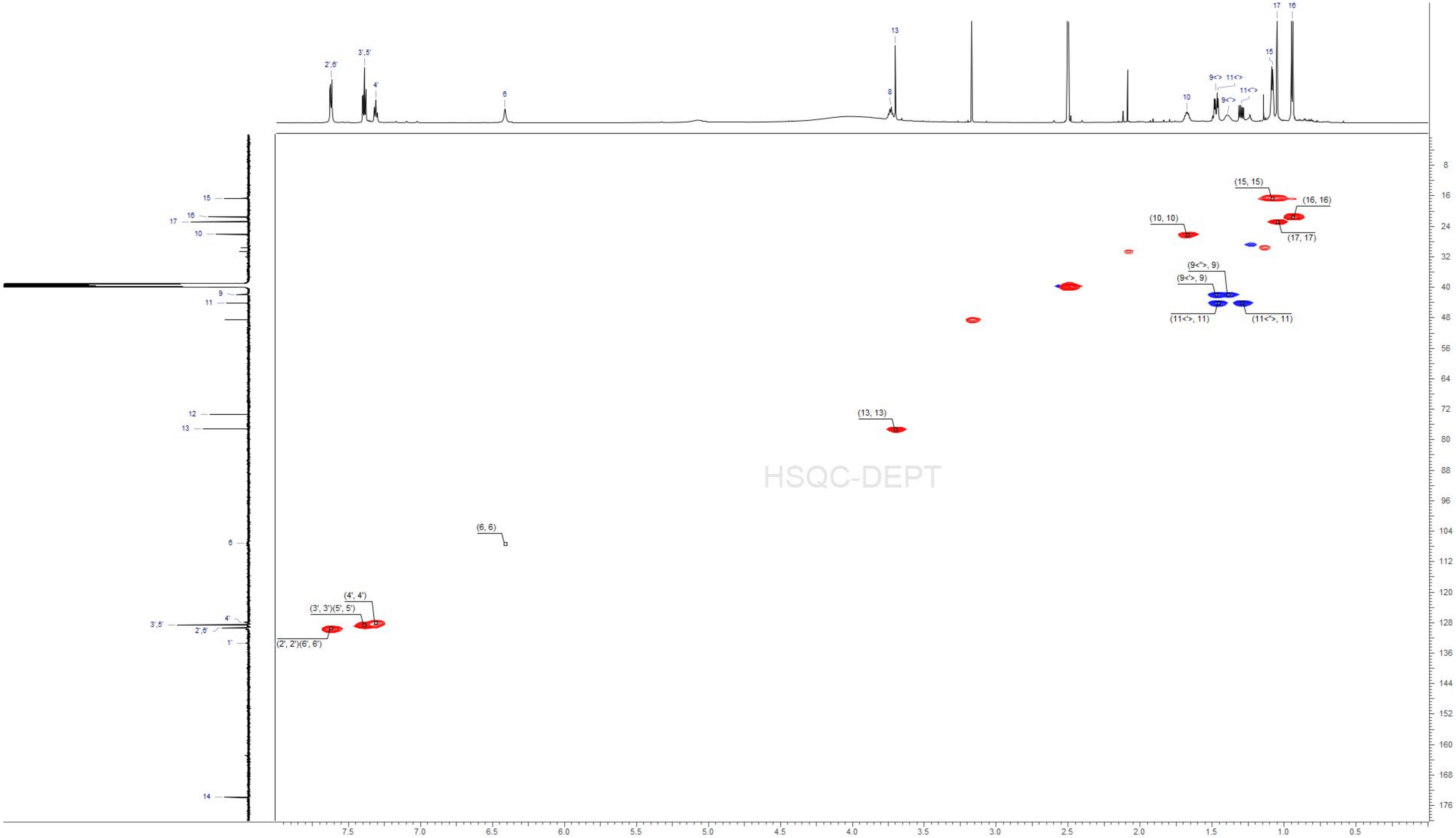

Figure S37. HSQC NMR spectrum of **6** (700 MHz, DMSO-d_6).

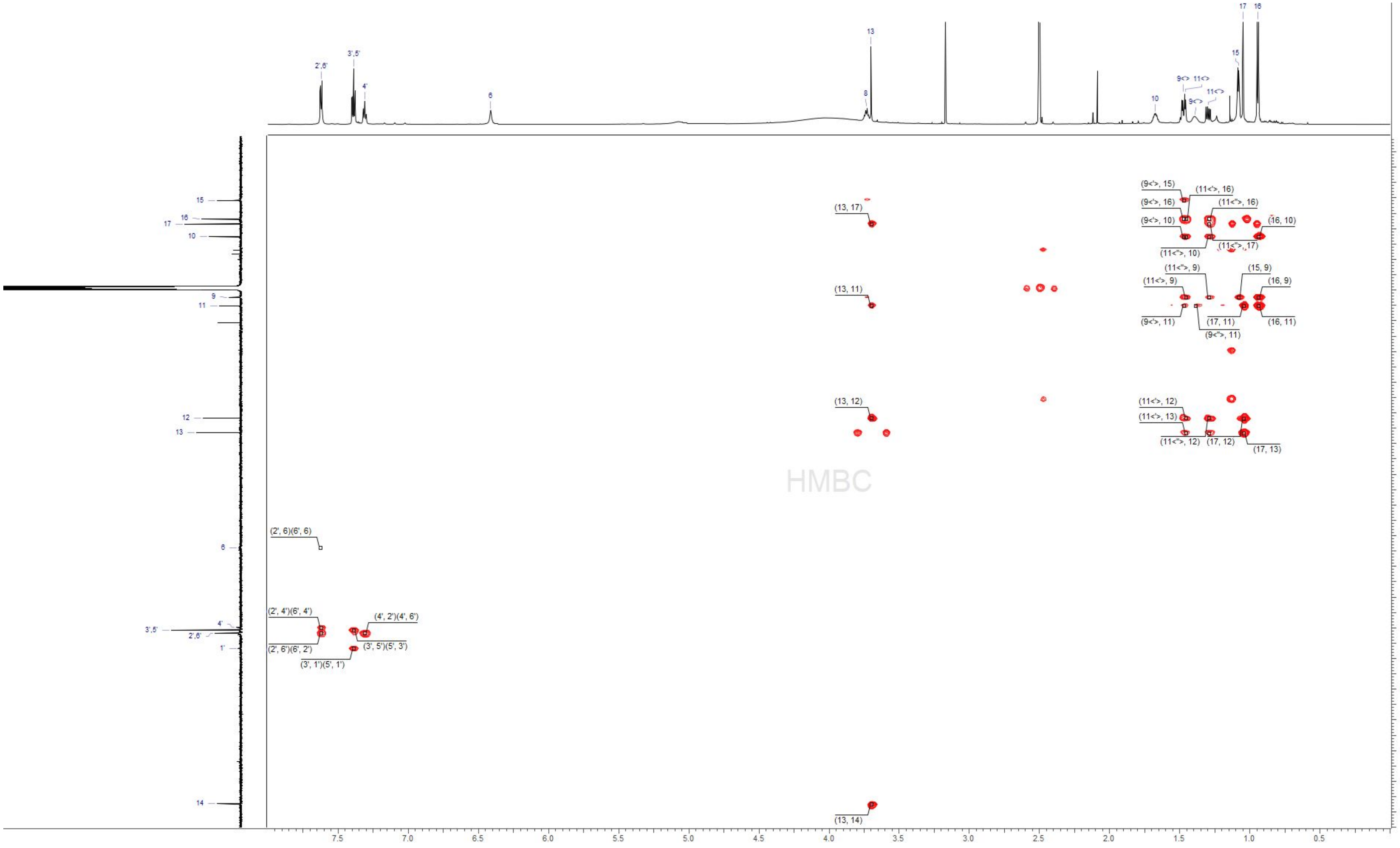

Figure S38. HMBC NMR spectrum of **6** (700 MHz, DMSO-d_6).

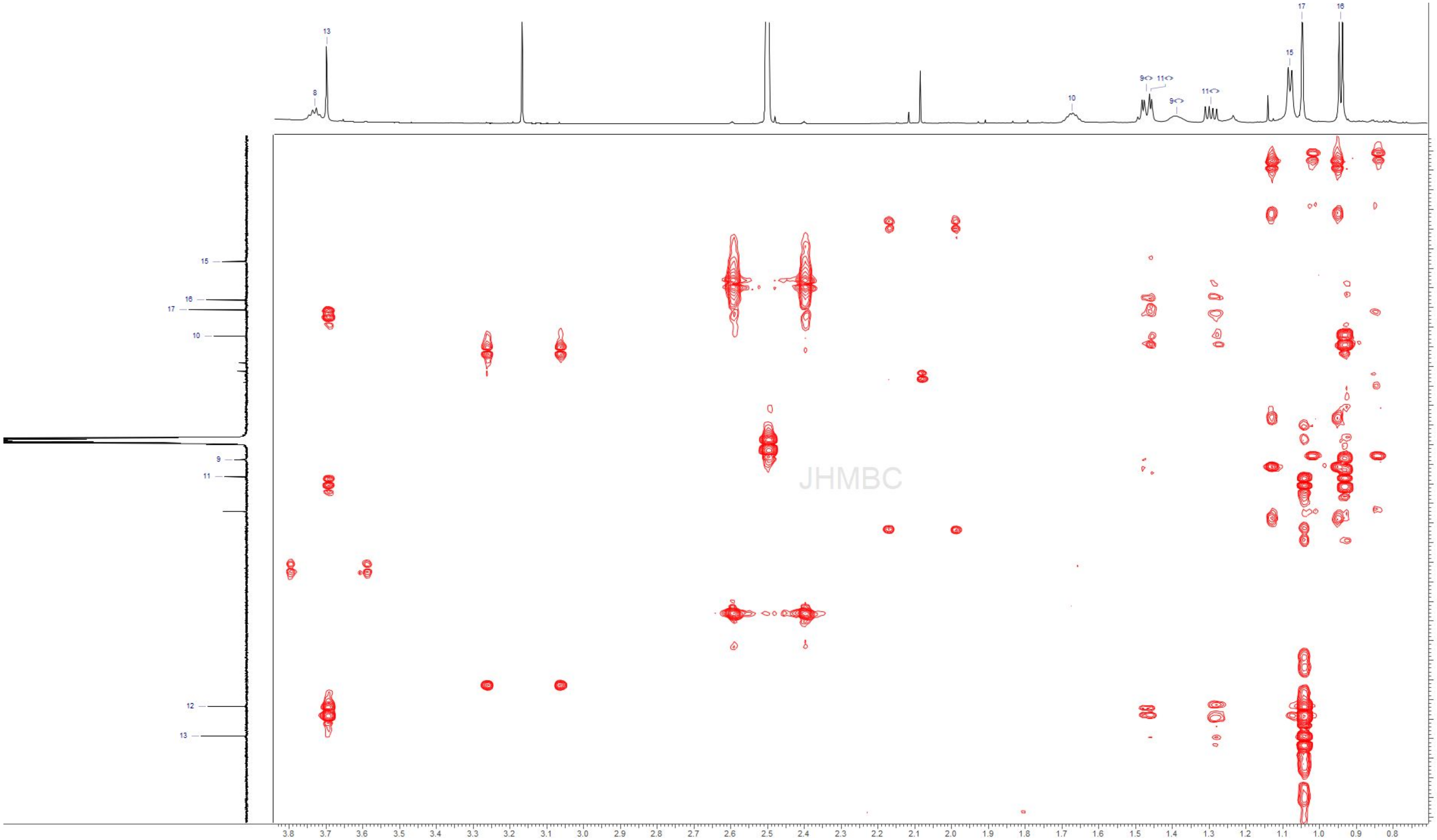

Figure S39. JHMBC NMR spectrum of **6** (700 MHz, DMSO-d_6).

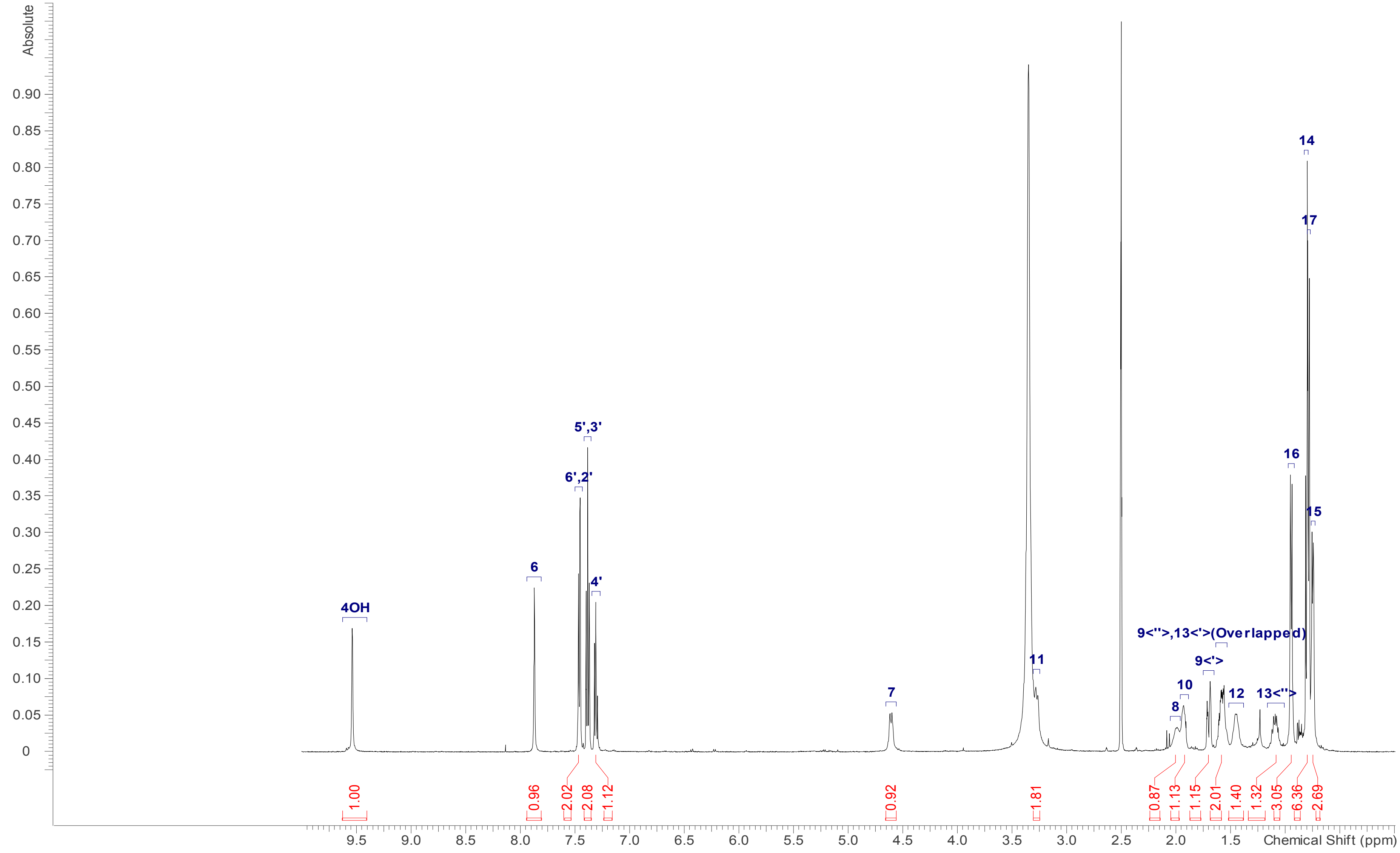

Figure S40. ^{1}H NMR spectrum of **7** (700 MHz, DMSO-d_6).

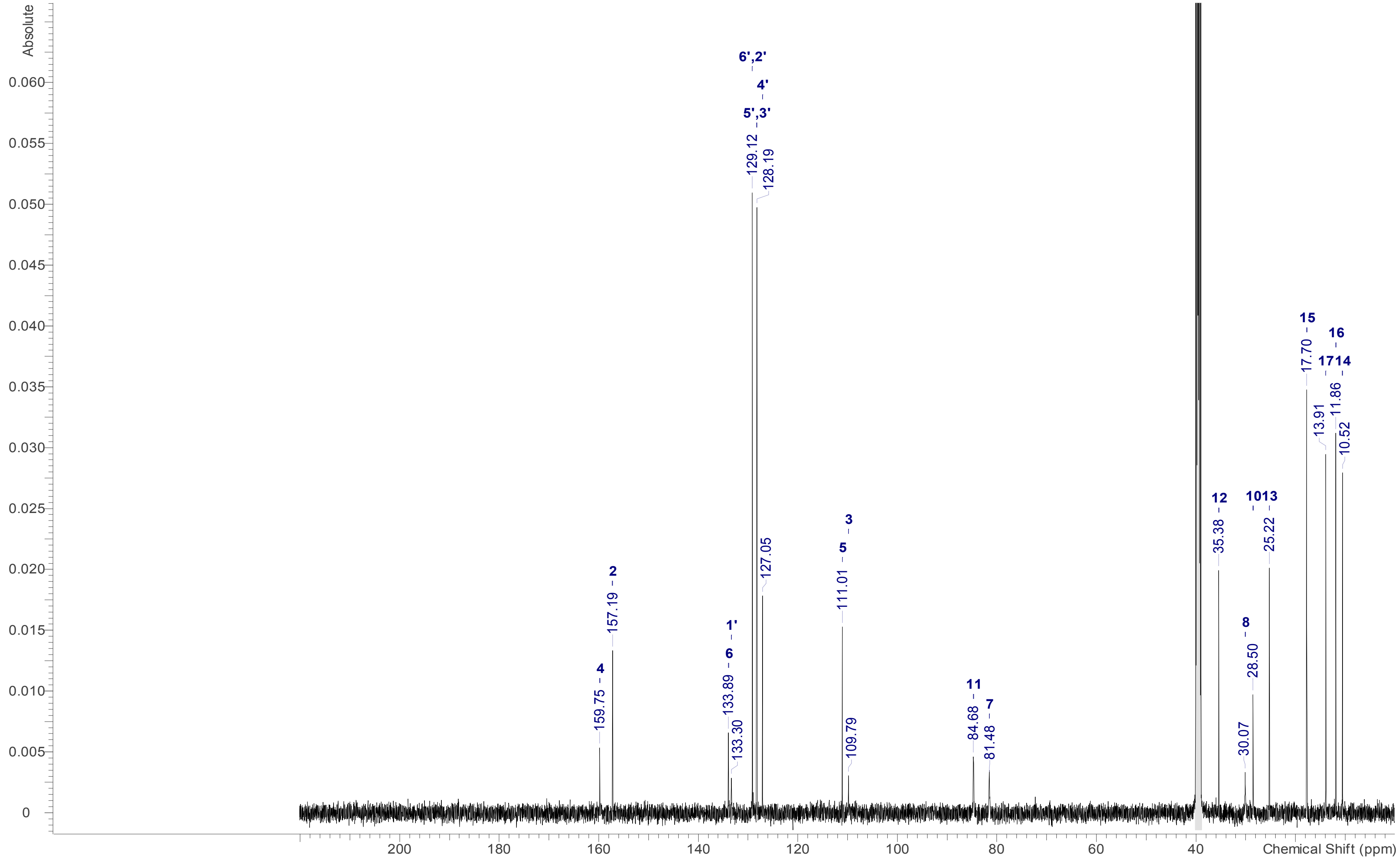

Figure S41. ^{13}C NMR spectrum of **7** (175 MHz, DMSO-d_6).

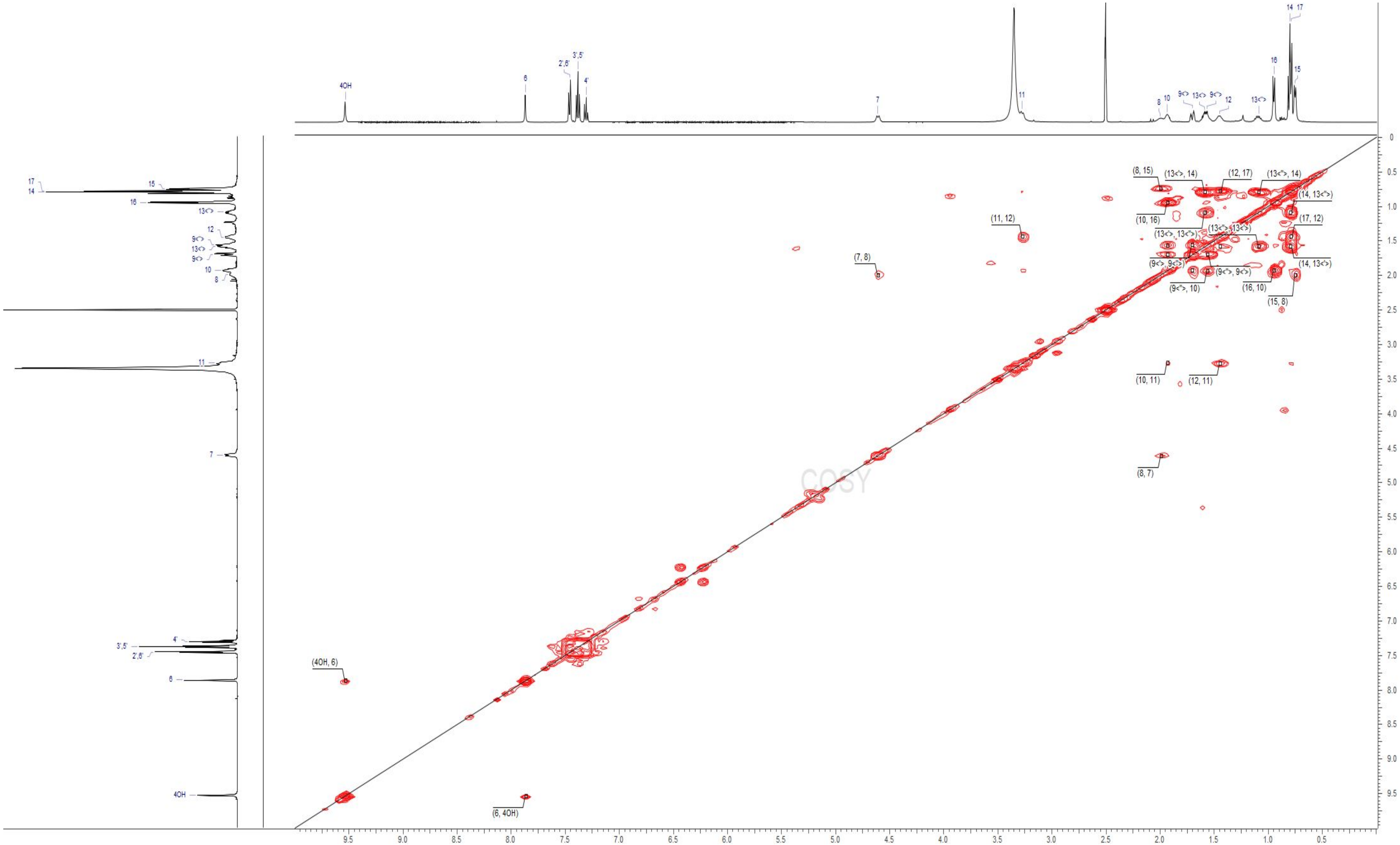

Figure S42. COSY NMR spectrum of **7** (700 MHz, DMSO-d_6).

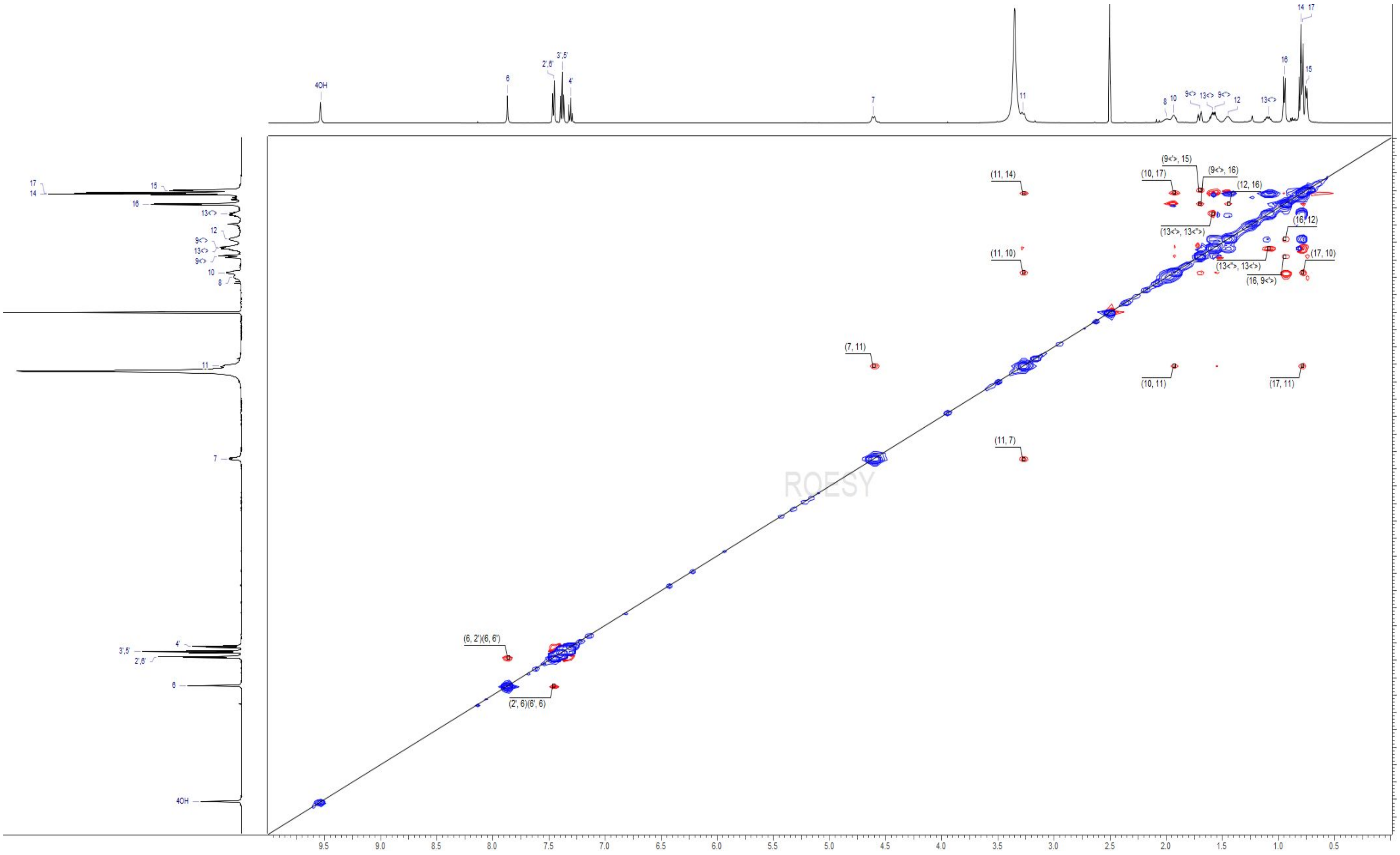

Figure S43. ROESY NMR spectrum of **7** (700 MHz, DMSO-d_6).

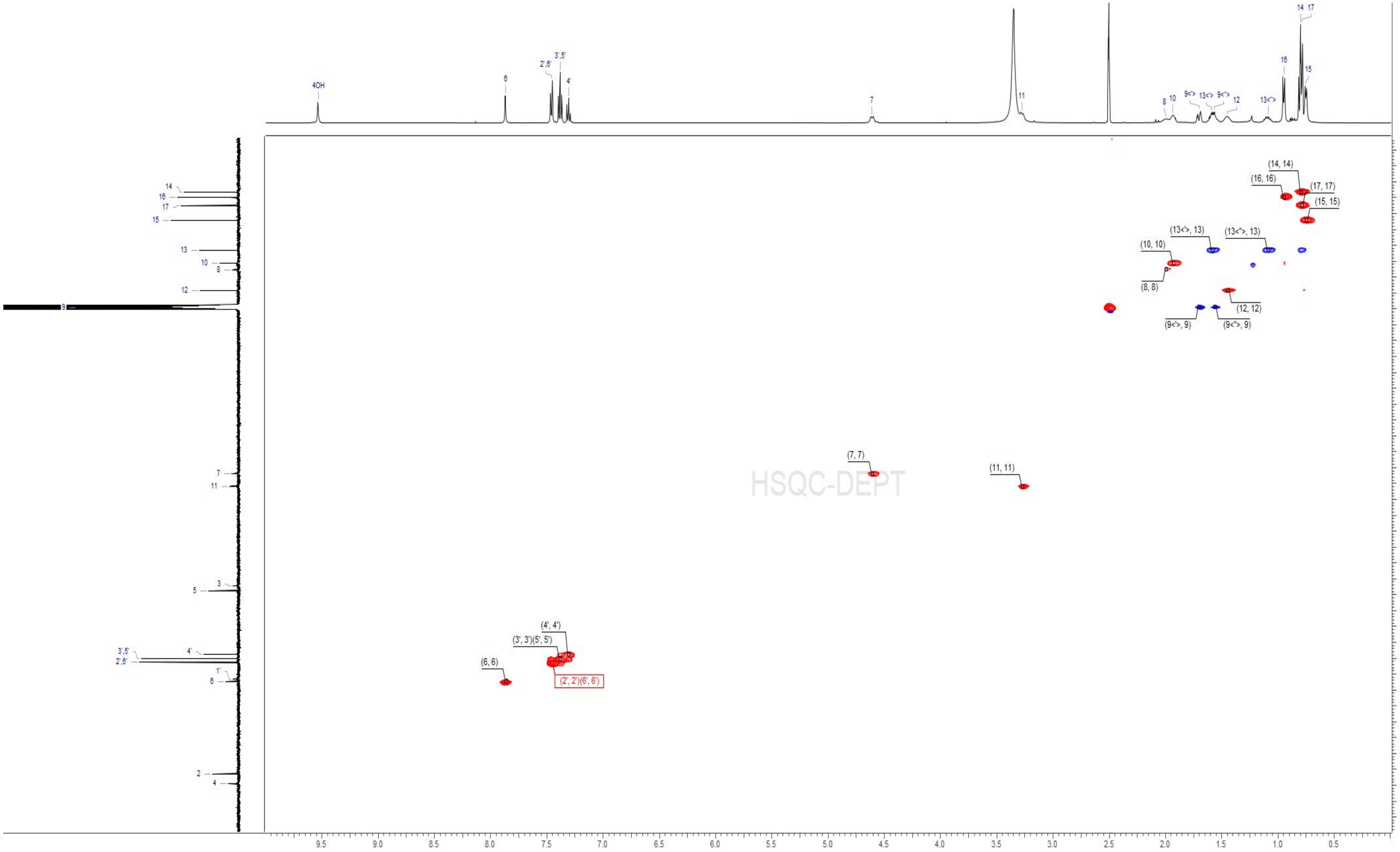

Figure S44. HSQC NMR spectrum of **7** (700 MHz, DMSO-d_6).

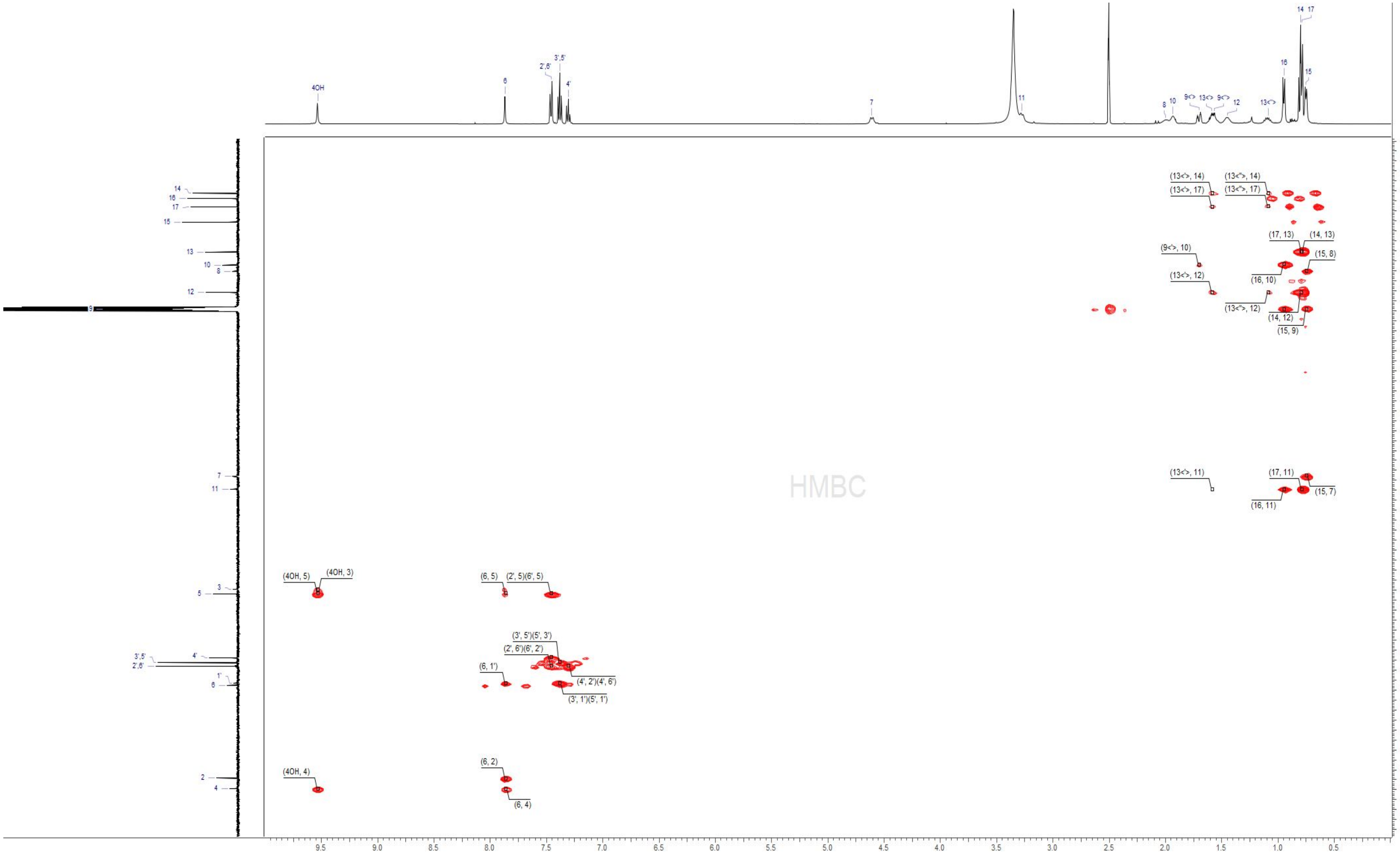

Figure S45. HMBC NMR spectrum of **7** (700 MHz, DMSO-d_6).

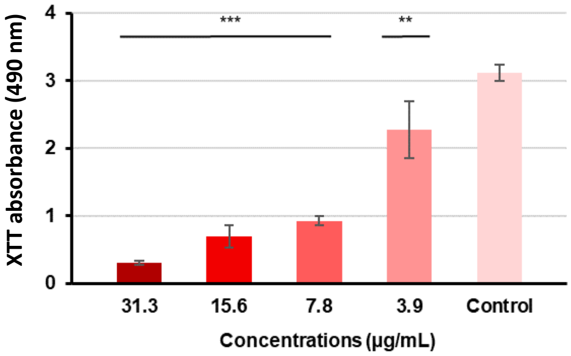

Figure S46. Effect of Acp A on the metabolic activity in preformed biofilm cells of *S. aureus* DSM 1104. Methanol as solvent control. Error bars indicate standard deviation, n=4. p values: ** $p < 0.01$, *** $p < 0.001$.

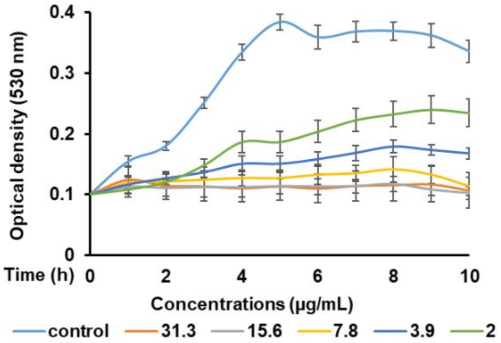

Figure S47. Effects of Acp A on the growth of *S. aureus* DSM 1104 planktonic cells. The absorption ($OD_{530\ nm}$) was measured in 90 min interval. Methanol was used as solvent control. Error bars indicate standard deviation, n=4.

References

1. K.-N. Truong, S. Ito, J. M. Wojciechowski, C. R. Göb, C. J. Schürmann, A. Yamano, M. Del Campo, E. Okunishi, Y. Aoyama, T. Mihira, N. Hosogi, J. Benet-Buchholz, E. C. Escudero-Adán, F. J. White, Joseph D. Ferrara, R. Bücker, Making the Most of ED Electron Diffraction: Best Practices to Handle a New Tool, *Symmetry,* **2023**, *15*, 1555. https://doi.org/10.3390/sym15081555
2. V. Petříček, L. Palatinus, J. Plášil, M. Dušek, Jana2020 – A new version of the crystallographic computing system JANA, *Z. Kristallogr. – Cryst. Mater.,* **2023**, *238*, 271–282. https://doi.org/10.1515/zkri-2023-0005
3. P. B. Klar, Y. Krysiak, H. Xu, G. Steciuk, J. Cho, X. Zou, L. Palatinus, Accurate structure models and absolute configuration determination using dynamical effects in continuous-rotation 3D electron diffraction data, *Nat. Chem.,* **2023**, *15*, 848–855. https://doi.org/10.1038/s41557-023-01186-1
